# Nanoparticle-mediated TRPV1 channel blockade amplifies cancer thermo-immunotherapy via heat shock factor 1 modulation

**DOI:** 10.1038/s41467-023-38128-x

**Published:** 2023-04-29

**Authors:** Ting Li, Shuhui Jiang, Ying Zhang, Jie Luo, Ming Li, Hengte Ke, Yibin Deng, Tao Yang, Xiaohui Sun, Huabing Chen

**Affiliations:** 1 Jiangsu Key Laboratory of Neuropsychiatric Diseases, and College of Pharmaceutical Sciences, Soochow University, Suzhou, 215123 China; 2 State Key Laboratory of Radiation Medicine and Protection, Soochow University, Suzhou, 215123 China

**Keywords:** Targeted therapies, Drug delivery, Target validation, Drug delivery, Nanoparticles

## Abstract

The survival of malignant tumors is highly dependent on their intrinsic self-defense pathways such as heat shock protein (HSP) during cancer therapy. However, precisely dismantling self-defenses to amplify antitumor potency remains unexplored. Herein, we demonstrate that nanoparticle-mediated transient receptor potential vanilloid member 1 (TRPV1) channel blockade potentiates thermo-immunotherapy via suppressing heat shock factor 1 (HSF1)-mediated dual self-defense pathways. TRPV1 blockade inhibits hyperthermia-induced calcium influx and subsequent nuclear translocation of HSF1, which selectively suppresses stressfully overexpressed HSP70 for enhancing thermotherapeutic efficacy against a variety of primary, metastatic and recurrent tumor models. Particularly, the suppression of HSF1 translocation further restrains the transforming growth factor β (TGFβ) pathway to degrade the tumor stroma, which improves the infiltration of antitumor therapeutics (e.g. anti-PD-L1 antibody) and immune cells into highly fibrotic and immunosuppressive pancreatic cancers. As a result, TRPV1 blockade retrieves thermo-immunotherapy with tumor-eradicable and immune memory effects. The nanoparticle-mediated TRPV1 blockade represents as an effective approach to dismantle self-defenses for potent cancer therapy.

## Abstract (web summary)

TRPV1 has been associated with proliferation and survival of tumors, and can be activated by heat and other stimuli. Here, the authors block TRPV1 using photothermal nanoparticles encapsulating a TRPV1 antagonist in different cancer types, which can enhance thermo-immunotherapy in pancreatic cancer models.

## Introduction

Intrinsic self-defense pathways of tumor cells severely impair therapeutic potencies^1–3^, leading to frequent tumor recurrence and metastasis. For instance, heat shock proteins (HSPs) in tumor cells are stressfully upregulated to repair cell injury upon abnormal hyperthermia that can often be afforded by photothermal conversion agents such as copper sulfide (CuS)-based nanoparticles under light irradiation^4–7^, or non-thermal factors such as oxidants and free radicals^8–10^, while transforming growth factor β (TGFβ) pathway at tumor causes inaccessibility of antitumor therapeutics through elevated cascade proliferation and activation of cancer-associated fibroblasts (CAFs) to induce excessive enrichment of extracellular matrix (ECM) in tumors^11,12^, further severely compromising antitumor efficacy of conventional therapeutic compounds against fibrotic tumors such as pancreatic ductal adenocarcinoma (PDAC) tumors, together with inevitable tumor recurrence and metastasis^11,13^. Although relevant small-molecule inhibitors have been extensively explored to dismantle self-defenses of tumors for improving therapeutic potencies^14–16^, such compounds still suffer from severe dose-limiting off-target toxicities owing to their indiscriminate suppression of stressfully overexpressed proteins in tumor and normally expressed proteins in healthy tissues that are necessarily involved in crucial intracellular events such as HSPs-assisted protein folding correction and TGFβ-mediated tissue repair^3,8^. Hence, exploring a specific and efficient tool to selectively dismantle self-defenses of tumors is still urgently demanded for safely amplifying cancer therapy.

Transient receptor potential vanilloid member 1 (TRPV1) channel as a calcium-permeable channel, is involved in a diversity of pathophysiological processes (e.g. temperature and pain perception) and can be activated by multiple physicochemical stimuli such as heat (>42 °C), low pH, pungent chemicals and endogenous nociception mediators^17–19^. Moreover, TRPV1 channel is found to be overexpressed in a variety of aggressive tumors including breast, lung, hepatocellular, colorectal and pancreatic tumors, and is closely associated with in vitro proliferation, migration and survival of tumor cells^20–22^. Inspired by the observations that TRPV1 channel was relevant to the expressions of HSP70 and TGFβ proteins^18,23,24^, we hypothesize that TRPV1 channel might be involved in the modulation of self-defense behaviors of tumor cells during cancer therapy.

In this work, we show that nanoparticle-mediated TRPV1 blockade selectively suppresses stressful HSP70 and TGFβ1 via effective modulation of heat shock factor 1 (HSF1) for augmented thermo-immunotherapy against highly malignant tumors. Via applying the A549-TRPV1 knockdown (A549-TRPV1 KD) tumor model and transcriptome analysis, TRPV1 blockade is found to specifically block calcium influx upon hyperthermia at tumor, and results in distinct inhibition of HSF1 nuclear translocation for selectively suppressing stressfully overexpressed HSP70 to reverse thermo-resistance. Furthermore, tumor-selective TRPV1 blockade using polymeric micelles incorporating both indocyanine green (ICG) and TRPV1 antagonist yields considerable antitumor potency against a variety of primary tumors (e.g. breast, liver, colorectal, and pancreatic tumors), metastatic tumors, and recurrent tumors under light exposure, together with superior safety. More importantly, the inhibition of HSF1 nuclear translocation from this TRPV1 blockade distinctly attenuates TGFβ1 for effective decomposition of ECM to improve the infiltration of antitumor therapeutics (e.g. anti-PD-L1 antibody, aPD-L1) and immune cells into highly fibrotic and immunosuppressive tumors such as PDAC model, eventually achieving synergistic thermo-immunotherapy against both subcutaneous and orthotopic tumor models through the reinvigorated immune responses and alleviated immunosuppression. Such nanoparticles-mediated TRPV1 blockade provides an emerging paradigm to dismantle self-defenses of tumors for safely amplifying cancer therapy against highly intractable tumors.

## Results

### TRPV1 blockade or knockdown enhances thermo-cytotoxicity

**Fig. 1:**
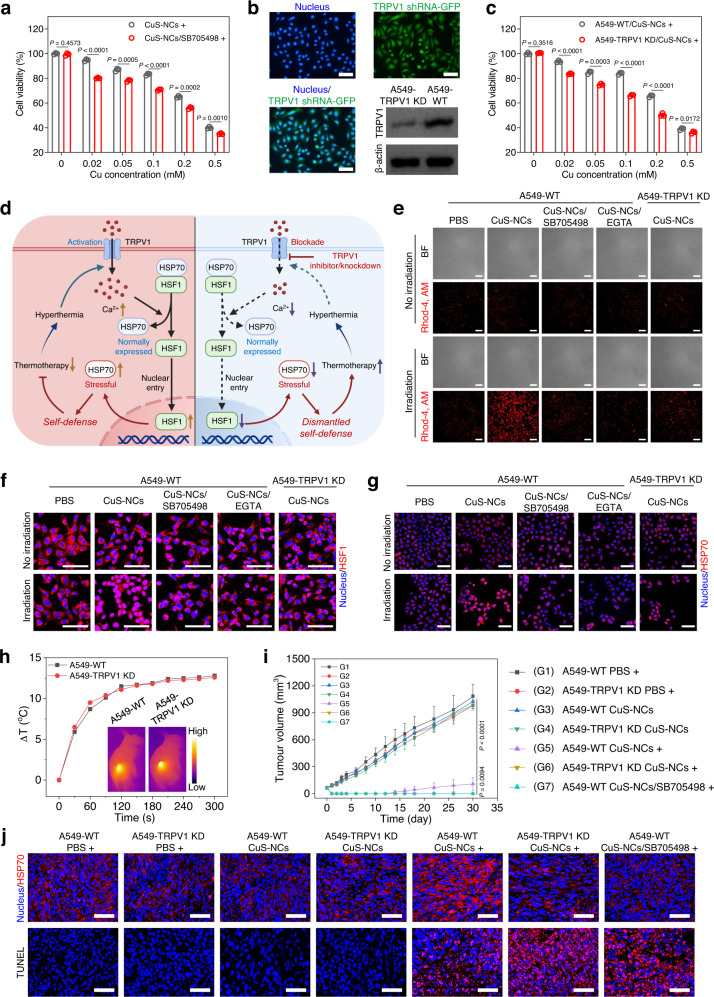
TRPV1 blockade or knockdown enhances thermotherapeutic efficacies via blocking Ca^2+^ influx and inhibiting HSF1 nuclear translocation to suppress stressful HSP70 upregulation. **a** Thermo-cytotoxicity of hyperthermia from CuS-NCs against A549-WT cells in the presence or absence of SB705498 (*n* = 3 biological replicates). **b** Fluorescent images and TRPV1 expression of stably transfected A549-TRPV1 knockdown cells (A549-TRPV1 KD). Scale bars, 100 μm. **c** Thermo-cytotoxicity of hyperthermia from CuS-NCs against A549-WT and A549-TRPV1 KD cells under light exposure (*n* = 3 biological replicates). **d** Schematic illustration of the synergistic mechanism of TRPV1 blockade with thermotherapy at tumor site. TRPV1 blockade effectively inhibits the calcium influx that induces heat shock factor 1 (HSF1) translocation into nucleus upon hyperthermia, leading to selective suppression of stressful HSP70 to dismantle the self-defense of tumor cells for preferable thermotherapeutic efficacy. Dash lines indicate the failure of downstream signals transduction. Ca^2+^ influx imaging (red) (**e**), CLSM images of HSF1 (red) (**f**) and HSP70 (red) (**g**) in A549-WT and A549-TRPV1 KD cells treated with or without hyperthermia from CuS-NCs in the presence or absence of SB705498 or EGTA. Scale bars, 100 μm. **h** Temperature elevation at tumor site of mice bearing subcutaneous A549-WT and A549-TRPV1 KD tumors at 24 h post-injection of CuS-NCs under light exposure. Inset is infrared thermography of mice at 5 min during irradiation (Color bar, Low represents 15 °C and High represents 50 °C). **i** Tumor volume of the mice bearing subcutaneous A549-WT and A549-TRPV1 KD tumors treated with hyperthermia from CuS-NCs together with intratumoral injection of SB705498 or not (*n* = 5 mice per group). **j** CLSM images of HSP70 (red) and TUNEL stainings (red) in the tumor sections from A549-WT or A549-TRPV1 KD tumor-bearing mice treated with hyperthermia from CuS-NCs together with intratumoral injection of SB705498 or not. Scale bars, 50 μm. Data are presented as mean ± SD (**a**, **c**, **i**). Statistical significance was determined by one-way ANOVA with Tukey’s post hoc test. The experiments for **b**, **e**, **f**, **g**, and **j** were repeated three times independently with similar results. Source data are provided as a Source Data file.

To generate potent hyperthermia for yielding cancer thermotherapy, the albumin nanoparticles caging copper sulfide nanocrystals (CuS-NCs) were constructed as a photothermal source as described previously^25,26^, which possessed the core size of 7.8 nm and hydrodynamic diameter of 25.4 nm (Supplementary Fig. 1a). These CuS-NCs exhibited the concentration-dependent temperature elevation with the increase of ∼30 °C during 300 s at the concentration of 1.0 mM Cu under near-infrared light irradiation, showing a distinct photothermal conversion capacity for causing hyperthermia owing to the notable near-infrared absorbance (Supplementary Fig. 1b, c). To evaluate the influence of TRPV1 blockade on hyperthermia-mediated cytotoxicity from CuS-NCs, a specific TRPV1 antagonist SB705498^27^, was applied to wild-type A549 (A549-WT) cells that were simultaneously treated with CuS-NCs as well, followed by 5 min light exposure at 1.5 W cm^−2^ and subsequent assessment of photocytotoxicity against A549-WT cells using MTT assay. The viability of A549-WT cells remained relatively unchanged after incubation with SB705498 or CuS-NCs without light exposure (Supplementary Fig. 2a, b). However, upon hyperthermia from CuS-NCs (0.2 mM), SB705498 (0–20 nM) displayed the concentration-dependent improvement of cytotoxicity, and 40 nM SB705498 caused no obvious increase of cytotoxicity as compared to 20 nM SB705498 (Supplementary Fig. 2c). In subsequent experiments, 20 nM SB705498 was used to block TRPV1 ion channels for potentiating thermo-cytotoxicity. In the presence of SB705498, the hyperthermia from CuS-NCs had the IC_50_ of 0.25 mM under light exposure, whereas a distinct increase of IC_50_ (0.39 mM) was observed in the absence of SB705498 under light exposure (Fig. 1a). Meanwhile, SB705498 was also found to amplify hyperthermia-mediated cell injury via increasing the cell culturing temperature (Supplementary Fig. 2d). These results suggest that the SB705498-mediated TRPV1 blockade accounts for the distinct improvement of thermo-cytotoxicity. Moreover, the 5-ethynyl-20-deoxyuridine (EdU) staining, which is frequently applied to detect proliferative cells during S phase through monitoring green fluorescence^28^, was performed to verify the ability of SB705498-mediated TRPV1 blockade to improve the thermo-cytotoxicity of CuS-NCs. Without light exposure, no matter combining SB705498 or not, CuS-NCs had no damage against A549-WT cell proliferation (Supplementary Fig. 3). On the contrary, upon light exposure, SB705498 distinctly promoted the hyperthermia-based cytotoxicity as evidenced by the lowest green fluorescence intensity, revealing less proliferative cells during S phase (Supplementary Fig. 3). Hence, the SB705498-mediated TRPV1 blockade dramatically improves the thermo-cytotoxicity upon hyperthermia.

To verify the synergy of TRPV1 blockade with the hyperthermia, we established the stable A549-TRPV1 knockdown (A549-TRPV1 KD) cells using TRPV1 shRNA with a green fluorescent protein (GFP) tag. As shown in Fig. 1b and Supplementary Fig. 4, more than 90% of the cells were GFP-positive, and the TRPV1 expression level in A549-TRPV1 KD cells was distinctly lower than that in A549-WT cells, confirming the effective knockdown of TRPV1. Then, the MTT assay showed that the hyperthermia from CuS-NCs had a 1.7-fold higher IC_50_ value of 0.35 mM in A549-WT cells than that in A549-TRPV1 KD cells (0.20 mM) (Fig. 1c). However, the overexpression of TRPV1 ion channels in A549 cells greatly decreased the thermo-cytotoxicity of CuS-NCs with the IC_50_ value of 0.45 mM (Supplementary Fig. 5a, b). These results suggest that the TRPV1 knockdown in tumor cells distinctly promotes the thermo-cytotoxicity. Since both A549-WT and A549-TRPV1 KD cells had similar cellular uptakes of CuS-NCs (Supplementary Fig. 5c), the knockdown of TRPV1 channel in tumor cells is reasonably involved in potentiating the hyperthermia-mediated thermo-cytotoxicity.

### TRPV1 blockade amplifies thermo-cytotoxicity via blocking Ca^2+^ influx and suppressing subsequent HSF1 nuclear translocation-mediated stressful HSP70 upregulation

Since TRPV1 blockade efficiently synergizes thermotherapeutic efficiency, we further explored the mechanism of SB705498 as a TRPV1 antagonist to evade thermo-resistance. TRPV1 channel, as a temperature-sensitive calcium ion channel that dominates calcium influx, participates in intracellular signal transduction^17,18^. We thus hypothesized that Ca^2+^ influx might play a vital role during thermotherapy, which is probably associated with HSF1 nuclear translocation and subsequent stressful HSP70 upregulation (Fig. 1d)^29–31^. To clearly observe the influx of Ca^2+^ after various treatments, a red fluorescent Ca^2+^-binding dye (CalciFluor^TM^ Rhod-4, AM) was used. The hyperthermia from CuS-NCs induced a sharp increase of intracellular Ca^2+^ in A549-WT cells as indicated by the potently improved red fluorescence intensity (7.9-fold over that of PBS group), while TRPV1 blockade by SB705498 or TRPV1 knockdown was found to distinctly inhibit this intracellular Ca^2+^ increase (Fig. 1e and Supplementary Fig. 6a). To further validate whether the Ca^2+^ influx depends on extracellular Ca^2+^, we utilized an extracellular Ca^2+^ chelator, ethylene glycol-bis-(2-aminoethylether)-N,N,N’,N’-tetraacetic acid (EGTA), to scavenge extracellular Ca^2+^ in the medium. Clearly, scavenging extracellular Ca^2+^ led to negligible increase of red fluorescence, suggesting a specific dependence of Ca^2+^ influx on extracellular Ca^2+^ (Fig. 1e and Supplementary Fig. 6a). Moreover, the flow cytometric analysis of red fluorescence from intracellular Ca^2+^ using CalciFluor^TM^ Rhod-4, AM probe further confirmed the ability of TRPV1 blockade or knockdown to suppress Ca^2+^ influx (Supplementary Fig. 6b, c). Hence, the hyperthermia sensitively activates TRPV1 channel-mediated Ca^2+^ influx (Fig. 1d), which is distinctly disadvantageous to thermo-cytotoxicity, while this Ca^2+^ influx is also able to be effectively inhibited by TRPV1 blockade.

We further demonstrated the influence of Ca^2+^ influx on the thermo-cytotoxicity from CuS-NCs using the MTT assay, in which non-toxic EGTA as calcium scavenger at the dose of 2.0 mM caused the IC_50_ of ~0.24 mM under hyperthermia from CuS-NCs (Supplementary Fig. 7), being preferable to the cytotoxicity (~0.35 mM) from the hyperthermia alone. Hence, scavenging extracellular Ca^2+^ using EGTA displays a similar behavior to non-toxic SB705498 as indicated in Fig. 1a. Afterwards, the EdU staining further revealed that the hyperthermia in the presence of EGTA resulted in a preferable inhibitory effect on cell proliferation as compared to hyperthermia alone under light exposure (Supplementary Fig. 8), confirming that blocking Ca^2+^ influx through TRPV1 channel is able to enhance the thermo-cytotoxicity.

Afterward, the flow cytometry was also utilized to evaluate the apoptosis of A549-WT cells under different treatments. The CuS-NCs-mediated hyperthermia alone caused the apoptosis level of 62.3%, while effective inhibition of Ca^2+^ influx through TRPV1 antagonist SB705498 or calcium chelator EGTA led to an apparent increase of apoptosis (75.2%) under hyperthermia from CuS-NCs upon light exposure (Supplementary Fig. 9), indicating the hyperthermia-activated Ca^2+^ influx distinctly impair the thermotherapeutic efficiency. In addition, the western blot was also utilized to validate the apoptotic behavior, and the enhanced expression of cleaved caspase-3 was observed for A549-WT cells under CuS-NCs-mediated hyperthermia when combining with SB705498, EGTA, or TPRV1 knockdown as compared to that under hyperthermia alone (Supplementary Fig. 10). Notably, TRPV1 blockade synergizes thermo-cytotoxicity and promotes tumor cells apoptosis *via* efficiently blocking Ca^2+^ influx.

To further unravel the synergistic mechanism caused by inhibition of Ca^2+^ influx upon hyperthermia, in which Ca^2+^ is presumably associated with cellular heat shock response including the translocation of heat shock transcription factor 1 (HSF1) into nucleus, and subsequent transcription and final translation of stressful HSP70^29,32^. Thus, we applied the immunofluorescence staining to assess the role of TRPV1 blockade in interfering the intranuclear HSF1 translocation and intracellular HSP70 expression in tumor cells under hyperthermia caused by CuS-NCs. The hyperthermia alone resulted in distinct nuclear translocation of HSF1 with 84.6% co-localization rate of HSF1 and nucleus (Fig. 1f and Supplementary Fig. 11a), and subsequent overexpression of HSP70 in A549-WT cells (Fig. 1g and Supplementary 11b), indicating that the hyperthermia specifically activates the stressful HSP70 expression. Meanwhile, SB705498 co-incubation or TRPV1 knockdown was found to apparently block HSF1 nuclear translocation with the co-localization rates of 24.4% and 35.6% (Fig. 1f and Supplementary Fig. 11a), respectively, and suppress stressful HSP70 upregulation under hyperthermia, as evidenced by almost unchanged fluorescence signals that were related to intranuclear HSF1 and intracellular HSP70 levels (Fig. 1g and Supplementary Fig. 11b), respectively. In particular, to verify whether the blockade of Ca^2+^ influx accounts for the suppression of HSF1 translocation and stressful HSP70 upregulation, EGTA (2.0 mM) was also applied to scavenge Ca^2+^ in the medium. EGTA distinctly suppressed the intranuclear translocation of HSF1 and stressful HSP70 upregulation in A549-WT cells under hyperthermia from CuS-NCs upon light irradiation (Fig. 1f, g and Supplementary Fig. 11), resembling the behavior of SB705498. Moreover, the ability of TRPV1 blockade to suppress intranuclear translocation of HSF1 and subsequent stressful HSP70 upregulation upon hyperthermia was also verified using the western blot. Both TRPV1 blockade and knockdown led to the potent decreases of intranuclear HSF1 expression as compared to that of A549-WT cells receiving hyperthermia alone from CuS-NCs (Supplementary Fig. 12), accounting for effective downregulation of stressful HSP70 (Supplementary Fig. 13). These results demonstrate the ability of TRPV1 blockade or knockdown to suppress intranuclear translocation of HSF1 for downregulating stressful HSP70, finally synergizing thermotherapy.

### TRPV1 blockade or knockdown potently amplifies thermotherapeutic efficacy in vivo

To investigate the synergistic effect of SB705498-mediated TRPV1 blockade on the thermotherapeutic potency in vivo, A549-WT cells or A549-TRPV1 KD cells were injected subcutaneously into the nude mice to establish A549-WT tumor or A549-TRPV1 KD tumor models, respectively. Notably, with similar tumor accumulation of ~7.2% ID g^−1^ at 24 h post-injection in the mice bearing A549-WT or A549-TRPV1 KD tumors (Supplementary Fig. 14), CuS-NCs at the dose of 30.0 µmol kg^−1^ Cu caused the temperature increases (ΔT) of ~12.8 °C and ~12.6 °C during 5 min at A549-WT and A549-TRPV1 KD tumors under light exposure at 24 h post-injection (Fig. 1h and Supplementary Fig. 15), respectively, indicating that CuS-NCs cause similar hyperthermia at both tumor models.

To trigger in vivo thermotherapy, CuS-NCs were intravenously injected into the mice bearing subcutaneous A549-WT and A549-TRPV1 KD tumors at the dose of 30.0 µmol kg^−1^, and then the mice were exposed to 785 nm light irradiation (5 min, 1.5 W cm^−2^) at 24 h post-injection. Meanwhile, SB705498 (1.0 mg kg^−1^) was intratumorally injected at 30 min pre-irradiation for inducing TRPV1 blockade in A549-WT tumor models followed by tumor volumes monitoring during 30 days post-injection (Supplementary Fig. 16). The mice bearing A549-WT tumor and A549-TRPV1 KD tumor in the PBS groups exhibited similar tumor growth profiles with ~15.0-fold increases during 30 days (Fig. 1i and Supplementary Fig. 17a), demonstrating that TRPV1 channel itself has no influence on tumor growth under non-hyperthermia conditions. Probably, the negligible influence of TRPV1 knockdown on tumor growth might arise from the relatively short monitoring period^33^. Interestingly, upon hyperthermia from CuS-NCs, the A549-WT tumors were rapidly ablated, but subsequently displayed apparent recurrence at 14 days post-irradiation (Fig. 1i and Supplementary Fig. 17a), suggesting that the thermotherapy alone is difficult to eradicate tumors due to the resistance of tumor cells to the thermotherapy^34^. Importantly, upon TRPV1 blockade or knockdown, the hyperthermia from CuS-NCs completely eliminated the tumors without any recurrence and negligible body weight variation (Fig. 1i and Supplementary Fig. 17), which was further verified by the severe histological damage against the tumors and potent inhibition on cell proliferation through hematoxylin and eosin (H&E) staining and Ki67 staining (Supplementary Fig. 18). In particular, both TRPV1 blockade and knockdown caused a similar synergistic ability, confirming that the TRPV1 blockade plays a crucial role in combating the thermal resistance in cancer thermotherapy. In addition, CuS-NCs also had no influence on the tumor growth in both two tumor models in the absence of light irradiation (Fig. 1i and Supplementary Fig. 17a–c), indicating that the hyperthermia from light-activatable CuS-NCs ensures selective thermotherapy against tumor cells.

We next investigated the in vivo expression of stressful HSP70 in both A549-WT and A549-TRPV1 KD tumor models under hyperthermia using the immunofluorescence staining. The distinct red fluorescence from HSP70 expression of tumor section was observed for CuS-NCs-mediated hyperthermia alone, which was distinctly higher than those in the absence of hyperthermia, indicating an in vivo generation of stressful HSP70 at tumor upon hyperthermia (Fig. 1j). Importantly, the TRPV1 blockade using SB705498 or TRPV1 knockdown effectively inhibited the in vivo stressful HSP70 expression at tumor section, together without distinct influence on normally expressed HSP70 (Fig. 1j). It suggests that the TRPV1 blockade specifically suppresses the in vivo expression of stressful HSP70 at tumor site. The TUNEL staining was also utilized to assess the effect of TRPV1 blockade on the in vivo damages against the tumors from A549-WT and A549-TRPV1 KD tumor models upon thermotherapy. Ignorable red fluorescence was observed for TRPV1 knockdown in the absence of hyperthermia, indicating a non-toxic property of TRPV1 channel itself on tumor cell apoptosis (Fig. 1j). Interestingly, the hyperthermia alone caused an obvious apoptotic behavior at the tumor section as verified by the enhanced red fluorescence, whereas SB705498 or TRPV1 knockdown further amplified the apoptotic levels at the tumor sections caused by the hyperthermia (Fig. 1j). The SB705498-mediated TRPV1 blockade boosts thermotherapeutic damage by blocking Ca^2+^ influx to restrict nuclear translocation of HSF1 and stressful HSP70 overexpression (Fig. 1d).

### Transcriptome analyses reveal the potential synergy between TRPV1 blockade and thermotherapy

**Fig. 2:**
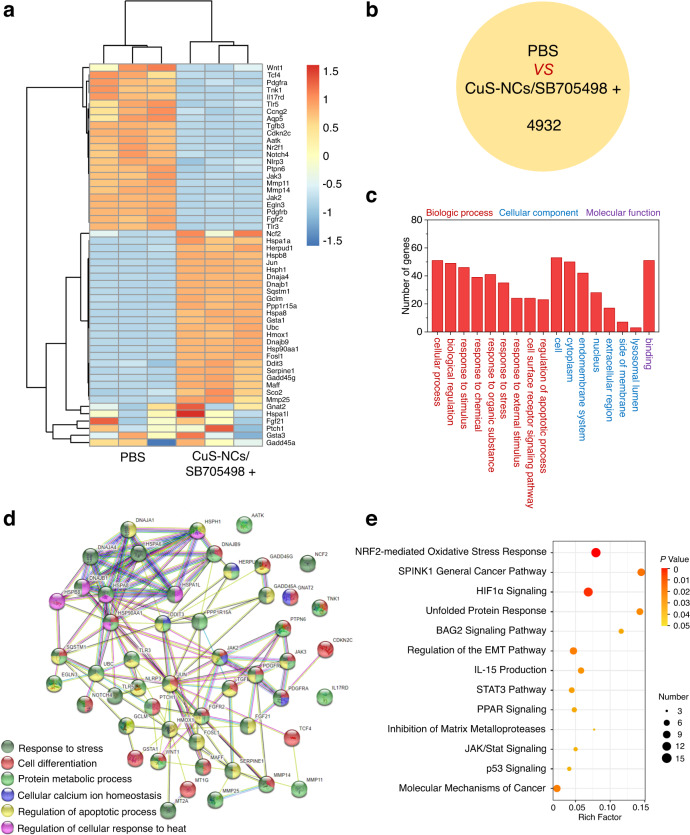
Transcriptome analyses of the synergistic effect of TRPV1 blockade with thermotherapy. **a** Heat map of prominently upregulated and downregulated genes in A549-WT cells treated with the hyperthermia from CuS-NCs (0.2 mM) under light irradiation at the dose of 20.0 nM SB705498 (*n* = 3, fold change ≥ 2 and *p* < 0.05). **b** The number of variable primary transcripts between PBS and TRPV1 blockade-synergized thermotherapy. **c** Gene Ontology (GO) analysis of variable genes associated with the biologic process (red font), cellular component (blue font) and molecular functions (purple font) between PBS and TRPV1 blockade-synergized thermotherapy. **d** Search Tool for the Retrieval of Interacting Genes/Proteins (STRING) algorithm analysis of functional interaction network between PBS and TRPV1 blockade-synergized thermotherapy. **e** Kyoto Encyclopedia of Genes and Genomes (KEGG) pathway analysis of variable genes in A549-WT cells treated with TRPV1 blockade-synergized thermotherapy. The measurements were repeated 3 times. Statistical significance was determined by two-tailed Student’s *t* test (**a**) and two-tailed Fisher’s exact test (**e**). The raw RNA-sequencing data have been deposited in the Gene Expression Omnibus under the accession number GSE199299.

To thoroughly investigate the influence of TRPV1 blockade on thermotherapy, the transcriptome analysis of A549-WT cells was conducted after treatment with both CuS-NCs-mediated hyperthermia and SB705498. Distinctly, 23 upregulated genes and 30 downregulated genes were found for the combination of hyperthermia and TRPV1 blockade as compared to PBS according to principal component analysis and cluster analysis (Fig. 2a, *P* < 0.05 and fold change ≥ 2). As shown in Fig. 2b, a distinct discrepancy of 4932 primary transcripts, being responsible for regulating downstream genes expression, was observed for the synergy of TRPV1 with hyperthermia as compared to PBS. Moreover, Gene Ontology (GO) analysis revealed that these 53 distinctly variable genes were closely associated with the biological process, molecular function and cellular component (Fig. 2c). Among them, those genes related to binding might be responsible for cancer cell apoptosis and migration, revealing a potent ability of TRPV1 blockade to synergize thermotherapy. In Fig. 2d, the Search Tool for the Retrieval of Interacting Genes/Proteins (STRING) network analysis was applied to determine different protein-protein interactions, and six types of functional proteins were found to be mainly involved in cellular calcium ion homeostasis, cellular heat response, cell differentiation, protein metabolic process, and regulation of apoptotic process. Moreover, Kyoto Encyclopedia of Genes and Genomes (KEGG) pathway analysis indicated that these variable genes are particularly associated with several specific intracellular pathways including Nrf2-mediated oxidative stress response, relevant to external hyperthermia stimulus^35^, p53 signaling^36^, regulation of epithelial-mesenchymal transition (EMT) pathway and inhibition of matrix metalloproteases that are closely participated in tumor migration^37,38^, suggesting the effectiveness of TRPV1 blockade to potently regulate cell apoptosis and potentially suppress tumor metastasis (Fig. 2e). Hence, the transcriptome analysis suggests that the synergy between TRPV1 blockade and thermotherapy is highly relevant to apoptosis, migration and metastasis.

### Nanoparticles-mediated targeted delivery of TRPV1 antagonist for selective TRPV1 blockade-synergized thermo-cytotoxicity

**Fig. 3:**
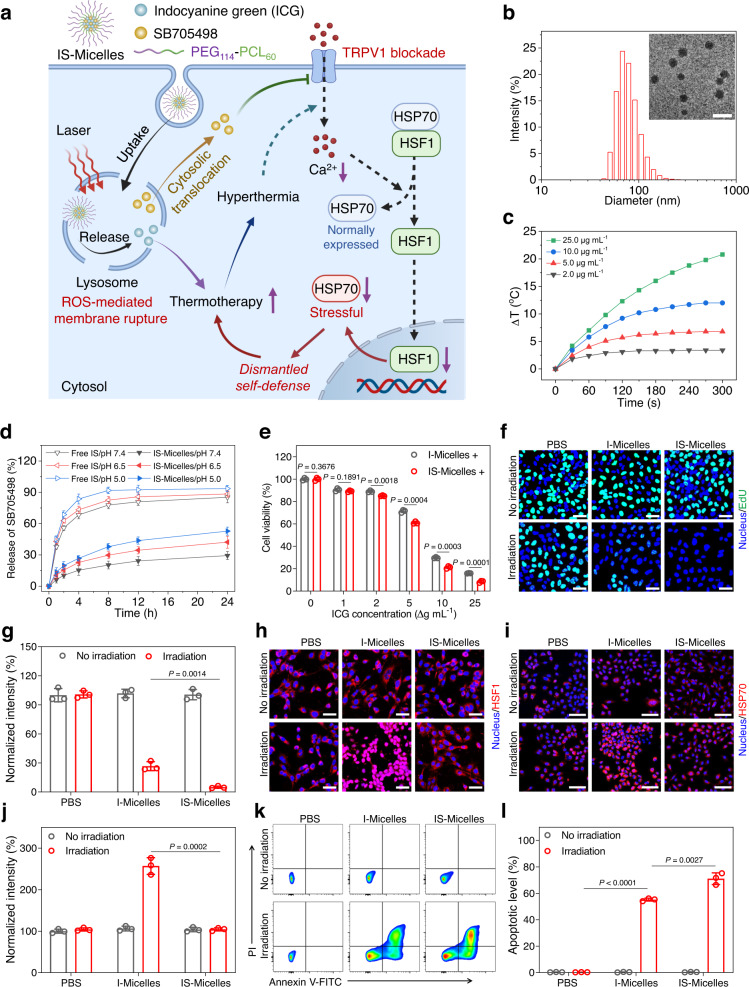
Co-delivery of TRPV1 antagonist and photothermal agent using polymeric micelles achieves TRPV1 blockade-synergized thermo-cytotoxicity. **a** Schematic illustration of tumor-targeted delivery of TRPV1 antagonist (SB705498) and photothermal agent (ICG) using IS-Micelles, followed by pH-responsive drug release and ROS-mediated cytoplasmic translocation of TRPV1 antagonist to facilitate TRPV1 blockade for synergizing thermotherapy. Dash lines indicate the failure of downstream signals transduction upon blockade of hyperthermia-activated TRPV1 channel. **b** Size distribution of IS-Micelles using dynamic light scattering. Inset is transmission electron microscopy (TEM) image of IS-Micelles. Scale bar, 100 nm. **c** Temperature elevation profiles of IS-Micelles at the concentrations of 2.0, 5.0, 10.0 and 25.0 µg mL^−1^ ICG under light exposure. **d** Release profiles of SB705498 from IS-Micelles at pH 7.4, 6.5 and 5.0 (*n* = 3 independent experiments). **e** Thermo-cytotoxicity of IS-Micelles and I-Micelles against 4T1-Luc cells under light irradiation (+) (n = 3 biological replicates). CLSM images (**f**) and corresponding fluorescence intensity analysis (**g**) of EdU (green) in 4T1-Luc tumor cells treated with I-Micelles or IS-Micelles under light irradiation or not (*n* = 3 independent experiments). Scale bars, 50 μm. **h** CLSM images of HSF1 (red) in 4T1-Luc tumor cells treated with I-Micelles or IS-Micelles in the presence or absence of light irradiation. Scale bars, 50 μm. CLSM images (**i**) and corresponding fluorescence intensity analysis (**j**) of HSP70 (red) in 4T1-Luc tumor cells treated with I-Micelles or IS-Micelles under light irradiation or not (*n* = 3 independent experiments). Scale bars, 100 μm. Representative flow cytometry dot plot (**k**) and apoptotic levels (**l**) in 4T1-Luc tumor cells treated with I-Micelles or IS-Micelles under light irradiation or not using Annexin V-FITC/PI staining (*n* = 3 biological replicates). Data are presented as mean ± SD (**d**, **e**, **g**, **j**, **l**). Statistical significance was determined by one-way ANOVA with Tukey’s post hoc test. The experiments for **b**, **c**, **f**, **h**, and **i** were repeated three times independently with similar results. Source data are provided as a Source Data file.

TRPV1 blockade was identified to induce the enhanced cancer thermotherapy by the combination of intratumoral TRPV1 antagonist (SB705498) with CuS-NCs-triggered hyperthermia as indicated above. To achieve reasonable synergy of TRPV1 antagonist with thermotherapy, we thus constructed the ICG/SB705498-loaded polymeric micelles (IS-Micelles, with drug loading efficiency of 7.5% ICG and 5.0% SB705498, respectively) using poly(ethylene glycol)_114_-*b*-poly(caprolactone)_60_ (PEG_114_-PCL_60_) as the vehicle^39,40^, which were expected to synchronously co-deliver TRPV1 antagonist and photothermal ICG into the tumors via intravenous injection (Fig. 3a). IS-Micelles possessed a transmission electron microscopy (TEM) diameter of ~42.8 nm and hydrodynamic diameter of ~75.6 nm (Fig. 3b), respectively. ICG-loaded polymeric micelles (I-Micelles with a diameter of 43.2 nm in TEM, Supplementary Fig. 19) were fabricated as the control with the function of hyperthermia alone. To confirm the ability of IS-Micelles to yield hyperthermia, the temperature elevation was monitored during 5 min under light exposure (785 nm, 1.5 W cm^−2^). These micelles exhibited apparent concentration-dependent temperature elevations (Fig. 3c), reasonably owing to their high photothermal conversion efficiency of 22.7% (Supplementary Fig. 20). Moreover, IS-Micelles exhibited a relatively low release of SB705498 at pH 7.4, while the increased releases of SB705498 from IS-Micelles were observed at pH 6.5 and 5.5 due to the enhanced solubility of SB705498 at acidic pH (Fig. 3d), thereby contributing to tumor microenvironment-activatable release for effective TRPV1 blockade.

We then evaluated the cellular uptake of IS-Micelles by 4T1-Luc triple-negative breast cancer (TNBC) cells that possess a similar expression level of TRPV1 channel to A549-WT cells (Supplementary Fig. 21). IS-Micelles exhibited the time-dependent uptakes of ICG through the clathrin-mediated endocytic pathway as evidenced by the decreased cellular uptake of ICG after chlorpromazine treatment (Supplementary Fig. 22). In particular, we further assessed the intracellular distribution of IS-Micelles using confocal laser scanning microscopy (CLSM). A co-localization rate of 94.8% with the lysosomes was observed for IS-Micelles, but was decreased to 18.9% under light exposure owing to the rupture of lysosomal membrane and subsequent cytoplasmic translocation (Supplementary Fig. 23). Reasonably, efficient generation of reactive oxygen species (ROS) from IS-Micelles accounts for the disruption of lysosome membrane, as confirmed by their dihydroethidium (DHE) staining with distinct red fluorescence after incubation of IS-Micelles under light exposure (Supplementary Fig. 24a). Moreover, the ability of IS-Micelles to disrupt lysosomal membrane was further verified using the acridine orange (AO) staining with the total disappearance of the red fluorescence in the lysosomes upon light irradiation (Supplementary Fig. 24b). To verify the ability of IS-Micelles to block TRPV1, we firstly employed Fluo-8 to monitor intracellular Ca^2+^ accumulation using CLSM. Apparently, IS-Micelles displayed much lower green fluorescence signals as compared to I-Micelles under light irradiation due to the inhibition of Ca^2+^ influx through TRPV1 blockade (Supplementary Fig. 25). Hence, the effective cellular uptake, cytoplasmic translocation, and pH-responsive release potentially promote the accessibility of SB705498 to TRPV1 channel from IS-Micelles for effective TRPV1 blockade.

To confirm the ability of TRPV1 blockade from IS-Micelles to synergize hyperthermia, the thermo-cytotoxicity was evaluated via the MTT assay with the IC_50_ of ∼6.8 µg mL^−1^ and ∼8.4 µg mL^−1^ for IS-Micelles and I-Micelles (Fig. 3e and Supplementary Fig. 26), respectively. The EdU staining further indicated the preferable inhibitory effect on the cell proliferation of IS-Micelles as compared to that of I-Micelles under light irradiation (Fig. 3f, g). Moreover, we applied the immunofluorescence staining of HSF1 and HSP expressions to validate whether the targeted co-delivery strategy could effectively overcome the thermo-resistance during thermotherapy. Clearly, negligible red fluorescence of HSF1 in the nucleus was observed for the hyperthermia from IS-Micelles under light irradiation, whereas I-Micelles as a control showed the distinct cytoplasmic translocation of HSF1 into the nucleus due to the lack of TRPV1 blockade (Fig. 3h), thus leading to obvious upregulation of stressful HSP70 (Fig. 3i, j). In contrast, through the efficient co-delivery of TRPV1 antagonist, the tumor cells in the group of IS-Micelles displayed no distinct change of HSP70 expression as compared to that of PBS upon irradiation (Fig. 3i, j), suggesting that IS-Micelles are able to block stressful HSP70 overexpression through valid TRPV1 blockade for surmounting thermo-resistance. Furthermore, the flow cytometry was employed to evaluate the apoptotic behavior via Annexin V-FITC/PI staining. IS-Micelles resulted in the apoptosis of ~71.7%, showing a preferable thermo-cytotoxicity as compared to that (59.4%) of I-Micelles with hyperthermia alone (Fig. 3k, l), which was also evidenced by the reduced HSP70 expression and distinctly upregulated cleaved caspase-3 in western blot (Supplementary Fig. 27). These data clearly indicate that the co-delivery strategy of IS-Micelles successfully facilitates TRPV1 blockade to overcome thermo-resistance for potentiating their thermo-cytotoxicity through the improved accessibility of SB705498 to TRPV1 channel.

### TRPV1 blockade-synergized thermotherapy yields in vivo suppression against primary, metastatic and recurrent TNBC tumors through targeted delivery of IS-Micelles

**Fig. 4:**
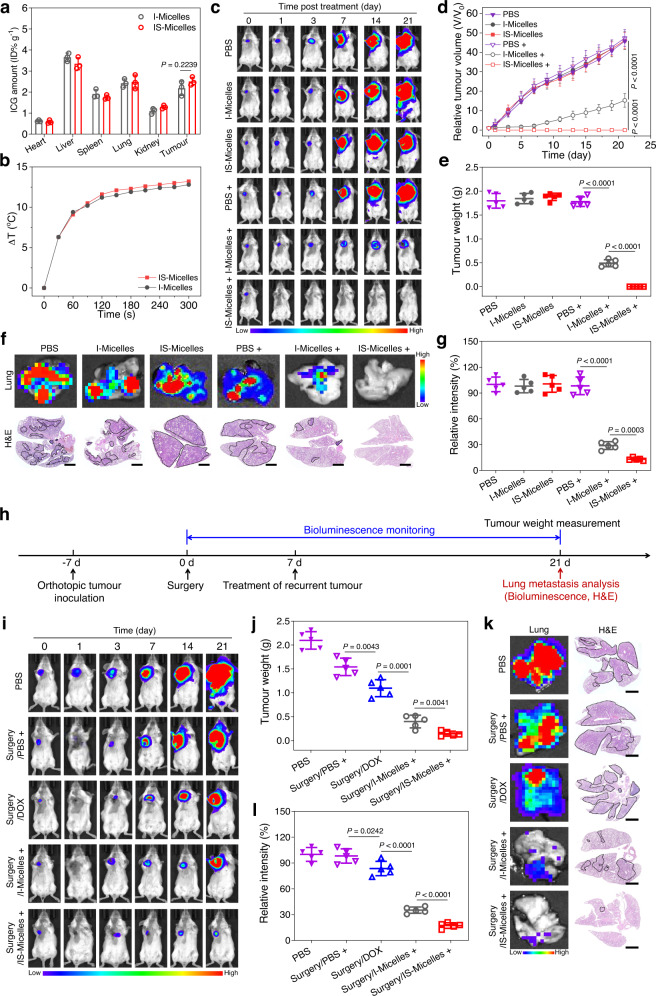
TRPV1 blockade-synergized thermotherapy effectively suppresses primary, metastatic and recurrent TNBC tumors using IS-Micelles. **a** Biodistribution of I-Micelles and IS-Micelles in orthotopic 4T1-Luc tumors at 24 h post-injection (*n* = 3 mice per group). **b** Temperature elevation at tumor site of mice bearing orthotopic 4T1-Luc tumors at 24 h post-injection of I-Micelles or IS-Micelles under light irradiation. Bioluminescence images (**c**), relative tumor volumes (**d**) and tumor weights (**e**) of the mice bearing orthotopic 4T1-Luc tumors treated with IS-Micelles or I-Micelles under light irradiation (+) or not during 21 days (*n* = 5 mice per group, color bar, Low represents 1000 a.u. and High represents 30000 a.u.). Bioluminescence images (up), H&E images (down) (**f**) and relative bioluminescence intensities (**g**) of the lung metastasis from the mice bearing orthotopic 4T1-Luc tumors at the end of the experiment as indicated in **c** (*n* = 5 mice per group, color bar, Low represents 100 a.u. and High represents 800 a.u.). Areas marked by black dash lines indicate the metastatic regions. Scale bars, 2 mm. **h** Timeline for the treatment of recurrent TNBC tumor models suffering from surgical failure. Bioluminescence images (**i**) and tumor weights (**j**) of the mice bearing orthotopic 4T1-Luc breast tumors treated with initial surgery, followed by subsequent treatments with IS-Micelles under light irradiation (+), I-Micelles under light irradiation (+) and DOX, respectively (*n* = 5 mice per group, color bar, Low represents 1000 a.u. and High represents 30000 a.u.). Bioluminescence images (left), H&E images (right) (**k**) and relative bioluminescence intensities (**l**) of the lung metastasis from the mice at the end of the experiment as indicated in **i** (*n* = 5 mice per group, color bar, Low represents 100 a.u. and High represents 800 a.u.). Areas marked by black dash lines indicate the metastatic regions. Scale bars, 2 mm. Data are presented as mean ± SD (**a**, **d**, **e**, **g**, **j**, **l**). Statistical significance was determined by one-way ANOVA with Tukey’s post hoc test. Source data are provided as a Source Data file.

To demonstrate the synergistic antitumor efficacy of IS-Micelles against TNBC, we firstly evaluated their biodistribution in the mice bearing orthotopic 4T1-Luc breast tumors via intravenous injection of IS-Micelles at the dose of 7.5 mg kg^−1^ ICG and 5.0 mg kg^−1^ SB705498. Both of IS-Micelles and I-Micelles exhibited the effective tumor biodistribution of ∼2.6% ID g^−1^ at tumor site after 24 h post-injection (Fig. 4a), accounting for comparable hyperthermia of ~13.0 °C under light irradiation (Fig. 4b and Supplementary Fig. 28). We then evaluated the in vivo tumoral microdistribution of IS-Micelles through the tumor cell membrane staining using primary anti-*N*-cadherin primary antibody and Alexa Fluor 488 conjugated secondary antibody. A uniform distribution at tumor stroma and intracellular space was observed for IS-Micelles (Supplementary Fig. 29), confirming deep and uniform penetration of TRPV1 antagonist and photothermal agent into tumor. Moreover, we investigated the penetration depth of IS-Micelles via immunofluorescence staining using platelet and endothelial cell adhesion molecule 1 (PECAM-1) primary antibody and Alexa Fluor 488 conjugated secondary antibody. IS-Micelles exhibited considerable extravasation and deep penetration depth of ~100 μm in small 4T1 tumors (~75 mm^3^) due to their suitable size distribution (Supplementary Fig. 30). Owing to the abundant blood vessels in highly permeable 4T1 tumors^41^, IS-Micelles still displayed uniform and deep penetration in large 4T1 tumors (~250 mm^3^), being comparable to that of IS-Micelles in small 4T1 tumors (Supplementary Fig. 30).

To explore the in vivo synergy of TRPV1 blockade with thermotherapy, IS-Micelles were administrated into the orthotopic 4T1-Luc breast tumors-bearing mice at a single dose of 7.5 mg kg^−1^ ICG and 5.0 mg kg^−1^ SB705498, followed by light exposure (785 nm, 1.5 W cm^−2^) for 5 min and subsequent measurements of tumor volumes during 21 days post-injection. The ~47.5-fold increases of tumor volumes were observed for PBS regardless of light exposure, and the mice in both IS-Micelles and I-Micelles groups without irradiation also showed similar tumor growth to PBS, as validated by their bioluminescence images (Fig. 4c, d). Importantly, IS-Micelles resulted in the complete tumor eradication without any regrowth upon irradiation, while I-Micelles with hyperthermia alone displayed an increase of ~14.9-fold tumor volume due to rapid tumor recurrence at 7 days post-injection (Fig. 4c, d). Moreover, the tumor weights of the mice at the end of the experiment further confirmed the preferable antitumor efficacy of IS-Micelles as compared to I-Micelles (Fig. 4e). Notably, the strategy of TRPV1 blockade shows a distinct advantage in the eradication of the tumor cells surviving from hyperthermia upon irradiating IS-Micelles at tumor. To further confirm the tumor damage caused by the synergy of TRPV1 blockade with thermotherapy, 4T1-Luc breast tumors suffering from different treatments were collected at 6 h post-irradiation for subsequent histological, Ki67 and TUNEL staining. Upon light irradiation, IS-Micelles exhibited more severe histological damage, less cell proliferation and preferable cell apoptosis as compared to I-Micelles (Supplementary Fig. 31).

In particular, the bioluminescence of 4T1-Luc metastatic nodules in the lungs was simultaneously observed at the end of this experiment. IS-Micelles completely eradicated the metastatic nodules in the lungs owing to potent eradication of primary tumors, while I-Micelles resulted in notable metastatic nodules in the lungs originating from orthotopic 4T1-Luc tumor (Fig. 4f, g). Moreover, the H&E staining was further applied to validate their potent suppression of metastatic nodules in the lungs through IS-Micelles treatment, while a few metastatic nodules were still observed for I-Micelles (Fig. 4f, g). Distinctly, the TRPV1 blockade plays a crucial role in yielding an effective inhibition against distal metastatic nodules arising from their involvement in regulation of EMT pathways (Fig. 2e), although IS-Micelles only exert the local hyperthermia at primary tumor.

Inspired by considerable thermotherapeutic efficacy of IS-Micelles, we further evaluated their antitumor efficacy against orthotopic TNBCs suffering from post-operative failure. Briefly, the surgical resection was applied to ablate the orthotopic 4T1-Luc breast tumors for providing the initial surgical treatment. Then, at 7 days post-surgery, free doxorubicin (DOX), I-Micelles and IS-Micelles at a single dose of 7.5 mg kg^−1^ DOX, 7.5 mg kg^−1^ ICG, or 5.0 mg kg^−1^ SB705498 were administrated via intravenous injection to provide post-operative chemotherapy or thermotherapy against recurrent tumors (Fig. 4h), respectively. After surgical resection, a negligible bioluminescence was observed in all treatment groups except for PBS as a control, demonstrating the successful surgical resection of orthotopic tumors (Fig. 4i), but there were distinct tumor recurrences at 7 days post-surgery. The chemotherapy from DOX was found to suppress the growth of recurrent tumors as compared to the surgical resection alone, however, the tumor growth remained aggressive. Importantly, the TRPV1 blockade-synergized thermotherapy from IS-Micelles led to the potent suppression of recurrent tumors as compared to the thermotherapy alone from I-Micelles, as evidenced by the decreased bioluminescence signals and tumor weights at the end of the experiment (Fig. 4i, j), further validating distinct synergy between TRPV1 blockade and thermotherapy. The photothermal damages of IS-Micelles against recurrent tumors were also evaluated at 6 h post-chemotherapy or post-irradiation using histological, Ki67 and TUNEL staining. When compared with I-Micelles and DOX, IS-Micelles under light irradiation exhibited more severe tumor cell damage, less cell proliferation, and enhanced tumor cell apoptosis (Supplementary Fig. 32).

We also observed the influence of IS-Micelles on the metastatic lung nodules originating from these recurrent breast tumor models at the end of this experiment (Fig. 4k, l). IS-Micelles distinctly inhibited the metastatic nodules in the lungs, whereas the hyperthermia from I-Micelles alone showed much more obvious lung metastasis. In contrast, the additional chemotherapy from DOX failed to generate any anti-metastasis effect as compared to PBS group (Fig. 4k, l). Also, the H&E staining further validated preferable and potent anti-metastasis effect from IS-Micelles with undetectable metastatic nodules in the lungs as compared to those from additional thermotherapy or chemotherapy (Fig. 4k). These results suggest the effectiveness of TRPV1 blockade-synergized thermotherapy to prevent tumor metastasis.

To further unravel the mechanism of IS-Micelles to prevent tumor metastasis and recurrence upon light exposure, the cell wound scratch assay was applied to evaluate the metastatic ability of tumor cells after different treatments, in which 4T1 tumor cells in PBS group as a control exhibited the smallest distance between two separated cell populations, implying the intrinsic metastatic ability. Upon irradiation, IS-Micelles resulted in the reduced migration of tumor cells as compared to I-Micelles, revealing that both TRPV1 blockade and hyperthermia from IS-Micelles synergistically contribute to distinctly decreased occurrence of cell metastasis (Supplementary Fig. 33), possibly due to the involvement of TRPV1 blockade-synergized thermotherapy in metastasis-relevant EMT pathways (Fig. 2e). Hence, IS-Micelles yield preferable thermotherapeutic potency against primary, metastatic and recurrent tumors via TRPV1 blockade-mediated suppression of HSP70 upregulation and cell migration upon light exposure.

### TRPV1 blockade possesses superior safety via selective suppression of stressfully overexpressed HSP70 without injury on normal tissues

**Fig. 5:**
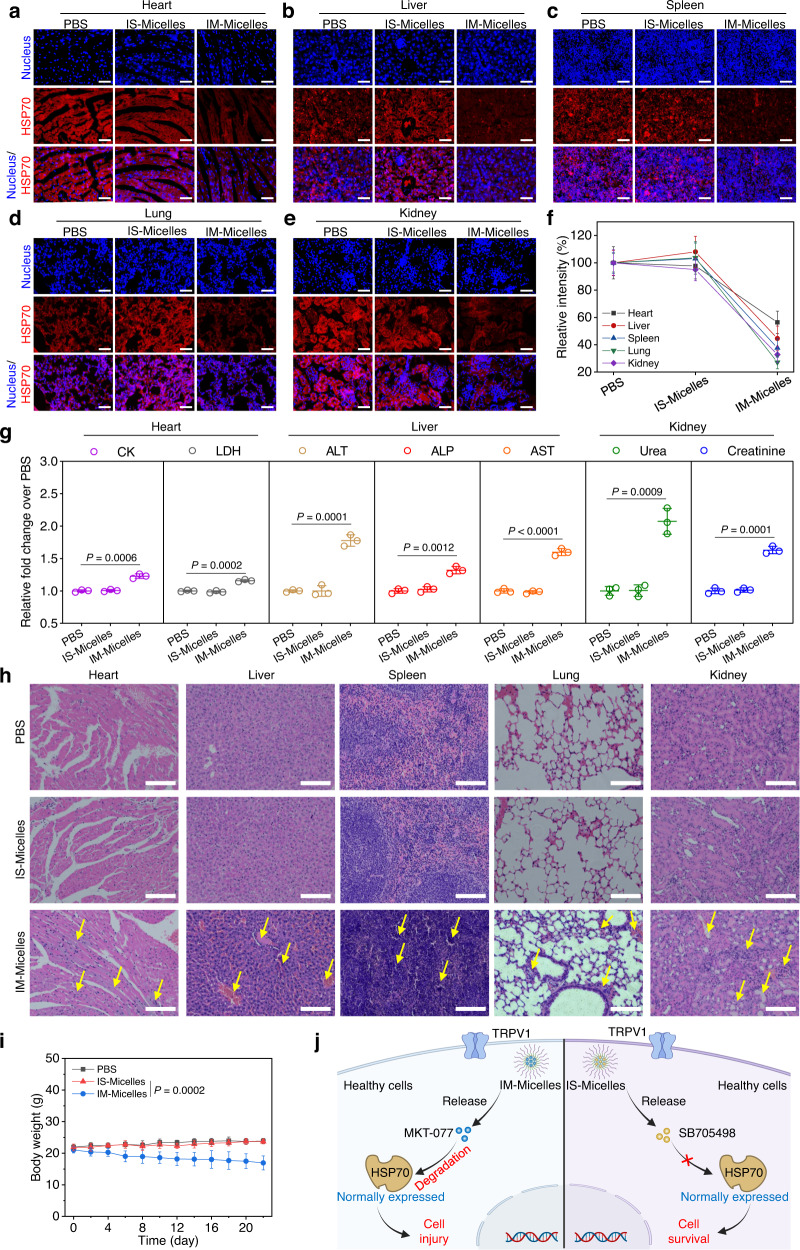
TRPV1 blockade confers superior safety without interfering normally expressed HSP70 in healthy tissues. CLSM images of HSP70 (Alexa Fluor 594-labeled anti-HSP70 antibody, red) in different sections of non-cancerous tissues, including heart (**a**), liver (**b**), spleen (**c**), lung (**d**), kidney (**e**), and corresponding fluorescence intensity analysis (**f**) at 24 h post-injection of IS-Micelles or IM-Micelles at the dose of 5.0 mg kg^−1^ SB705498 or 5.0 mg kg^−1^ MKT-077 (equal to 7.5 mg kg^−1^ ICG) using immunofluorescence staining (*n* = 3 independent experiments). Scale bars, 100 μm. **g** Blood levels of heart function markers (CK and LDH), hepatic function markers (ALP, ALT and AST) and renal function markers (Urea and Creatinine) treated with 5.0 mg kg^−1^ SB705498 (IS-Micelles) or MKT-077 (IM-Micelles) at 3 days post-injection (*n* = 3 biological replicates). **h** H&E images of normal tissues from the mice at 3 days after intravenous injection of 5.0 mg kg^−1^ SB705498 (IS-Micelles) or MKT-077 (IM-Micelles). Yellow arrows represent the histological damage such as fibrosis in different tissues, and renal tubular dilution in kidneys. Scale bars, 200 μm. **i** Body weights of the mice after intravenous injection of 5.0 mg kg^−1^ SB705498 (IS-Micelles) or MKT-077 (IM-Micelles) during 22 days (*n* = 5 mice per group). **j** Schematic illustration of TRPV1 blockade-mediated superior safety without interference on normally expressed HSP70 of healthy cells using IS-Micelles (right), as compared to IM-Micelles (left) which led to inevitable degradation of normally expressed HSP70 with severe concurrent toxicities. Data are presented as mean ± SD (**f**, **g**, **i**). Statistical significance was determined by one-way ANOVA with Tukey’s post hoc test. The experiments for **a**–**e**, and **h** were repeated three times independently with similar results. Source data are provided as a Source Data file.

Normally expressed HSP70 behaves as an important and indispensable molecular-chaperone to ensure accurate protein folding, thus maintaining intracellular homeostasis to defend against external stimuli^1^. To investigate the safety of TRPV1 blockade, the immunofluorescence staining was applied to evaluate the expression of HSP70 in the healthy tissues using anti-HSP70 primary antibody and Alexa Fluor 594 conjugated secondary antibody, and conventional HSP70 inhibitor MKT-077 and ICG co-encapsulated micelles (IM-Micelles) were applied as a control^42^. Briefly, IS-Micelles and IM-Micelles were intravenously injected into the healthy mice at a single dose of 5.0 mg kg^−1^ SB705498 or MKT-077, respectively, followed by CLSM observation of HSP70 expression in different tissues. Apparently, at 24 h post-injection, IM-Micelles caused a notable decrease of HSP70 expression in multiple healthy tissues owing to non-selective inhibition of HSP70 using MKT-077, while IS-Micelles exhibited negligible influence on normally expressed HSP70 in all healthy tissues as compared to the PBS groups, owing to the selective downregulation of stressful HSP70 through TRPV1 blockade-mediated suppression of HSF1 nuclear translocation (Fig. 5a–e), which is also validated by the fluorescence intensity analysis (Fig. 5f). Thus, IS-Micelles might possess superior safety compared to IM-Micelles due to negligible influence on normally expressed HSP70, showing an emerging potential for safe and selective inhibition of stressfully overexpressed HSP70.

To confirm the safety, we further evaluated the influence of IS-Micelles and IM-Micelles on the functions the heart, liver and kidney at 3 days post-injection. As a result, IS-Micelles displayed negligible influence on typical heart function markers (Lactate Dehydrogenase, LDH and Creatine Kinase, CK), hepatic function markers (Alanine Transaminase, ALT, Alkaline Phosphotase, ALP and Aspartate Aminotransferase, AST) and kidney function markers (Urea and Creatinine) as compared to PBS-treated mice, while IM-Micelles as a control caused distinct heart, liver and kidney function markers variation, thus leading to severe toxicity due to inevitable suppression of normally expressed HSP70 in healthy tissues (Fig. 5g), further demonstrating the good safety of TRPV1 blockade-mediated selective suppression of stressful HSP70 inside cancer cells. To further verify the concurrent toxicity caused by HSP70 inhibition, the H&E staining was applied to evaluate the histological damage. Distinctly, severe myocardial fibrosis, liver fibrosis and bleeding, nuclear contraction in spleen, pulmonary fibrosis and severe renal tubular damage such as tubular dilation were observed in IM-Micelles treated mice, while IS-Micelles exhibited negligible influence on histologically morphological change as compared to PBS control, validating the good safety of IS-Micelles in simultaneously improving thermotherapeutic potency and minimizing off-target toxicity (Fig. 5h). Moreover, IM-Micelles administration also resulted in the continuous body weight loss owing to severe multiple-organ histological damage, while IS-Micelles showed negligible influence on body weight variation (Fig. 5i), further confirming the distinct advantages of IS-Micelles for in vivo cancer therapy. Notably, TRPV1 blockade, instead of HSP70 inhibitors, represents a revolutionary strategy to enhance thermotherapeutic efficacy with minimal off-target toxicities, originating from its negligible interference on normally expressed HSP70 in healthy tissues (Fig. 5j).

### TRPV1 blockade-synergized thermotherapy yields distinct therapeutic potency against a variety of malignant tumors

**Fig. 6:**
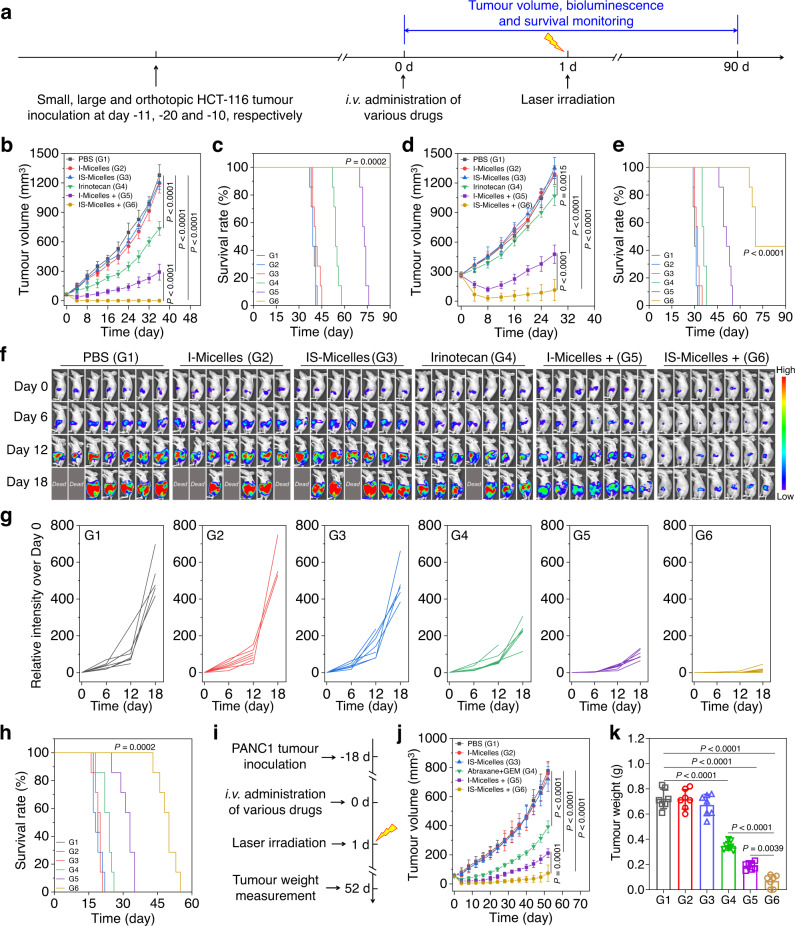
TRPV1 blockade-synergized thermotherapy yields preferable therapeutic potencies against a variety of malignant tumors. **a** Timeline for the treatment of small, large and orthotopic HCT-116 colon tumor models. Tumor growth profiles (**b**) and survival curves (**c**) of the mice bearing small HCT-116 tumor models (50–75 mm^3^) treated with PBS, irinotecan, I-Micelles and IS-Micelles under light irradiation (+) or not (*n* = 7 mice per group). Tumor growth profiles (**d**) and survival curves (**e**) of the mice bearing large HCT-116 tumor model (250–300 mm^3^) treated with PBS, irinotecan, I-Micelles and IS-Micelles under light irradiation (+) or not (*n* = 7 mice per group). Bioluminescence images (**f**), corresponding bioluminescence intensities (**g**) and survival curves (**h**) of the mice bearing orthotopic HCT−116-Luc tumor model treated with PBS, irinotecan, I-Micelles and IS-Micelles under light irradiation (+) or not (*n* = 7 mice per group, color bar, Low represents 1000 a.u. and High represents 50000 a.u.). **i** Timeline for the treatment of subcutaneous PANC1 pancreatic tumor models. Tumor growth profiles (**j**) and tumor weight (**k**) of the mice bearing small PANC1 tumor models (50–75 mm^3^) treated with PBS, Abraxane/gemcitabine (GEM), I-Micelles and IS-Micelles under light irradiation (+) or not (*n* = 7 mice per group). Data are presented as mean ± SD (**b**, **d**, **j**, **k**). For **b**, **d**, **j** and **k**, statistical significance was determined by one-way ANOVA with Tukey’s post hoc test. For **c**, **e** and **h**, statistical significance was calculated by log-rank test. Source data are provided as a Source Data file.

Inspired by preferable antitumor efficacy and safety of IS-Micelles via TRPV1 blockade-synergized thermotherapy in TNBC models, we further evaluated their therapeutic potencies against highly aggressive human tumor models that also highly express TRPV1 channels, including HepG2 liver tumors, MDA-MB-231 breast tumors, HCT-116 colorectal tumors and PNAC1 pancreatic tumors^18^. Firstly, immunofluorescence staining was applied to validate the TRPV1 expression in these malignant tumor cells using anti-TRPV1 primary antibody and Alexa Fluor 594 labeled secondary antibody. Prominent red fluorescence from TRPV1 channels was observed in different cancer cell lines including HepG2, MAD-MB-231, HCT-116 and PANC1 cancer cells (Supplementary Fig. 34), suggesting that TRPV1 channels are abundantly expressed on these cells. In all these types of tumor cells, TRPV1 channels were mainly distributed in cell membrane as indicated by the obvious yellow fluorescence merged from the anti-TRPV1 antibody (red) and cell membrane probe (DiO, green) (Supplementary Fig. 34). Then, IS-Micelles and I-Micelles were intravenously administrated into the mice bearing different tumor models at a single dose of 7.5 mg kg^−1^ ICG, or 5.0 mg kg^−1^ SB705498, followed by light exposure (785 nm, 1.5 W cm^−2^) for 5 min at 24 h post-injection and measurement of tumor volume together with survival analysis in the following 90 days (Fig. 6a and Supplementary Fig. 35a). The clinically used formulations as controls were also intravenously administrated into the mice bearing various tumor models including 5.0 mg kg^−1^ oxaliplatin plus 7.5 mg kg^−1^ DOX, 5.0 mg kg^−1^ Doxil, and 7.5 mg kg^−1^ irinotecan. In HepG2, MDA-MB-231 and HCT-116 tumor models, I-Micelles or IS-Micelles alone displayed negligible influence on tumor growth, being similar to that of PBS in the absence of light exposure, demonstrating non-toxic property of I-Micelles and IS-Micelles. The clinically used formulations delayed the tumor progression and prolonged survival time of mice bearing small HepG2, MDA-MB-231 and HCT-116 tumors owing to chemotherapeutic cytotoxicity (Fig. 6b, c and Supplementary Fig. 35b–e), respectively. Hyperthermia alone from I-Micelles could only provide distinct suppression on tumor growth and slightly extended their survival, but obvious tumor recurrence occurred due to intrinsic thermo-resistance of cancer cells. In contrast, upon light exposure, a single injection of IS-Micelles entirely eradicated these malignant tumors without any recurrence during 90 days post-injection regardless of tumor models, leading to 100% survival rate within monitoring periods (Fig. 6b, c and Supplementary Fig. 35b–e). We then explored the synergistic therapeutic efficiency of IS-Micelles against large HCT-116 tumor model (250–300 mm^3^). Owing to the ability of TRPV1 blockade to selectively suppress stressful HSP70, IS-Micelles under light exposure distinctly delayed the tumor growth of large HCT-116 tumor model, as evidenced by the smallest tumor volume and prolonged survival period (Fig. 6d, e). Moreover, the antitumor efficacy of TRPV1 blockade-synergized thermotherapy against orthotopic HCT-116-Luc tumor model was further investigated (Fig. 6a). Clearly, the mice receiving IS-Micelles under light irradiation showed the lowest bioluminescence signal and prolonged survival time (Fig. 6f–h), validating the preferable antitumor potency. These results confirm the ability of TRPV1 blockade from IS-Micelles to potentiate thermotherapy against large and orthotopic tumor models.

In addition, the thermotherapeutic potency of IS-Micelles against intractable PANC1 pancreatic tumors was also investigated (Fig. 6i). In the absence of light exposure, I-Micelles and IS-Micelles administration displayed negligible influence on tumor growth as compared to PBS-treated mice, further demonstrating the safety of such formulations, whereas the clinically used formulation of Abraxane/gemcitabine (GEM), partially suppressed progression of PANC1 pancreatic tumors but still displayed aggressive tumor growth, as further verified by the tumor weight measurements at the end of experiments (Fig. 6j, k). Importantly, IS-Micelles exhibited more severe inhibition on tumor growth as compared to I-Micelles in the presence of light exposure, but were still compromised by incomplete ablation, possibly owing to their insufficient accumulation and penetration in poorly permeable pancreatic cancer (Fig. 6j, k)^43^. These results show that TRPV1 blockade behaves as an effective approach to synergize thermotherapy using IS-Micelles.

### TRPV1 blockade regulates TGFβ-mediated fibrotic stroma of PDAC tumor model via HSF1 modulation

**Fig. 7:**
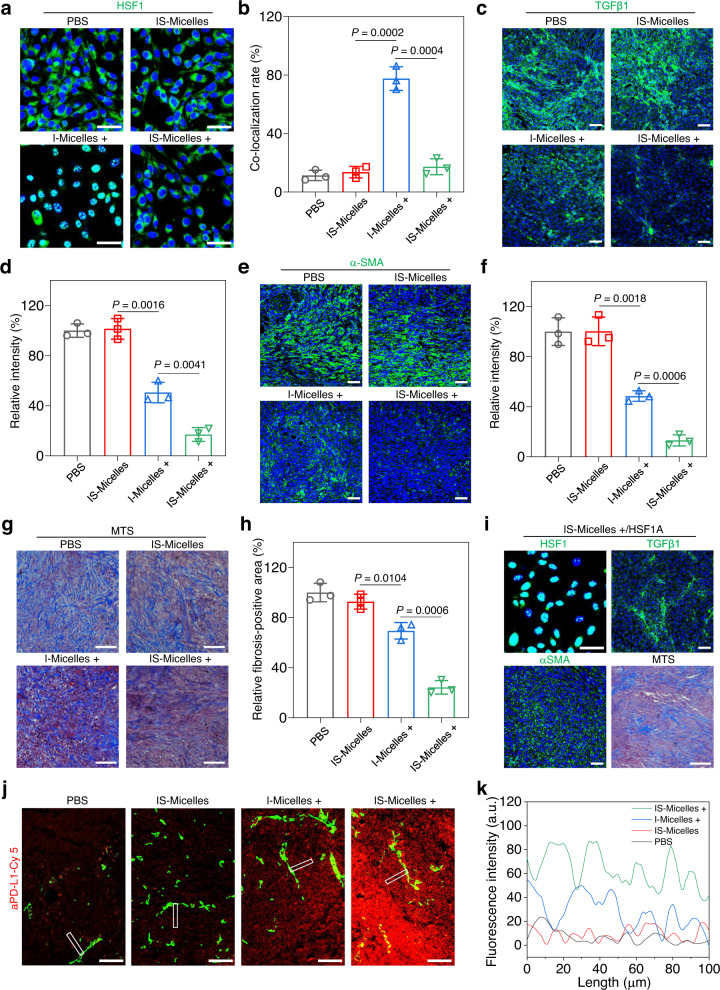
TRPV1 blockade-synergized thermotherapy degrades highly fibrotic tumor stroma of PANC02 tumors and facilitates aPD-L1 infiltration via suppressing HSF1 nuclear translocation-mediated TGFβ1 upregulation. CLSM images of HSF1 (green) (**a**) and corresponding co-localization rate analysis of HSF1 with nucleus (blue) (**b**) in PANC02 tumor cells treated with I-Micelles or IS-Micelles under light irradiation (+) or not (*n* = 3 independent experiments). Scale bars, 50 μm. CLSM images of TGFβ1 (green) (**c**) and corresponding fluorescence intensity analysis (**d**) of PANC02 tumor sections from the mice treated with different formulations (*n* = 3 independent experiments). Scale bars, 50 μm. CLSM images of α-SMA (green) (**e**) and corresponding fluorescence intensity analysis (**f**) of PANC02 tumor sections from the mice treated with different formulations (*n* = 3 independent experiments). Scale bars, 50 μm. Masson’s trichrome staining (MTS) (**g**) and corresponding fibrosis analysis (**h**) of PANC02 tumor sections from the mice treated with different formulations (*n* = 3 independent experiments). Scale bars, 50 μm. **i** CLSM images of HSF1 (green) translocation in PANC02 tumor cells treated with IS-Micelles and HSF1A under light irradiation (+), and CLSM images of TGFβ1 (green), α-SMA (green) and Masson’s trichrome staining (MTS) of PANC02 tumor sections from the mice treated with IS-Micelles and HSF1A under light irradiation (+) (*n* = 3 independent experiments). Scale bars, 50 μm. CLSM images of Cy5 labeled aPD-L1 (red) (**j**) and corresponding fluorescence intensity analysis (**k**) in PANC02 tumor sections at 24 h post-injection of aPD-L1 from the mice treated with different formulations. Scale bars, 100 μm. This experiment was repeated three times independently with similar results. In **c**, **e**, **g**, and **j**, the mice were treated with PBS, IS-Micelles, I-Micelles under light irradiation (+), and IS-Micelles under light irradiation (+), respectively. Data are presented as mean ± SD (**b**, **d**, **f**, **h**). Statistical significance was determined by one-way ANOVA with Tukey’s post hoc test. Source data are provided as a Source Data file.

The compact ECM such as collagen I and fibronectin constitute pathological barriers of intractable tumors such as PDAC models, which often impedes the infiltration of antitumor compounds^44,45^. Interestingly, HSF1 as an important transcriptor is also reported to be associated with TGFβ1 modulation^46^, which plays a vital role in CAFs proliferation, differentiation, and subsequent ECM production^11^. To demonstrate that TRPV1 blockade might enable the manipulation of tumor stroma via suppressing HSF1-mediated TGFβ1 upregulation, we thus firstly investigated the influence of TRPV1 blockade on HSF1 distribution in the PANC02 tumor cells upon hyperthermia using immunofluorescence staining. Clearly, negligible green fluorescence from HSF1 in the nucleus was observed in the cells treated with IS-Micelles under light irradiation, whereas I-Micelles as a control displayed distinct nuclear distribution of HSF1 in the presence of light exposure, as validated by high co-localization of green and blue fluorescence (Fig. 7a, b), suggesting the effectiveness of TRPV1 blockade to suppress HSF1 nuclear translocation upon hyperthermia in PANC02 tumor cells which highly express TRPV1 ion channels (Supplementary Fig. 36). Then, the immunofluorescence staining was performed to investigate the HSP70 expression in PANC02 tumor sections from the mice receiving different treatments. IS-Micelles led to obvious downregulation of red fluorescence from anti-HSP70 antibody as compared to that treated with I-Micelles under light irradiation, confirming the ability of TRPV1 blockade to suppress stressful HSP70 upregulation (Supplementary Fig. 37). Afterwards, the immunofluorescence staining was further utilized to evaluate the in vivo TGFβ1 level of PANC02 tumor section from the mice treated with IS-Micelles at 24 h post-irradiation. Distinctly, IS-Micelles alone possessed negligible influence on TGFβ1 expression as compared to PBS group (Fig. 7c, d), owing to lysosomal entrapment of TRPV1 antagonist. Importantly, upon light exposure, IS-Micelles led to obviously diminished green fluorescence as compared to hyperthermia-treated mice from I-Micelles, reasonably due to considerable cytoplasmic translocation of SB705498 for effective TRPV1 blockade. Apparently, TRPV1 blockade plays an important role in downregulating TGFβ1 expression upon thermotherapy.

To further investigate the influence of TGFβ1 attenuation on CAFs, the levels of α smooth muscle actin (α-SMA) and fibroblasts activation proteins α (FAPα) as two representative markers for quantifying CAFs^47,48^, were evaluated using immunofluorescence staining at 24 h post-irradiation. IS-Micelles only led to a few α-SMA- and FAPα-positive fibroblasts under light irradiation, as indicated by the disappearance of green fluorescence (Fig. 7e, f and Supplementary Fig. 38), while the hyperthermia from I-Micelles displayed obvious green fluorescence from α-SMA and FAPα. It shows that TRPV1 blockade effectively suppresses the CAFs proliferation and differentiation via attenuating TGFβ1 expression upon hyperthermia. Since myofibroblastic CAFs are responsible for highly fibrotic tumor stroma^49^, the immunohistochemical staining of two typical ECM proteins, collagen I and fibronectin, were further applied to evaluate the deposition of ECM proteins in the mice treated with IS-Micelles at 24 h post-irradiation. Consequently, less collagen I and fibronectin were observed in tumor sections from the mice treated with IS-Micelles under light irradiation, as indicated by the obviously diminished brown color when compared with those from the mice receiving IS-Micelles alone and I-Micelles with light exposure (Supplementary Fig. 39). TRPV1 blockade thus plays a key role in preventing extracellular matrix deposition upon hyperthermia. Moreover, the Masson’s trichrome staining (MTS) that is frequently applied to evaluate the fibrosis of tumors^50^, also revealed a notably diminished desmoplasia upon treatment with IS-Micelles under light exposure (Fig. 7g, h). These results verify the ability of TRPV1 blockade to modulate fibrotic stroma of pancreatic tumor via attenuating TGFβ1 levels.

To further demonstrate the correlation between blockade of HSF1 nuclear translocation and TGFβ1 attenuation in pancreatic tumor, HSF1A, a potent HSF1 agonist^51^, was intratumorally injected into PANC02 tumors at 30 min before light irradiation. Clearly, when combined with HSF1A, IS-Micelles treatment caused the re-translocation of HSF1 into nucleus upon light exposure, and resulted in a similar level of TGFβ1 expression to that of I-Micelles with hyperthermia alone (Fig. 7i), thereby causing the reduced suppression of the fibroblasts proliferation and fibrosis amelioration. Thus, TRPV1 blockade-mediated suppression of HSF1 nuclear translocation is a key process in alleviating tumor matrix in PDAC models.

Subsequently, the immunofluorescence staining of vascular endothelial growth factor A (VEGF-A) that is closely associated with vascular permeability^52^, was applied to verify the ability of IS-Micelles-mediated hyperthermia to regulate the vascular permeability in tumor. Both I-Micelles and IS-Micelles led to the distinct increases of VEGF-A expression upon light exposure as compared to PBS or IS-Micelles alone (Supplementary Fig. 40), confirming that the hyperthermia is able to enhance vascular permeability of tumor^53,54^.

Immune checkpoint blockade (e.g. aPD-L1) emerges as an effective strategy to treat cancers^55–58^, but suffers from poor clinical outcomes in treating highly fibrotic and immunosuppressive tumors^59^. We demonstrated the ability of TRPV1 blockade to improve the infiltration of aPD-L1 into PANC02 tumors that were treated with IS-Micelles under light irradiation. Briefly, Cy5-labeled aPD-L1 was intravenously injected into the mice bearing PANC02 tumors at 24 h post-irradiation, followed by the immunofluorescence staining of tumor sections at 24 h post-injection of aPD-L1 (Fig. 7j, k). Clearly, aPD-L1 displayed considerable extravasation and deep penetration of ~100 μm in the tumor of the mice receiving IS-Micelles with light exposure, while only a shallow penetration of aPD-L1 in the tumor was observed in the mice treated with PBS or I-Micelles under light irradiation. Hence, the TRPV1 blockade-mediated TGFβ1 downregulation and hyperthermia-induced increase of vascular permeability cooperatively enhance the infiltration of macromolecular aPD-L1 in poorly permeable PDAC models upon light-triggered thermotherapy.

### TRPV1 blockade potentiates thermo-immunotherapy against small PDAC model through the alleviation of immunosuppression and activation of immune responses

Inspired by the considerable infiltration of aPD-L1 in PANC02 tumor, we further investigated the in vivo synergistic antitumor efficacy of aPD-L1 with IS-Micelles against intractable PANC02 tumor model. Briefly, IS-Micelles and I-Micelles were intravenously injected into the mice bearing PANC02 tumors (50–75 mm^3^) at the dose of 7.5 mg kg^−1^ ICG or 5.0 mg kg^−1^ SB705498, followed by light irradiation for 5 min at 24 h post-injection and subsequent intravenous administration of 3.0 mg kg^−1^ aPD-L1 after another 24 h, as well as measurements of tumor volume and survival rate in the following 90 days (Supplementary Fig. 41a). The combination of 8.0 mg kg^−1^ Abraxane and 35.0 mg kg^−1^ GEM were also administrated as a clinically used control. The IS-Micelles/aPD-L1 combination completely eradicated the tumors without any recurrence under light irradiation, and all of the mice survived during 90 days (Supplementary Fig. 41b, c), while IS-Micelles alone failed to totally ablate the tumors due to its disability to prevent immune evasion under light exposure. Meanwhile, the I-Micelles/aPD-L1 combination treatment under light exposure also led to incomplete tumor ablation, reasonably due to the thermo-resistance and limited infiltration of aPD-L1. In contrast, the Abraxane/GEM combination still resulted in rapid tumor growth, and all the mice died in 55 days post-injection, showing that the clinically standard regimen has a very limited efficacy (Supplementary Fig. 41b, c). IS-Micelles-mediated TRPV1 blockade ensures potent thermo-immunotherapeutic efficacy against intractable PDAC model without any recurrence.

Given that highly immunosuppressive microenvironment of PDAC models usually restrains durable immune response^43,60^, we investigated the ability of the IS-Micelles/aPD-L1 combination to relieve in vivo immunosuppression of PANC02 tumor by monitoring in vivo infiltrations of immunosuppressive myeloid-derived suppressor cells (MDSCs) and tumor-associated macrophages (TAMs) in PANC02 tumors using flow cytometry^61^. As shown in Supplementary Fig. 42a, PANC02 tumor itself possessed a high proportion of MDSCs, and aPD-L1 treatment just caused a slight decrease of MDSCs frequency due to inaccessibility of aPD-L1 into PDAC model. Importantly, the IS-Micelles/aPD-L1 combination caused obvious decrease of MDSCs frequency in PANC02 tumors, which was also preferable to those of IS-Micelles alone or I-Micelles/aPD-L1 combination under light exposure. Moreover, IS-Micelles alone also exhibited a distinct decrease of MDSCs frequency as compared to that of I-Micelles in the presence of light exposure, due to the suppression of immunosuppressive cytokine TGFβ1, which can facilitate the proliferation of immunosuppressive cells^2,11^. Thus, TRPV1 blockade plays an important role in suppressing MDSCs through the aPD-L1 infiltration and TGFβ1 modulation in tumor. Meanwhile, PANC02 tumor was found to harbor a large proportion of CD11b^+^F4/80^+^CD206^+^ TAMs (M2-like character), while the IS-Micelles/aPD-L1 combination led to more efficient repolarization of macrophages from M2-like TAMs to CD11b^+^F4/80^+^CD80^+^ TAMs (M1-like character) in PANC02 tumor upon light irradiation, as compared to IS-Micelles alone or I-Micelles/aPD-L1 combination regardless of light irradiation (Supplementary Fig. 42b–d), manifesting the shift of macrophages from protumor to antitumor phenotype^62^. In contrast, the Abraxane/GEM combination failed to efficiently repolarize macrophages into antitumor phenotype (Supplementary Fig. 42d). Thus, IS-Micelles-mediated TRPV1 blockade effectively relieves in vivo immunosuppression, accounting for synergistic thermo-immunotherapy against PDAC model.

To further explore the immune responses from IS-Micelles-synergized aPD-L1, we firstly evaluated the dendritic cells (DCs) maturation in tumor-draining lymph nodes that is a prerequisite for antigen-specific T lymphocytes priming and amplification^63,64^. The IS-Micelles/aPD-L1 combination resulted in notable DCs maturation (CD11c^+^CD80^+^CD86^+^ DCs) in the lymph nodes of mice upon light exposure, also being preferable to IS-Micelles alone or I-Micelles/aPD-L1 combination (Supplementary Fig. 42e). Then, we further monitored the infiltrated cytotoxic T lymphocytes (CTLs) at tumor using flow cytometry. The IS-Micelles/aPD-L1 combination caused a distinctly improved frequency of CTLs (CD45^+^CD3^+^CD8^+^ T cells) in the tumor upon light exposure (Supplementary Fig. 42f), displaying 1.15-fold and 1.2-fold increases as compared to IS-Micelles alone or I-Micelles/aPD-L1 combination under light irradiation, respectively. Distinctly, the TRPV1 blockade-synergized thermo-immunotherapy induces the priming, amplification and infiltration of CTLs for activating T-cell immunity.

On the other hand, the tumor-infiltrating natural killer (NK) cells were also quantified using flow cytometry analysis. The IS-Micelles/aPD-L1 combination upon light irradiation resulted in the distinct increase of NK cells as compared to IS-Micelles alone regardless of light exposure or I-Micelles/aPD-L1 combination even under light irradiation (Supplementary Fig. 42g), suggesting that IS-Micelles/aPD-L1 combination also arouses innate immune activation, owing to the synergy of TRPV1 blockade-mediated ECM modulation, hyperthermia-mediated thermotherapy, and aPD-L1-mediated immunotherapy. Thus, TRPV1 blockade-synergized thermo-immunotherapy effectively triggers the immune responses through the infiltration of tumor-attacking immune cells in PDAC model.

We further demonstrated whether TRPV1 blockade-synergized thermo-immunotherapy arouses long-term memory effect to prevent tumor recurrence. Briefly, the mice bearing PANC02 tumors were initially treated with the IS-Micelles/aPD-L1 combination in the presence of light irradiation, and then the surviving mice without any recurrence were further inoculated with PANC02 tumor cells at 60 days post-administration, followed by measurement of tumor volume in the following 45 days. As shown in Supplementary Fig. 43a, the IS-Micelles/aPD-L1 combination resulted in the total suppression on tumor growth of the mice, showing a distinct long-term memory effect, whereas the tumor growth of naïve mice remained aggressive. To unravel the mechanism of this long-term memory effect, we evaluated the frequency of memory T cells in the spleens of the mice at 60 days post-administration, which is responsible for resisting tumor recurrence^65^. The IS-Micelles/aPD-L1 combination led to ~2.2-fold increase of CD44^high^CD62L^low^ effector memory T cells under light exposure as compared to that of naïve mice (Supplementary Fig. 43b, c), thereby confirming the long-term immune memory effect from the TRPV1 blockade-synergized thermo-immunotherapy.

### TRPV1 blockade potentiates thermo-immunotherapy against large and orthotopic PDAC models through modulating tumor microenvironment

**Fig. 8:**
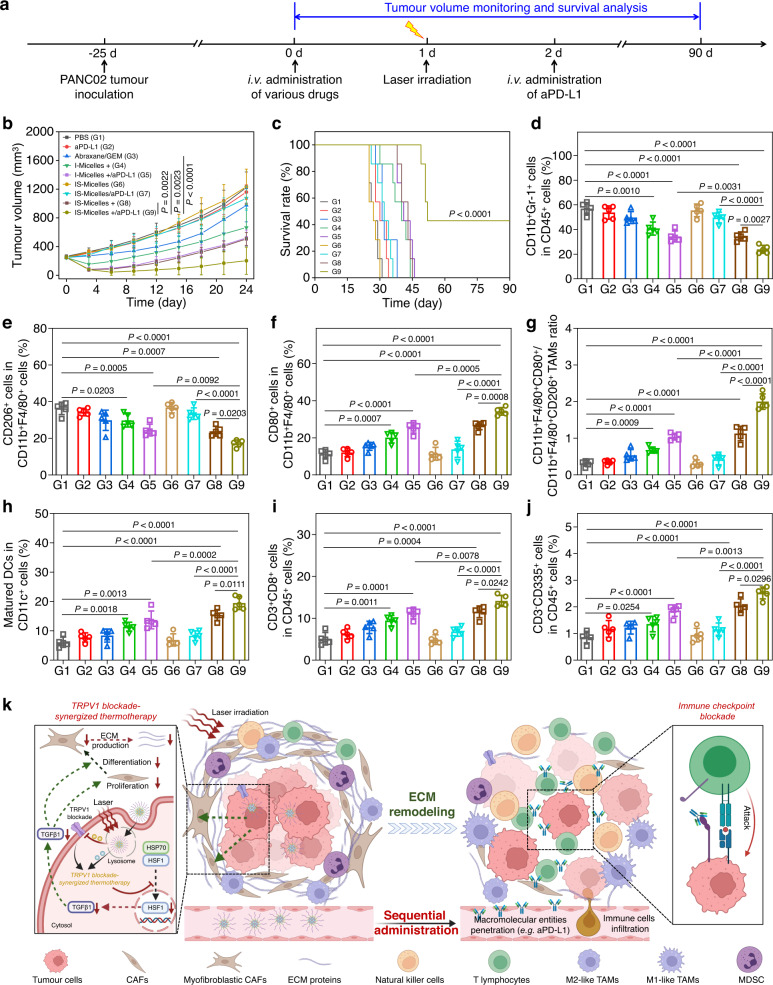
TRPV1 blockade amplifies thermo-immunotherapeutic efficacy and immune response against subcutaneous PANC02 pancreatic tumors using IS-Micelles/aPD-L1 combination. **a** Timeline for the treatment of subcutaneous PANC02 tumor model. Tumor growth profiles (**b**) and survival curves (**c**) of the mice bearing large PANC02 tumor model (250–300 mm^3^) treated with various formulations (*n* = 7 mice per group). Quantification of CD45^+^CD11b^+^Gr−1^+^ MDSCs (**d**), CD11b^+^F4/80^+^CD206^+^ TAMs (**e**) and CD11b^+^F4/80^+^CD80^+^ TAMs (**f**) inside PANC02 tumors at 72 h post-treatment with various formulations (*n* = 5 mice per group). **g** Ratio of CD11b^+^F4/80^+^CD80^+^ TAMs to CD11b^+^F4/80^+^CD206^+^ TAMs inside large PANC02 tumors at 72 h post-treatment with various formulations (*n* = 5 mice per group). **h** Quantification of matured dendritic cells (CD11c^+^CD80^+^CD86^+^ DCs) inside tumor-draining lymph nodes at 72 h post-treatment with various formulations (*n* = 5 mice per group). Quantification of tumor-infiltrating CD45^+^CD3^+^CD8^+^ CTLs (**i**) and CD45^+^CD3^-^CD335^+^ NK cells (**j**) in PANC02 tumors at 72 h post-treatment with various formulations (*n* = 5 mice per group). **k** Schematic illustration of ECM remodeling to facilitate aPD-L1 infiltration for inducing durable immune response via TRPV1 blockade-synergized thermotherapy. Upon TRPV1 blockade, nuclear translocation of HSF1 is effectively blocked which subsequently suppresses TGFβ1 upregulation and secretion, thus leading to inhibition of CAFs proliferation and activation-mediated ECM proteins deposition, ultimately promoting the infiltration of aPD-L1 and immune cells (e.g. NK cells and T lymphocytes) for durable immune response. Dash lines indicate the failure of downstream signals transduction upon blockade of hyperthermia-activated TRPV1 channel. The mice in **b–j** were treated with PBS (G1), aPD-L1 (G2), Abraxane/GEM (G3), I-Micelles under light irradiation (+) (G4), I-Micelles/aPD-L1 under light irradiation (+) (G5), IS-Micelles (G6), IS-Micelles/aPD-L1 (G7), IS-Micelles under light irradiation (+) (G8), and IS-Micelles/aPD-L1 under light irradiation (+) (G9), respectively. Data are presented as mean ± SD (**b**, **d**–**j**). For **b** and **d**–**j**, statistical significance was determined by one-way ANOVA with Tukey’s post hoc test. For **c**, statistical significance was calculated by log-rank test. Source data are provided as a Source Data file.

To further evaluate the synergistic antitumor effect against large PANC02 pancreatic tumor model, which often has highly severe fibrosis and immunosuppression^43^, we established the large PANC02 tumor model with the volume of 250–300 mm^3^. The penetration of IS-Micelles in large PANC02 tumors with less blood vessels and fibrotic stroma was relatively compromised as compared to their penetration in 4T1 tumor models and small PANC02 tumor model (Supplementary Fig. 30)^43,66^, together with moderate distribution in large PANC02 tumor. It suggests that IS-Micelles are even able to penetrate into relatively large and fibrotic tumors, ensuring subsequent in vivo synergy of TRPV1 blockade with thermotherapy. As shown in Fig. 8b, c, the mice receiving clinically standard regimen (Abraxane/GEM combination) exhibited a rapid tumor growth, and all the mice died at 38 days post-treatment. Interestingly, IS-Micelles distinctly delayed the tumor growth upon irradiation via TRPV1 blockade-synergized thermotherapy. More importantly, IS-Micelles/aPD-L1 combination obviously amplified the antitumor efficacy to achieve the tumor eradication in 3 out of 7 mice upon irradiation, suggesting that the selective suppression of stressful HSP70 and modulation of TGFβ pathway cooperatively contribute to the preferable therapeutic potency. Meanwhile, the I-Micelles/aPD-L1 combination as a control showed the reduced antitumor efficacy, reasonably due to the thermo-resistance and insufficient aPD-L1 infiltration resulting from dense ECM (Fig. 8b, c). Then, the flow cytometry was applied to investigate the in vivo immune response of mice bearing large PANC02 tumors receiving different treatments. IS-Micelles under light exposure led to obvious downregulation of immunosuppressive MDSCs and distinct repolarization of M2-like TAMs to M1-like TAMs in large PANC02 tumors owing to effective modulation of TGFβ pathway^11^, and aPD-L1 combination further improved the efficiency to relieve immunosuppression due to the ECM degradation-mediated aPD-L1 infiltration (Fig. 8d–g and Supplementary Fig. 44). Moreover, upon light exposure, the mice receiving IS-Micelles/aPD-L1 combination also displayed distinct maturation of DCs, obvious improvement of tumor-infiltrating CTLs and NK cells, being preferable to other control groups (Fig. 8h–j, and Supplementary Fig. 45). These results demonstrate the ability of TRPV1 blockade-synergized thermo-immunotherapy to elicit robust immune responses, thus accounting for preferable therapeutic efficacy against large pancreatic tumor models.

**Fig. 9:**
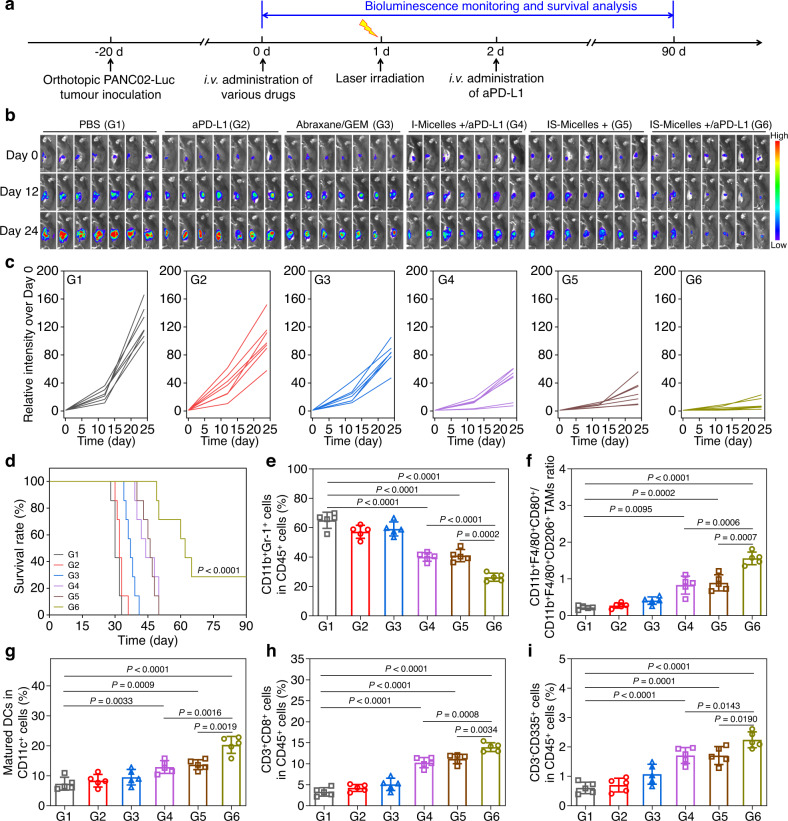
TRPV1 blockade amplifies thermo-immunotherapeutic efficacy and immune response against orthotopic PANC02-Luc pancreatic tumors using IS-Micelles/aPD-L1 combination. **a** Timeline for the treatment of orthotopic PANC02-Luc tumor model. Bioluminescence images (**b**), corresponding bioluminescence intensities (**c**) and survival curves (**d**) of the mice bearing orthotopic PANC02-Luc tumor models treated with different formulations (*n* = 7 mice per group, color bar, Low represents 800 a.u. and High represents 30000 a.u.). Quantification of CD45^+^CD11b^+^Gr−1^+^ MDSCs (**e**) and ratio of CD11b^+^F4/80^+^CD80^+^ TAMs to CD11b^+^F4/80^+^CD206^+^ TAMs (**f**) inside orthotopic PANC02-Luc tumors at 72 h post-treatment with various formulations (*n* = 5 mice per group). **g** Quantification of matured dendritic cells (CD11c^+^CD80^+^CD86^+^ DCs) inside tumor-draining lymph nodes at 72 h post-treatment with various formulations) (*n* = 5 mice per group). Quantification of tumor-infiltrating CD45^+^CD3^+^CD8^+^ CTLs (**h**) and CD45^+^CD3^−^CD335^+^ NK cells (**i**) inside orthotopic PANC02-Luc tumors at 72 h post-treatment with various formulations (*n* = 5 mice per group). The mice in **b**–**i** were treated with PBS (G1), aPD-L1 (G2), Abraxane/GEM (G3), I-Micelles/aPD-L1 under light irradiation (+) (G4), IS-Micelles under light irradiation (+) (G5), and IS-Micelles/aPD-L1 under light irradiation (+) (G6), respectively. Data are presented as mean ± SD (**e–i**). For **d**, statistical significance was calculated by log-rank test. For **e–i**, statistical significance was determined by one-way ANOVA with Tukey’s post hoc test. Source data are provided as a Source Data file.

Inspired by the effective relief of immunosuppression and robust activation of antitumor immune response in subcutaneous PANC02 pancreatic tumor models, we further explored the potency of TRPV1 blockade-synergized thermo-immunotherapy against highly aggressive orthotopic PANC02-Luc pancreatic tumor model (Fig. 9a). As shown in Fig. 9b–d, the mice receiving aPD-L1 treatment showed aggressive tumor growth, while IS-Micelles/aPD-L1 combination effectively suppressed the growth of orthotopic PANC02-Luc tumor under light irradiation, as demonstrated by only a few bioluminescence signals and distinctly extended survival period, being preferable to that of IS-Micelles or I-Micelles/aPD-L1 combination. These results suggest the effectiveness of TRPV1 blockade to potentiate thermo-immunotherapy against orthotopic PDAC models through restraining both HSP70 and TGFβ self-defense pathways. Afterward, the flow cytometry was further applied to investigate the in vivo immune responses of the mice receiving different treatment regimens. As compared to subcutaneous PANC02 tumor models, higher populations of immunosuppressive MDSCs and M2-like TAMs were found in the orthotopic PANC02-Luc tumor model (Supplementary Figs. 44a, b and 46a, b). aPD-L1 alone showed a negligible influence on the frequencies of immunosuppressive MDSCs and M2-like TAMs (Supplementary Fig. 46a, b), while IS-Micelles/aPD-L1 under light exposure resulted in 2.2-fold decrease of immunosuppressive MDSCs and 5.7-fold increase of M1-like TAMs to M2-like TAMs ratio, which was much more efficient than those of mice treated with I-Micelles/aPD-L1 or IS-Micelles under light exposure (Fig. 9e, f and Supplementary Fig. 46). Clearly, TRPV1 blockade-synergized thermo-immunotherapy led to effective relief of severe immunosuppression via TGFβ self-defense pathway modulation and considerable aPD-L1 infiltration. Moreover, aPD-L1 itself displayed a negligible impact on the maturation of DCs, while IS-Micelles/aPD-L1 under light exposure led to 1.6- and 1.5-fold increases of DCs maturation as compared to those of mice receiving I-Micelles/aPD-L1 or IS-Micelles under light exposure, respectively (Fig. 9g and Supplementary Fig. 47a), thus accounting for the priming and subsequent infiltration of CTLs to kill tumor cells (Fig. 9h and Supplementary Fig. 47b). Furthermore, IS-Micelles/aPD-L1 under light exposure also resulted in the highest infiltration of NK cells (Fig. 9i and Supplementary Fig. 47c), confirming the capacity of TRPV1 blockade-synergized thermo-immunotherapy to activate innate immunity. These results further validate the ability of TRPV1 blockade-synergized thermo-immunotherapy to activate robust antitumor immunity via retraining both HSP70 and TGFβ self-defense pathways, ultimately yielding preferable antitumor potency against orthotopic PDACs.

## Discussion

We have developed nanoparticle-mediated TRPV1 blockade as a highly effective approach to modulate nuclear translocation of HSF1, which selectively suppresses HSP and TGFβ self-defense pathways in tumors for achieving potent thermo-immunotherapy against highly malignant tumors such as intractable pancreatic cancers. We constructed TRPV1-KD tumor model to elucidate that TRPV1 blockade is able to inhibit Ca^2+^ influx and subsequent HSF1 nuclear translocation, and thus suppress the stressfully overexpressed HSP70 upon hyperthermia, leading to the enhanced cancer thermotherapy through alleviation of thermo-resistance. To achieve effective synergy of TRPV1 blockade and thermotherapy, polymeric IS-Micelles were explored as nanoparticles-based vehicle to enable tumor-targeted synchronous delivery of TRPV1 antagonist and thermotherapeutic agent for tumor-selective TRPV1 blockade-synergized thermotherapy. These IS-Micelles showed distinct thermotherapeutic efficacies against primary, metastatic and recurrent TNBCs, a variety of TRPV1-positive malignant human tumors including HepG2, MDA-MB-231 and PANC1 tumors, as well as small, large and orthotopic HCT-116 tumor models. Meanwhile, such TRPV1 blockade conferred superior safety over conventional HSP70 inhibitors owing to the capacity to avoid off-target toxicity through the effective identification of stressfully overexpressed HSPs from normally expressed HSPs. In particular, we further recognized that the TRPV1 blockade-mediated inhibition of HSF1 nuclear translocation is able to restrain TGFβ pathway to promote effective decomposition of tumor stroma for improving the infiltrations of antitumor therapeutics (e.g. aPD-L1) and immune cells (e.g. CTLs and NK cells) into tumors (Fig. 8k). As a result, the IS-Micelles/aPD-L1 combination yielded TRPV1 blockade-synergized thermo-immunotherapy to efficiently suppress the tumor progression of highly intractable subcutaneous and orthotopic PDAC tumor models through reinvigorated immune responses and relieved immunosuppression. We believe that TRPV1 blockade represents as an effective approach to dismantle self-defenses in tumors for amplifying thermo-immunotherapy against a variety of highly malignant tumors, and offer an insightful avenue in the ion channel blockade toward potent cancer therapy.

## Methods

This research complies with all relevant ethical regulations approved by Soochow University.

### Materials

Copper chloride (CuCl_2_), human serum albumin (HSA), indocyanine Green (ICG), SB705498, EGTA and HSF1A were obtained from Aladdin Reagent. RPMI 1640 medium, fetal bovine serum (FBS), trypsin EDTA solution and Penicillin-streptomycin solution were purchased from Gibco Life Technologies (California, USA). 3-(4,5-Dimethylthiazol-2-yl)-2,5-diphenyltetrazolium bromide (MTT) was obtained from Sigma-Aldrich. Fluo-8 probe was purchased from Abcam. CalciFluor^TM^ Rhod-4, AM probe was obtained from Santa Cruz. Lyso-tracker Green (LTG), Hoechst 33342, and Annexin V-FITC/PI detection kit were purchased from Beyotime Institute of Biotechnology. Anti-PD-L1 antibody (BioXCell, catalog number BE0101, Clone: 10 F.9G2) was obtained from BioXCell. FITC anti-mouse CD11c (BioLegend, catalog number 117306, clone: N418, dilution: 1:200), PE anti-mouse CD80 (BioLegend, catalog number 104708, clone: 16-10A1, dilution: 1:200), APC anti-mouse CD86 (BioLegend, catalog number 105012, clone: GL-1, dilution: 1:200), FITC anti-mouse CD45 (BioLegend, catalog number 103108, clone: 30-F11, dilution: 1:200), APC anti-mouse/human CD11b (BioLegend, catalog number 101212, clone: M1/70, dilution: 1:200), PE anti-mouse Gr-1 (BioLegend, catalog number 108408, clone: RB6-8C5, dilution: 1:200), FITC anti-mouse/human CD11b (BioLegend, catalog number 101206, clone: M1/70, dilution: 1:200), APC anti-mouse F4/80 (BioLegend, catalog number 123116, clone: BM8, dilution: 1:200), PE anti-mouse CD206 (BioLegend, catalog number 141706, clone: C068C2, dilution: 1:200), APC anti-mouse CD3 (BioLegend, catalog number 100236, clone: 17A2, dilution: 1:200), PE anti-mouse CD8a (BioLegend, catalog number 100708, clone: 53-6.7, dilution: 1:200), PE anti-mouse CD335 (BioLegend, catalog number 137604, clone: 29A1.4, dilution: 1:200), PerCP anti-mouse CD8a (BioLegend, catalog number 100732, clone: 53-6.7, dilution: 1:200), PE anti-mouse CD44 (BioLegend, catalog number 397504, clone: C44Mab-5, dilution: 1:200) and APC anti-mouse CD62L (BioLegend, catalog number 104412, clone: MEL-14, dilution: 1:200) were purchased from Biolegend (San Diego, CA, USA). anti-HSF1 antibody (Abcam, catalog number ab52757, clone: EP1710Y, dilution: 1:1000 for western blot and 1:100 for immunostainings), anti-HSP70 antibody (Abcam, catalog number ab181606, clone: EPR16892, dilution: 1:1000 for western blot and 1:100 for immunostainings), anti-CD31 antibody (Abcam, catalog number ab281583, clone: RM1006, dilution: 1:100), anti-VEGF-A antibody (Abcam, catalog number ab52917, clone: EP1176Y, dilution: 1:100), anti-Histone H3 antibody (Abcam, catalog number ab1791, dilution: 1:1000), anti-Ki67 antibody (Abcam, catalog number ab15580, dilution: 1:100), Alexa 594 labeled goat anti-rabbit IgG H&L secondary antibody (Abcam, catalog number ab150080, dilution: 1:200), Alexa 594 labeled goat anti-mouse IgG H&L secondary antibody (Abcam, catalog number ab150116, dilution: 1:200), Alexa 488 labeled goat anti-rabbit IgG H&L secondary antibody (Abcam, catalog number ab150077, dilution: 1:200), goat anti-rabbit IgG H&L secondary antibody (Abcam, catalog number ab6702, dilution: 1:5000) and goat anti-mouse IgG H&L secondary antibody (Abcam, catalog number ab6708, dilution: 1:5000) were purchased from Abcam. Anti-β-actin antibody (Abclonal, catalog number AC026, clone: ARC5115-01, dilution: 1:5000), anti-GAPDH antibody (Abclonal, catalog number AC033, clone: AMC0062, dilution: 1:5000), anti-TGFβ1 antibody (Abclonal, catalog number A15103, dilution: 1:100), anti-α-SMA antibody (Abclonal, catalog number A2319, clone: ARC1913, dilution: 1:100), anti-FAPα antibody (Abclonal, catalog number A6349, dilution: 1:100), anti-collagen I antibody (Abclonal, catalog number A5786, dilution: 1:100), and anti-fibronectin antibody (Abclonal, catalog number A16678, dilution: 1:100) were purchased from Abclonal. Anti-TRPV1 antibody (Proteintech, catalog number 66983-1-Ig, clone: 1A3C9, dilution: 1:500 for western blot and 1:100 for immunostainings) was obtained from Proteintech. Anti-Cleaved Caspase-3 antibody (CST, catalog number 9664S, dilution: 1:1000) was obtained from CST. HRP labeled goat anti-rabbit IgG H&L secondary antibody (ThermoFisher, catalog number 32460, dilution: 1:3000) was purchased from ThermoFisher.

### Cell lines

A549 (catalog number SCSP-503), HepG2 (catalog number SCSP-510), MDA-MB-231 (catalog number SCSP-5043), HCT-116 (catalog number SCSP-5076), and PANC1 (catalog number SCSP-535) cells were purchased from Cell Bank of Type Culture Collection of the Chinese Academy of Sciences (Shanghai, China). 4T1 tagged with luciferase (catalog number IML-021), HCT-116 tagged with luciferase (catalog number IML-052), PANC02 (catalog number IML-092) and PANC02 tagged with luciferase (catalog number IML-045) cells were obtained from IMMOCELL. To construct A549-TRPV1 KD cells, A549-WT were seeded in plates (1.0 × 10^6^ cells/well) followed by transfection with TRPV1 shRNA (5‘-CCG AGG GAT TCA GTA TTT CCT-3‘) (GeneCopoeia, Rockville, MD) using lipofectamine 2000 as the vector to increase transfection efficiency and further incubation with 1.0 μg mL^−1^ puromycin for picking up A549-TRPV1 KD cells. To construct TRPV1 overexpressed A549-TRPV1 cells, the rTRPV1 plasmid (a kind gift from Prof. Jiuping Ding, Huazhong University of Science and Technology, Wuhan, China) was transiently transfected into A549-WT cells using lipofectamine 2000. These cell lines were cultured in RPMI 1640 Medium supplemented with 1% penicillin-streptomycin and 10% FBS under 5% CO_2_ at 37 °C in a humidified incubator.

### Animals and ethics statement

BALB/c mice (female, 18 ± 2 g, 6–8 weeks), BALB/c nude mice (female, 18 ± 2 g, 6–8 weeks) and C57BL/6J mice (female, 18 ± 2 g, 6–8 weeks) were purchased from Shanghai SLAC Animal Technology Co., Ltd. (Shanghai China). Mice were housed in an animal facility under constant environmental conditions (room temperature, 21 ± 1 °C; relative humidity, 40–70% and a 12 h light-dark cycle). All mice had access to food and water. All animal experiments were carried out following protocols approved by Laboratory Animal Center of Soochow University (No. ECSU-2019000179). In our experiment, the maximum tumor burden was 1500 mm^3^ in mice, which is lower than the maximal tumor burden permitted by Laboratory Animal Center of Soochow University. In some cases, this limit has been exceeded owing to the need to observe the lung-metastatic nodules at 21 days post-injection consistently, and then the mice were immediately euthanized.

### Synthesis

For the preparation of CuS-NCs, 1.0 mL CuCl_2_ solution (200.0 mM) was added into 500.0 mg human serum albumin (HSA) dispersing in 9.0 mL deionized water under vigorous stirring together with adjusting the pH to 12 and further addition of Na_2_S (1.0 M) at the Cu/S ratios of 1:4, followed by reaction at 55 °C for 4 h^25^. Finally, CuS-NCs nanoparticles were purified through centrifugation and ultrafiltration (100 kDa MWCO, 1000 g, 20 min/each) for 5 times and stored in PBS solution (pH 7.4, 10.0 mM). For the preparation of IS-Micelles, 1.5 mg ICG and 1.0 mg SB705498 were dissolved in 0.3 mL DMSO, followed by the addition of 8.0 mg PEG_114_-PCL_60_ dissolved in 0.4 mL DMSO. Then, the mixture was dispersed in 7.0 mL deionized water under vigorous ultrasonication for 10 min and further dialysis against deionized water for 24 h (3.5 kDa MWCO). The obtained solution was preserved in PBS solution (pH 7.4, 10.0 mM). I-Micelles as control were similarly prepared as compared to that of IS-Micelles except for the addition of SB705498.

### Characterization

The morphology was observed by transmission electron microscopy (TEM) (HT7700, Hitachi). The hydrodynamic diameter was measured by dynamic light scattering (DLS, Zetasizer ZS90, Malvern). UV-vis spectrophotometer (UV2600, Shimadzu) was applied to record the UV-vis spectra. Inductively coupled plasma optical emission spectrometer (ICP-OES) (710-ES, VARIAN) was used to determine the copper ion concentration. The reversed-phase high-performance liquid chromatography (HPLC) (Agilent 1100, Agilent) was applied to determine the SB705498 concentration.

### Thermal effect and thermal conversion efficiency

To investigate the thermal effect of CuS-NCs, 0.5 mL CuS-NCs at the concentration of 0.05, 0.1, 0.5 and 1.0 mM were exposed to light for 5 min (785 nm, 1.5 W cm^−2^) together with recording the temperature at the interval of 30 s. As to IS-Micelles, 0.5 mL IS-Micelles at the concentration of 2.0, 5.0, 10.0 and 25.0 μg mL^−1^ ICG were applied for testing the thermal effect. To evaluate the thermal conversion efficiency, IS-Micelles (20.0 μg mL^−1^ ICG) were exposed to light for 5 min (785 nm, 1.5 W cm^−2^) together with recording the temperature at the interval of 30 s before reaching the platform, followed by naturally cooling down to room temperature together with calculation of thermal conversion efficiency^39^.

### In vitro drug release

A dialysis method was applied to evaluate the release behavior of SB705498 from IS-Micelles, and free ICG/SB705498 (IS) was taken as a control. Free IS and IS-Micelles (each 1.0 mL, 100.0 μg mL^−1^ SB705498) were respectively added in the various solutions including the pH 5.0 and pH 7.4 buffers. Then, the in vitro drug release was performed in Air Contrast Temperature Oscillator Shaker at 37 °C. Each sample was taken from the release medium at 1, 2, 4, 8, 12 and 24 h with the addition of fresh medium. HPLC (Agilent 1100, Agilent) was used to measure the SB705498 concentration.

### Cellular uptakes and endocytic pathway

For cellular uptake of CuS-NCs, A549-WT and A549-TRPV1 KD cells were seeded in plates (1.0 × 10^6^ cells/well) followed by the addition of CuS-NCs (0.1 mM Cu) and further incubation for 6, 12 and 24 h, respectively. Then, the cells were collected for cell counting and disruption under ultrasonication, and ICP-OES was used to determine the Cu amount. As to the cellular uptake of IS-Micelles in 4T1-Luc breast cancer cells, IS-Micelles and I-Micelles (10.0 μg mL^−1^ ICG) were incubated with 4T1-Luc cells for 6, 12 and 24 h, respectively. Then, the amount of ICG was determined using the fluorescence spectrophotometer (Fluoromax-4, HORIBA) after extraction of ICG from the disrupted 4T1-Luc cells. For the endocytic pathway, the inhibitors including 10.0 µg·mL^−1^ chlorpromazine, 100.0 µg·mL^−1^ amiloride, 5.0 µg·mL^−1^ nystatin were added into 4T1-Luc tumor cells (1.0 × 10^6^ cells/well) followed by 1 h incubation at 37 °C or 4 °C in serum-free RPMI 1640 medium^39^. Then, IS-Micelles (10.0 μg mL^−1^ ICG) were added into the medium for 2 h incubation. Afterward, the cells were collected through trypsin treatment, centrifugation, and lysis under ultrasonication. Finally, the amount of ICG was determined by the fluorescence spectrophotometer (Fluoromax-4, HORIBA).

### In vitro cytotoxicity

To evaluate the thermo-cytotoxicity, A549-WT cells, A549-TRPV1 KD cells and A549-TRPV1 cells (1.0 × 10^4^ cells/well) were incubated with CuS-NCs at the concentration of 0.02, 0.05, 0.1, 0.2 and 0.5 mM Cu for 24 h with or without 20.0 nM SB705498 pre-incubation for 30 min, followed by light irradiation (785 nm, 1.5 W cm^−2^) for 5 min. After another 24 h, the MTT assay was applied to evaluate the cell viability. The viabilities of A549-WT cells, A549-TRPV1 KD and A549-TRPV1 cells that were incubated with various concentrations of CuS-NCs in the presence of SB705498 (20.0 nM) and EGTA (2.0 mM) without irradiation were also assessed via MTT assay to evaluate the dark cytotoxicity. For evaluating the influence of SB705498 on cell viabilities, SB705498 was incubated with A549-WT cells for 30 min at the concentration of 0, 2, 5, 10, 20, 50 and 100 nM. For evaluating the influence of SB705498 on thermo-cytotoxicity, SB705498 was pre-incubated with A549-WT cells for 30 min at 0, 2, 5, 10, 20 and 40 nM in the presence of 0.2 mM CuS-NCs under light irradiation. For evaluating the influence of culturing temperature on cell viabilities, A549-WT cells were cultured under the temperature of 37 °C, 43 °C, 49 °C and 55 °C, respectively, in the presence of SB705498 (20.0 nM) or not. For the cytotoxicity of IS-Micelles, IS-Micelles or I-Micelles were incubated with 4T1-Luc tumor cells for 24 h at the concentration of 1.0, 2.0, 5.0, 10.0, 25.0 μg mL^−1^ ICG, followed by light irradiation for 5 min or not (785 nm, 1.5 W cm^−2^).

### Cell apoptosis

To evaluate the apoptotic behaviors, A549-WT cells (1.0 × 10^6^ cells/well) were incubated with CuS-NCs at the concentration of 0.5 mM Cu for 24 h with or without 20.0 nM SB705498, followed by light irradiation (785 nm, 1.5 W cm^−2^) for 5 min or not. At 24 h post-irradiation, Annexin V-FITC Apoptosis Detection Kit was applied to evaluate the apoptotic behaviors followed by detection with flow cytometry (BD, FACSAria III) and analysis with FlowJo software (Version 10). For the analysis of apoptotic behaviors from IS-Micelles against 4T1-Luc cells upon irradiation, IS-Micelles and I-Micelles were administrated at the concentration of 7.5 µg mL^−1^ ICG following the protocols as mentioned above.

### EdU staining

A549-WT cells were incubated with CuS-NCs (0.2 mM Cu) together with 20.0 nM SB705498 or 2.0 mM EGTA pre-incubation or not, and 4T1-Luc cells were incubated with I-Micelles or IS-Micelles (7.5 μg mL^−1^ ICG) for 24 h, followed by light irradiation (785 nm, 1.5 W cm^−2^) for 5 min. After another 24 h, the cells were stained with EdU assay kit according to the manufacturer’s protocols followed by observation with CLSM (LSM710, Zeiss). Fluorescence intensities were analyzed by Image J software (Version 1.8.0.112).

### Intracellular distribution and disruption of lysosomal membrane

To evaluate the intracellular distribution, IS-Micelles were incubated with 4T1-Luc cells (5.0 × 10^4^ cells/well) seeded in a glass plate for 2 h, followed by washing with PBS and subsequent light irradiation (785 nm, 1.5 W cm^−2^) for 5 min. Then, the 4T1-Luc cells with lysosomes and nucleus respectively stained by Lysotracker Green DND-26 (0.2 mL, 100.0 nM) and Hoechst 33342 (1.0 mL, 1.0 μg mL^−1^) were observed using CLSM (LSM710, Zeiss). Meanwhile, acridine orange (AO) was applied as a sensor to evaluate the acidic organelle integrity. 4T1-Luc cells seeded in a 24-well plate were treated with IS-Micelles (2.0 μg mL^−1^ ICG) for 6 h, followed by light irradiation (785 nm, 1.5 W cm^−2^) for 5 min or not. After another 1 h, the cells were stained with AO (6.0 μM, 1.0 mL) for 15 min followed by rinsing with PBS for three times. Subsequently, CLSM (LSM710, Zeiss) was applied to observe acidic organelle integrity.

### DHE staining

Dihydroethidium (DHE) was an indicator of intracellular reactive oxygen species. 4T1-Luc cells (2.0 × 10^5^ cells/well) were seeded on 24-well plates and incubated with IS-Micelles (2.0 μg mL^−1^ ICG) for 6 h. Then, the cells were washed with PBS for three times and further exposed to light irradiation (785 nm, 1.5 W cm^−2^) for 5 min. Subsequently, 4T1-Luc cells were incubated with DHE (5.0 μM, 1.0 mL) for 30 min at 37 °C. Finally, the cells were observed using CLSM (LSM710, Zeiss).

### Ca^2+^ staining

CalciFluor^TM^ Rhod-4, AM^67^, was applied as a selective red intracellular Ca^2+^ probe for quantifying the amount of Ca^2+^ influx. To investigate the Ca^2+^ influx elicited by hyperthermia, A549-WT cells or A549-TRPV1 KD cells (1.0 × 10^6^ cells/well) were seeded on 24-well plates and incubated with CuS-NCs (0.2 mM Cu) for 24 h in the presence or absence of 20.0 nM SB705498 or 2.0 mM EGTA. Then, the cells were suffered from light irradiation (785 nm, 1.5 W cm^−2^) for 5 min and stained with 1.0 μM CalciFluor^TM^ Rhod-4, AM for 30 min, followed by observation with CLSM (LSM710, Zeiss), or detection with flow cytometry (FACSAria III, BD) and analysis with FlowJo software (Version 10). Fluo-8 (1.0 μM)^68^, was applied as a selective green intracellular Ca^2+^ probe to monitor the calcium influx in 4T1-Luc tumor cells receiving different treatment regimens, followed by observation with CLSM (LSM710, Zeiss).

### Western blot

A549-WT and A549-TRPV1 KD cells were incubated with CuS-NCs (0.2 mM Cu) for 24 h in the presence or absence of 20.0 nM SB705498 or 2.0 mM EGTA, followed by light irradiation (785 nm, 1.5 W cm^−2^) for 5 min. 4T1-Luc cells were treated with I-Micelles or IS-Micelles (7.5 μg mL^−1^ ICG, 5.0 μg mL^−1^ SB705498) for 24 h followed by light irradiation (785 nm, 1.5 W cm^−2^) for 5 min or not. After another 3 h or 24 h, cells were collected to extract total proteins or intranuclear proteins using Nuclear and Cytoplasmic Protein Extraction Kit from Beyotime. The concentration of protein was determined using BCA assay. Then, the proteins (20.0 µg for each sample) were run on the 12% SDS-polyacrylamide gel and transferred to a PVDF membrane, followed by further blocking with 5% bovine serum albumin for 2 h. Afterward, the PVDF membrane was incubated with anti-HSF1 antibody (Abcam, ab52757, dilution: 1:1000), anti-HSP70 antibody (Abcam, ab181606, dilution: 1:1000), anti-Cleaved Caspase-3 antibody (CST, 9664S, dilution: 1:1000), anti-GAPDH antibody (Abclonal, AC033, dilution: 1:5000), anti-β-actin antibody (Abclonal, AC026, dilution: 1:5000), and anti-Histone H3 antibody (Abcam, ab1791, dilution: 1:1000) overnight at 4 °C. Then, the PVDF membrane was incubated with goat anti-rabbit IgG H&L secondary antibody (Abcam, ab6702, dilution: 1:5000) or goat anti-mouse IgG H&L secondary antibody (Abcam, ab6708, dilution: 1:5000) followed by visualization with ECL using a detection system (GE healthcare). For evaluating the TRPV1 expression of A549-TRPV1 KD and A549-TRPV1 cells, anti-TRPV1 antibody (Proteintech, 66983-1-Ig, dilution: 1:500) was applied.

### Immunofluorescence staining of HSF1, HSP70 and TRPV1

A549-WT and A549-TRPV1 KD cells were incubated with CuS-NCs (0.2 mM Cu) for 24 h in the presence or absence of 20.0 nM SB705498 or 2.0 mM EGTA, followed by light irradiation (785 nm, 1.5 W cm^−2^) for 5 min. 4T1-Luc cells were treated with I-Micelles or IS-Micelles (7.5 μg mL^−1^ ICG, 5.0 μg mL^−1^ SB705498) for 24 h in the presence or absence of 10.0 µg mL^−1^ HSF1A followed by light irradiation (785 nm, 1.5 W cm^−2^) for 5 min. After another 3 h, the cells were fixed and permeabilized, and incubated with anti-HSF1 antibody (Abcam, ab52757, dilution: 1:100) or anti-HSP70 antibody (Abcam, ab181606, dilution: 1:100) for 1 h and further labeled with Alexa 488 labeled goat anti-rabbit IgG H&L secondary antibody (Abcam, ab150077, dilution: 1:200) or Alexa 594 labeled goat anti-rabbit IgG H&L secondary antibody (Abcam, ab150080, dilution: 1:200) for 0.5 h. Finally, the cells were stained with Hoechst 33342 (1.0 mL, 1.0 μg mL^−1^) for 10 min followed by observation with CLSM (LSM710, Zeiss). For the evaluation of TRPV1 expression, anti-TRPV1 antibody (Proteintech, 66983-1-Ig, dilution: 1:100) and cell membrane probe DiO were applied to stain different cancer cells. Fluorescence intensities were analyzed by Image J software (Version 1.8.0.112).

### Construction of A549-WT, A549-TRPV1 KD, 4T1-Luc, HepG2, MDA-MB-231, HCT-116, PANC1 and PANC02 tumor models

A549-WT, A549-TRPV1 KD and HepG2, MDA-MB-231, HCT-116 and PANC1 tumor cells (5.0 × 10^6^ cells per mouse) were subcutaneously injected into the BALB/c nude mouse (female, 6–8 weeks) to construct the above subcutaneous tumor models. To establish the orthotopic 4T1-Luc tumor model, 4T1-Luc tumor cells (1.0 × 10^6^ cells per mouse) were injected into the breast pad of BALB/c mouse (female, 6–8 weeks). To establish the orthotopic HCT-116-Luc tumor model, small pieces of HCT-116 tumors (2 mm^3^) was stitched to intestine of BALB/c nude mice (female, 6–8 weeks). To establish the subcutaneous PANC02 tumor model, PANC02 tumor cells (1.0 × 10^6^ cells per mouse) were subcutaneously injected into the C57BL/6J mouse (female, 6–8 weeks). To establish the orthotopic PANC02-Luc tumor model, PANC02-Luc tumor cells (1.0 × 10^6^ cells per mouse) were orthotopically injected into the pancreas of C57BL/6J mouse (female, 6–8 weeks).

### In vivo biodistribution

To evaluate the biodistribution in A549-WT and A549-TRPV1 KD tumors, CuS-NCs (30.0 μmol kg^−1^) were intravenously injected into the BALB/c nude mice (female, 6–8 weeks) bearing A549-WT or A549-TRPV1 KD tumors (*n* = 3 mice per group), and major organs including heart, liver, spleen, lung, kidney and tumor were subsequently picked out at 24 h post-injection and digested with the mixture of aqua regia and perchloric acid at 280 °C. Afterward, ICP-OES (710-ES, VARIAN) was applied to determine the Cu amount in each sample. For the biodistribution of IS-Micelles in 4T1-Luc tumors, I-Micelles and IS-Micelles (7.5 mg kg^−1^ ICG, 5.0 mg kg^−1^ SB705498) were intravenously injected into the BALB/c mice (female, 6–8 weeks) bearing orthotopic 4T1-Luc tumors (*n* = 3 mice per group), followed by extracting ICG from different tissues using methanol and chloroform mixture at 24 h post-injection. Finally, the amount was determined using a fluorescence spectrophotometer (Fluoromax-4, HORIBA).

### In vivo infrared thermography

CuS-NCs (30.0 μmol kg^−1^) were intravenously injected into the BALB/c nude mice (female, 6–8 weeks) inoculated with A549-WT or A549-TRPV1 KD tumors followed by irradiation at 24 h post-injection (*n* = 3 mice per group), the temperature variation at tumor site during 5 min were monitored using an infrared camera (Fotric 225, Fotric). As to IS-Micelles, I-Micelles and IS-Micelles (7.5 mg kg^−1^ ICG, 5.0 mg kg^−1^ SB705498) were intravenously administrated into the BALB/c mouse (female, 6–8 weeks) bearing orthotopic 4T1-Luc tumors (*n* = 3 mice per group), followed by the similar process mentioned above.

### In vivo penetration of IS-Micelles and aPD-L1

IS-Micelles (7.5 mg kg^−1^ ICG, 5.0 mg kg^−1^ SB705498) were intravenously administrated into the mice bearing orthotopic 4T1-Luc tumors or subcutaneous PANC02 tumors with the volume of ~75 mm^3^ or ~250 mm^3^ (*n* = 3 mice per group), and then the tissue sections with 10.0 μm thickness were made in a cryostat followed by fixation in cold acetone and block with 5% BSA for 1 h at room temperature. Afterward, the tumor sections were incubated with anti-CD31 antibody (Abcam, ab281583, dilution: 1:100) and further labeled with Alexa 488 labeled goat anti-rabbit IgG H&L secondary antibody (Abcam, ab150077, dilution: 1:200) for 0.5 h according to the manufacturer’s protocols, followed by observation with CLSM (LSM 710, Zeiss). As to aPD-L1 penetration, Cy5 labeled aPD-L1 was intravenously injected into the mice bearing subcutaneous PANC02 tumors at 24 h post-irradiation (*n* = 3 mice per group).

### In vivo antitumor efficacy

To evaluate the antitumor efficacy, CuS-NCs (30.0 μmol kg^−1^) were intravenously injected into the mice bearing A549-WT or A549-TRPV1 KD tumors and the mice were suffered from irradiation at 24 h post-injection together with intratumoral injection of SB705498 (1.0 mg kg^−1^) 30 min before irradiation or not (*n* = 5 mice per group), followed by measurement of tumor volumes (*V* = 0.5 × *L* × *W*^2^, *V*, *L*, and *W* represent the tumor volume, long diameter, and short diameter, respectively.) and body weights in the subsequent 30 days. For the orthotopic 4T1-Luc tumors, I-Micelles and IS-Micelles (7.5 mg kg^−1^ ICG, 5.0 mg kg^−1^ SB705498) were intravenously injected into the mice bearing orthotopic 4T1-Luc tumors followed by light irradiation at 24 h post-injection, and further observation of bioluminescence and measurement of tumor volume during 21 days (*n* = 5 mice per group). At the end of the experiment, the lungs were picked out to examine the lung metastasis via bioluminescence. To evaluate the antitumor efficacy of IS-Micelles against recurrent tumors, the mice were firstly treated with surgical resection to remove the primary tumor, followed by the monitoring of bioluminescence at tumor sites during 21 days post-irradiation. Meanwhile, at 7 days post-surgery, IS-Micelles (7.5 mg kg^−1^ ICG, 5.0 mg kg^−1^ SB705498), I-Micelles (7.5 mg kg^−1^ ICG) and 7.5 mg kg^−1^ DOX were further applied to provide additional thermotherapy or chemotherapy against recurrent tumors, respectively. Finally, the metastatic nodules in the excised lungs were also observed at the end of experiments.

To investigate the antitumor efficacy of IS-Micelles against HepG2, MDA-MB-231, HCT-116, and PANC1 tumor models, when the tumor volume reaches 50–75 mm^3^ at 14, 15, 11 and 18 days post-inoculation of HepG2, MDA-MB-231, HCT-116 and PANC1 tumor cells, respectively (*n* = 7 mice per group), I-Micelles and IS-Micelles at the doses of 7.5 mg kg^−1^ ICG and 5.0 mg kg^−1^ SB705498 were intravenously injected into the mice bearing the above tumors followed by irradiation at 24 h post-injection, and clinical treatment regimens, including 5.0 mg kg^−1^ oxaliplatin/7.5 mg kg^−1^ DOX for HepG2 tumors, 7.5 mg kg^−1^ irinotecan for HCT-116 tumors, 5.0 mg kg^−1^ Doxil for MDA-MB-231 tumors and 8.0 mg kg^−1^ Abraxane/35.0 mg kg^−1^ GEM for PANC1 tumors were used as the controls. Afterward, tumor volume and survival analysis were observed in the subsequent 90 days. To investigate the antitumor efficacy of IS-Micelles against large and orthotopic HCT-116 tumor models, I-Micelles (7.5 mg kg^−1^ ICG) and IS-Micelles (7.5 mg kg^−1^ ICG, 5.0 mg kg^−1^ SB705498), and 7.5 mg kg^−1^ irinotecan were administrated at 20 days and 10 days post-inoculation of HCT-116 cancer cells with the tumor volume of 250–300 mm^3^ or small pieces of HCT-116-Luc tumors (*n* = 7 mice per group), and bioluminescence of mice were obtained by IVIS Spectrum Lumina III (Perkin Elmer) and further analyzed using living image software (Version 4.5). When the tumor volumes reach 1500 mm^3^, the mice were euthanized to calculate the survival rate^69–71^.

To evaluate the synergistic antitumor efficacy of IS-Micelles and aPD-L1 against subcutaneous PDAC tumor models, when the tumor volume reaches 50–75 mm^3^ or 250–300 mm^3^ (*n* = 7 mice per group), I-Micelles (7.5 mg kg^−1^ ICG) and IS-Micelles (7.5 mg kg^−1^ ICG, 5.0 mg kg^−1^ SB705498) were administrated intravenously into the mice bearing subcutaneous PANC02 tumor models, followed by irradiation at 24 h post-injection, and clinical treatment regimen 8.0 mg kg^−1^ Abraxane/35.0 mg kg^−1^ GEM was taken as a control. Afterward, 3.0 mg kg^−1^ aPD-L1 were intravenously injected at 24 h post-irradiation followed by measurement of tumor volumes (*V* = 0.5 × *L* × *W*^2^, *V*, *L*, and *W* represent the tumor volume, long diameter, and short diameter, respectively.) in the subsequent 90 days. As to the analysis of survival rate, when the tumor volume reaches ~1500 mm^3^, the mice were euthanized to calculate the survival rate. For the orthotopic PANC02-Luc tumor model, different formulations were administrated at 20 days post-inoculation of PANC02-Luc tumor cells followed by monitoring of bioluminescence intensity using IVIS Spectrum Lumina III (Perkin Elmer) and further analysis using living image software (Version 4.5), together with survival analysis.

### Antitumor immune response

Briefly, I-Micelles (7.5 mg kg^−1^ ICG) and IS-Micelles (7.5 mg kg^−1^ ICG, 5.0 mg kg^−1^ SB705498) were administrated intravenously into mice bearing the small PANC02 tumor model (*n* = 3 mice per group), large PANC02 tumor model (*n* = 5 mice per group) and orthotopic PANC02 tumor model (*n* = 5 mice per group), followed by irradiation at 24 h post-injection, and clinical treatment regimen 8.0 mg kg^−1^ Abraxane/35.0 mg kg^−1^ GEM was taken as a control. Afterward, 3.0 mg kg^−1^ aPD-L1 were intravenously injected at 24 h post-irradiation. To evaluate in vivo antitumor immune response, lymph nodes and tumors were harvested at 3 days post-treatments from C57BL/6J mice (female, 6–8 weeks) bearing subcutaneous and orthotopic PANC02 tumors followed by homogenization or digestion using enzymes in staining buffers to prepare single cell suspensions. For T-cell and NK cells analysis, tumor cells were stained with FITC anti-mouse CD45 (BioLegend, 103108, dilution: 1:200), APC anti-mouse CD3 (BioLegend, 100236, dilution: 1:200), PE anti-mouse CD8a (BioLegend, 100708, dilution: 1:200), and PE anti-mouse CD335 (BioLegend, 137604, dilution: 1:200) antibodies for 60 min followed by analysis of CD45^+^CD3^+^CD8^+^CTLs and CD45^+^CD3^−^CD335^+^NK cells. For DC analysis, lymph nodes were stained with FITC anti-mouse CD11c (BioLegend, 117306, dilution: 1:200), PE anti-mouse CD80 (BioLegend, 104708, dilution: 1:200) and APC anti-mouse CD86 (BioLegend, 105012, dilution: 1:200) antibodies for 60 min followed by analysis of CD11c^+^CD80^+^CD86^+^DCs. For effector memory T-cell analysis, the splenocytes of three surviving mice after treatment with IS-Micelles plus aPD-L1 in the presence of light irradiation at 60 d post-irradiation were stained with FITC anti-mouse CD45 (BioLegend, 103108, dilution: 1:200), PerCP anti-mouse CD8a (BioLegend, 100732, dilution: 1:200), PE anti-mouse CD44 (BioLegend 397504, dilution: 1:200) and APC anti-mouse CD62L antibodies (BioLegend, 104412, dilution: 1:200) for 60 min followed by analysis of CD45^+^CD8a^+^CD44^+^CD62L^−^ effector memory T-cells. For immunosuppressive TAMs and MDSCs analysis, tumor cells were stained with FITC anti-mouse/human CD11b (BioLegend, 101206, dilution: 1:200), APC anti-mouse F4/80 (BioLegend, 123116, dilution: 1:200), and PE anti-mouse CD206 (BioLegend, 141706, dilution: 1:200), PE anti-mouse CD80 (BioLegend, 104708, dilution: 1:200) antibodies for 60 min followed by analysis of CD11b^+^F4/80^+^CD80^+^TAMs and CD11b^+^F4/80^+^CD206^+^TAMs, and FITC anti-mouse CD45 (BioLegend, 103108, dilution: 1:200), APC anti-mouse/human CD11b (BioLegend, 101212, dilution: 1:200), and PE anti-mouse Gr-1 (BioLegend, 108408, dilution: 1:200) antibodies for 60 min followed by analysis of CD45^+^CD11b^+^Gr-1^+^MDSCs. These immune cells data were detected with flow cytometry (FACSAria III, BD) and analyzed with FlowJo software (Version 10).

### Transcriptome analysis of CuS-NCs with SB705498

A549-WT were incubated with CuS-NCs at the concentration of 0.2 mM Cu for 24 h in the presence of 20.0 nM SB705498 or not, followed by irradiation to cause hyperthermia. After another 24 h, total RNA was extracted with TRIZOL agents and transcriptome analysis were conducted following the standard operations by the Suzhou Institute of Systems Medicine using the NovaSeq 6000 (Illumina) and further analyzed with IPA software (Basic Version) and STRING software (Version 11.5).

### Serum biochemistry assay and body weight measurement

To investigate potential toxicity, IS-Micelles and IM-Micelles were intravenously injected into the healthy BALB/c mice (female, 6–8 weeks) at a single dose of 5.0 mg kg^−1^ SB705498 or MKT-077 followed by collecting the blood at 3 days post-injection for serum biochemistry detection (*n* = 3 mice per group), including typical heart function markers (CK and LDH), heart function markers (ALT, AST and ALP) and kidney function markers (Urea and Creatinine). The body weights of mice were recorded within 22 days post-injection of IS-Micelles and IM-Micelles (*n* = 5 mice per group).

### Ex vivo histological, Ki67, TUNEL, HSP70, VEGF-A, TGFβ1, α-SMA, FAPα, collagen I, fibronectin and Masson’s trichrome staining

CuS-NCs (30.0 μmol kg^−1^) were injected into the mice bearing A549-WT or A549-TRPV1 KD tumors (*n* = 3 mice per group), followed by irradiation at 24 h post-injection together with intratumoral injection of SB705498 (1.0 mg kg^−1^) or not, followed by irradiation at 24 h post-injection. IS-Micelles (7.5 mg kg^−1^ ICG, 5.0 mg kg^−1^ SB705498) and IM-Micelles (7.5 mg kg^−1^ ICG, 5.0 mg kg^−1^ MKT-077) were injected into the healthy mice (*n* = 3 mice per group). IS-Micelles (7.5 mg kg^−1^ ICG, 5.0 mg kg^−1^ SB705498), I-Micelles (7.5 mg kg^−1^ ICG) and 8.0 mg kg^−1^ Abraxane/35.0 mg kg^−1^ GEM were injected into the mice bearing orthotopic 4T1-Luc tumors or subcutaneous PANC02 tumor models, together with intratumoral injection of 10.0 mg kg^−1^ HSF1A or not, respectively (*n* = 3 mice per group), followed by irradiation at 24 h post-injection. Then, the tissue sections with 10.0 μm thickness were made in a cryostat at 6 h post-irradiation or 24 h post-irradiation or 72 h post-injection, followed by the hematoxylin and eosin (H&E) staining, Ki67 staining (Abcam, ab15580, dilution: 1:100) TUNEL assay (green or red), Masson’s trichrome staining, TGFβ1 staining (Abclonal, A15103, dilution: 1:100), α-SMA staining (Abclonal, A2319, dilution: 1:100), FAPα staining (Abclonal, A6349, dilution: 1:100), collagen I staining (Abclonal, A5786, dilution: 1:100), fibronectin staining (Abclonal, A16678, dilution: 1:100), VEGF-A staining (Abcam, ab52917, dilution: 1:100), and HSP70 staining (Abcam, ab181606, dilution: 1:100) and further labeled with Alexa 594 labeled goat anti-rabbit IgG H&L secondary antibody (Abcam, ab150080, dilution: 1:200) or Alexa 488 labeled goat anti-rabbit IgG H&L secondary antibody (Abcam, ab150077, dilution: 1:200) for 0.5 h in different tissues according to the manufacturer’s protocols. Finally, these tissue sections were observed using bright field microscopy (IX73, Olympus) or CLSM (LSM710, Zeiss). Fluorescence intensities were analyzed by Image J software (Version 1.8.0.112).

### Statistics and reproducibility

Statistical analysis was performed using Graphpdad Prism (Version 8.0.2). All in vitro and in vivo experiments were repeated at least three times and data were presented as mean ± SD. Group size was determined on the basis of the preliminary experiments results, and no statistical method was used to predetermine sample size. The indicated sample size (*n*) represents biological replicates. For all studies, samples were randomly divided into different experimental groups. Group allocation and outcome assessment were not performed in a blinded manner. No data were excluded from the analyses. The statistical significance between two groups was calculated by unpaired two-tailed Student’s *t* test. The statistical significance between multiple groups was calculated by one-way ANOVA with Tukey’s post hoc test. Survival was measured using the Kaplan-Meier method and statistical significance was calculated by log-rank test. *P* < 0.05 was considered statistically significant.

### Reporting summary

Further information on research design is available in the Nature Portfolio Reporting Summary linked to this article.

## Supplementary information

Supplementary Information file

Reporting Summary

## Data Availability

The data that support the findings of this study are available within the paper and its Supplementary Information files. The RNA-sequencing data generated in this study have been deposited in the Gene Expression Omnibus under the accession number GSE199299. Source data are provided with this paper.

## Source data

Source Data
