# Supplementary material for: Nanoparticle-mediated TRPV1 channel blockade amplifies cancer thermo-immunotherapy via heat shock factor 1 modulation
Source: Nat Commun. 2023 Apr 29;14:2498. doi: 10.1038/s41467-023-38128-x (PMC10148815; doi:10.1038/s41467-023-38128-x)
Supplement: Supplementary file 1 — Supplementary Information file [file 41467_2023_38128_MOESM1_ESM.pdf]

Supplementary Information

Nanoparticle-mediated TRPV1 channel blockade amplifies cancer thermo-immunotherapy via heat shock factor 1 modulation

Ting Li[1,#], Shuhui Jiang[1,#], Ying Zhang[1], Jie Luo[1], Ming Li[1], Hengte Ke[1], Yibin Deng[1], Tao Yang[1,2,*], Xiaohui Sun[1,*], and Huabing Chen[1,2,*]

[1]Jiangsu Key Laboratory of Neuropsychiatric Diseases, and College of Pharmaceutical Sciences, Soochow University, Suzhou 215123, China
[2]State Key Laboratory of Radiation Medicine and Protection, Soochow University, Suzhou 215123, China

Keywords: transient receptor potential vanilloid member 1 (TRPV1) blockade; self-defense pathway; thermo-immunotherapy; nanomedicine; cancer therapy

[#]These authors contributed equally to this work.

*Correspondence and requests for materials should be addressed to T.Y. (tyang0920@suda.edu.cn), X.S. (sunxiaohui@suda.edu.cn) and H.C. (chenhb@suda.edu.cn).

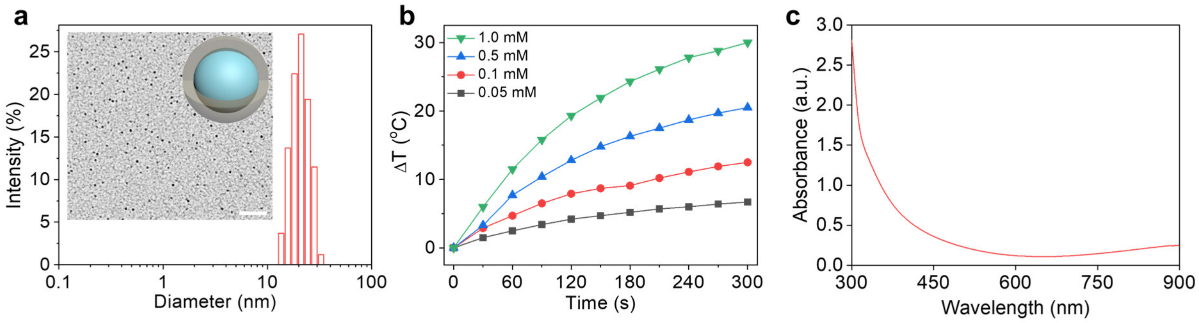

Supplementary Fig. 1. **Characterization of CuS-NCs. a** Size distribution of CuS-NCs using dynamic light scattering with a hydrodynamic diameter of 25.8 ± 4.3 nm. Inset is TEM image with the core size of 7.8 nm and structure of CuS-NCs, Scale bar, 100 nm. **b** Temperature elevation profiles of CuS-NCs at various concentrations of Cu under 5 min light exposure (785 nm, 1.5 W cm^{-2}). **c** UV absorbance of CuS-NCs in near-infrared region. These experiments were repeated three times independently with similar results.

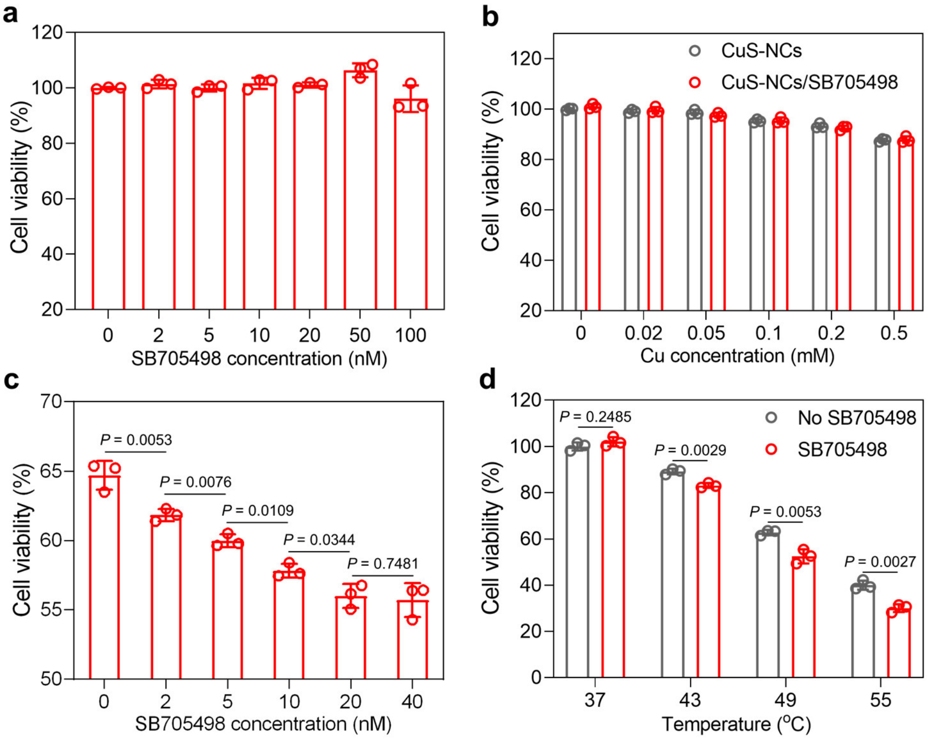

Supplementary Fig. 2. **Cytotoxicity assessment.** Cell viabilities of A549-WT cells incubated with different concentrations of SB705498 for 0.5 h (**a**) or different concentrations of CuS-NCs for 24 h in the presence or absence of SB705498 (20.0 nM) (**b**) (n = 3 biological replicates). **c** Cell viabilities of CuS-NCs against A549-WT cells treated with SB705498 at various doses (0-40 nM) under light irradiation (n = 3 biological replicates). **d** Cell viabilities of A549-WT cells at different culture temperatures in the absence or presence of SB705498 (20.0 nM) (n = 3 biological replicates). Data are presented as mean ± SD. Statistical significance was determined by one-way ANOVA with Tukey's post hoc test.

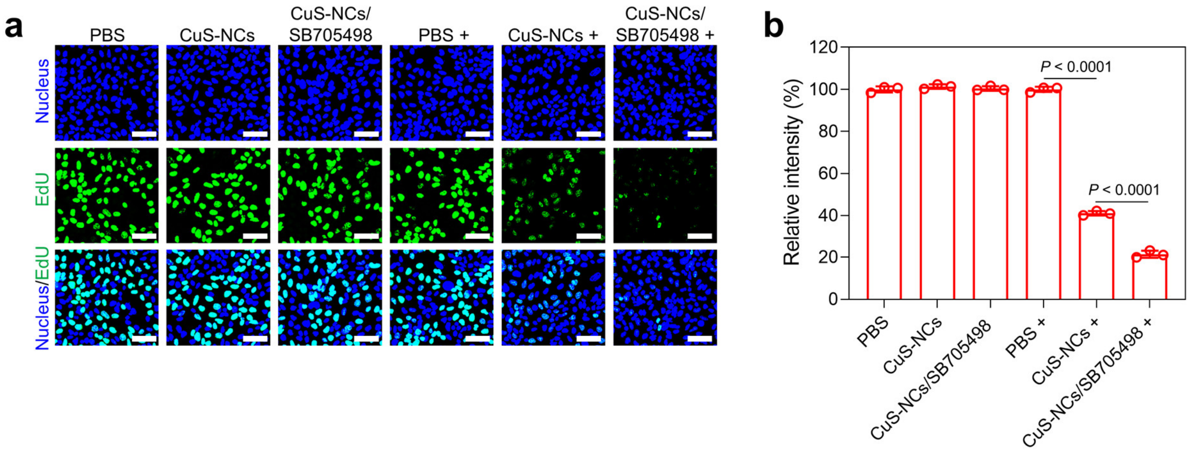

Supplementary Fig. 3. **Cell proliferation assay.** EdU staining (**a**) and relative green fluorescence intensity (**b**) of A549-WT cells treated with or without the hyperthermia from CuS-NCs in the presence or absence of SB705498 (20.0 nM) (n = 3 independent experiments). Scale bars, 50 μm. Data are presented as mean ± SD. Statistical significance was determined by one-way ANOVA with Tukey's post hoc test.

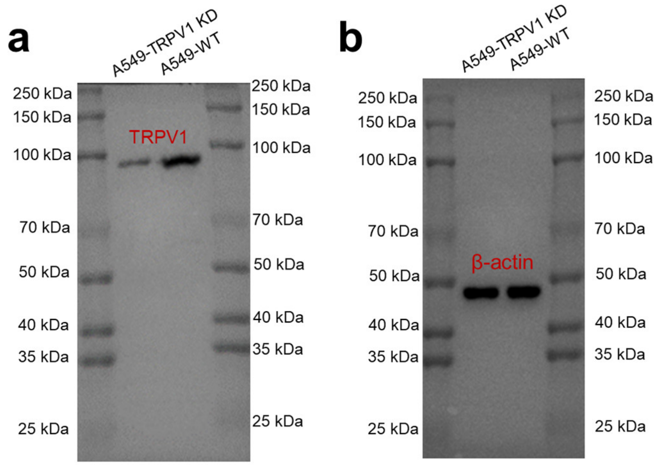

Supplementary Fig. 4. Original whole western blot images of TRPV1 (**a**) and β-actin (**b**) from A549-TRPV1 KD and A549-WT cells with anti-TRPV1 antibody and anti-β-actin antibody. This experiment was repeated three times independently with similar results.

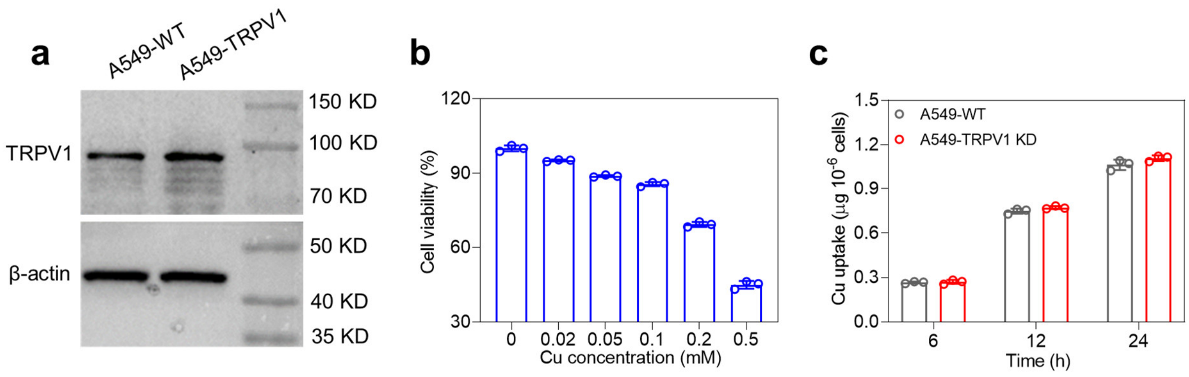

Supplementary Fig. 5. **Effect of TRPV1 expression on thermo-cytotoxicity and cellular uptake of CuS-NCs. a** Western blot images of TRPV1 and β-actin from A549-WT and A549-TRPV1 cells. This experiment was repeated three times independently with similar results. **b** Thermo-cytotoxicity of the hyperthermia from CuS-NCs against A549-TRPV1 cells (n = 3 biological replicates). **c** Cellular uptakes of CuS-NCs by A549-WT cells and A549-TRPV1 KD cells at 6, 12 and 24 h, respectively (n = 3 biological replicates). Data are presented as mean ± SD (**b, c**).

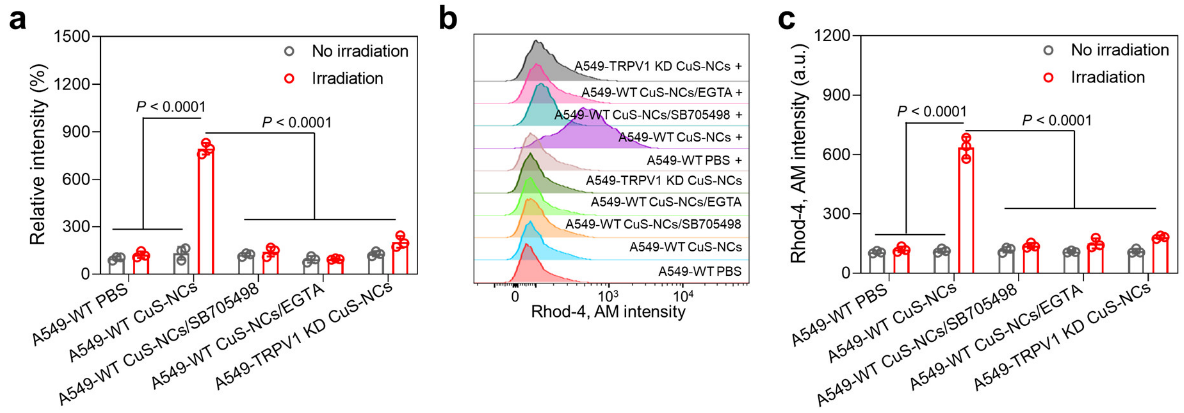

Supplementary Fig. 6. Ca^{2+} influx assessment. a Quantitative analysis of red fluorescence intensities from CalciFluor™ Rhod-4, AM (Rhod-4, AM) probe in A549-WT cells or A549-TRPV1 KD cells treated with or without the hyperthermia from CuS-NCs in the presence or absence of SB705498 (20.0 nM) or EGTA (2.0 mM) (n = 3 biological replicates). **b, c** Representative histogram from flow cytometry (**b**) and corresponding fluorescence intensities (**c**) of intracellular Ca^{2+} in A549-WT cells or A549-TRPV1 KD cells treated with or without the hyperthermia from CuS-NCs in the presence or absence of SB705498 (20.0 nM) or EGTA (2.0 mM) using Rhod-4, AM probe (n = 3 biological replicates). Data are presented as mean ± SD (**a, c**). Statistical significance was determined by one-way ANOVA with Tukey's post hoc test.

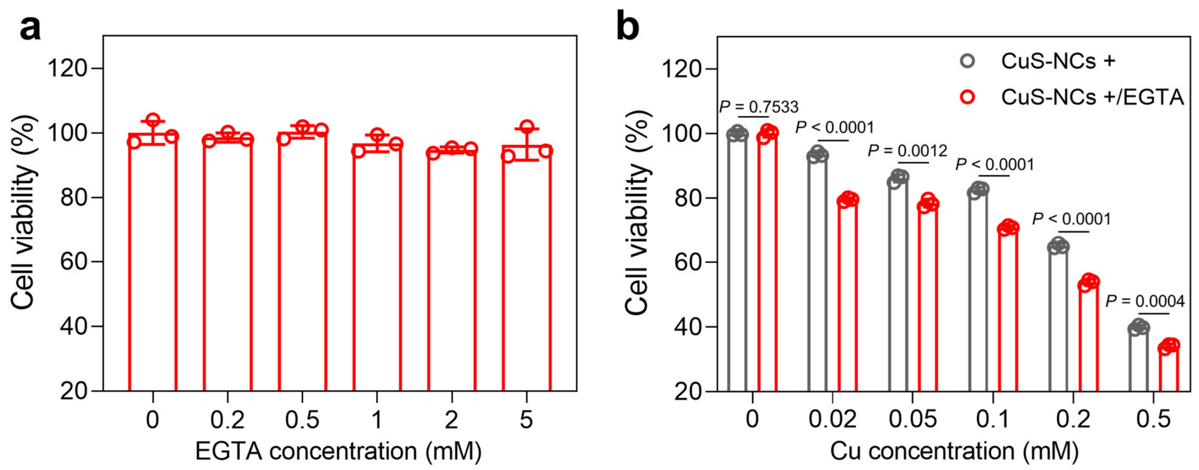

Supplementary Fig. 7. Effect of Ca^{2+} scavenging on the CuS-NCs induced thermo-cytotoxicity. a Cell viabilities of A549-WT cells after treatment with various concentrations of EGTA for 0.5 h. EGTA possessed negligible cytotoxicity (n = 3 biological replicates). **b** Cell viabilities of A549-WT cells after incubation with different concentrations of CuS-NCs for 24 h in the presence or absence of EGTA (2.0 mM) pre-incubation for 0.5 h upon irradiation (n = 3 biological replicates). Data are presented as mean ± SD (**a, b**). Statistical significance was determined by one-way ANOVA with Tukey's post hoc test.

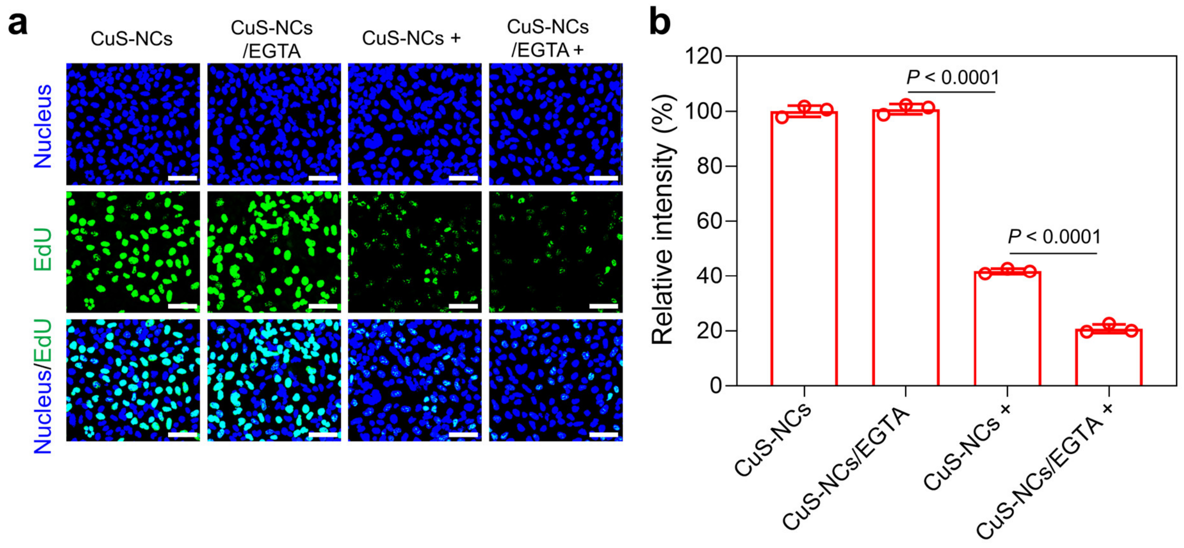

Supplementary Fig. 8. **Cell proliferation assay.** EdU staining (**a**) and relative green fluorescence intensity (**b**) of A549-WT cells treated with or without the hyperthermia from CuS-NCs in the presence or absence of EGTA (2.0 mM) pre-incubation for 0.5 h. EdU conjugated with Azide 488 was applied as a detector to evaluate cell proliferation, representing cells during S phase (green). CuS-NCs with EGTA showed distinct inhibition on cell proliferation as compared to CuS-NCs under light exposure (n = 3 independent experiments). Scale bars, 50 µm. Data are presented as mean ± SD. Statistical significance was determined by one-way ANOVA with Tukey's post hoc test.

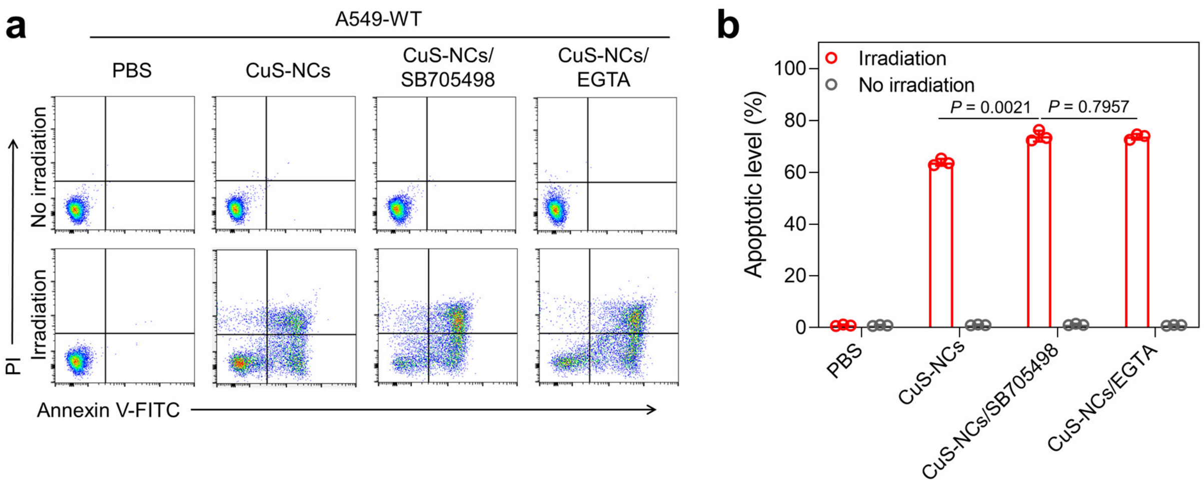

Supplementary Fig. 9. **Cell apoptosis assay.** Representative flow cytometry dot plots (**a**) and corresponding apoptotic levels (**b**) in A549-WT cells treated with or without the hyperthermia from CuS-NCs in the presence or absence of SB705498 (20.0 nM) or EGTA (2.0 mM) using Annexin V/PI staining (n = 3 biological replicates). Data are presented as mean ± SD. Statistical significance was determined by one-way ANOVA with Tukey's post hoc test.

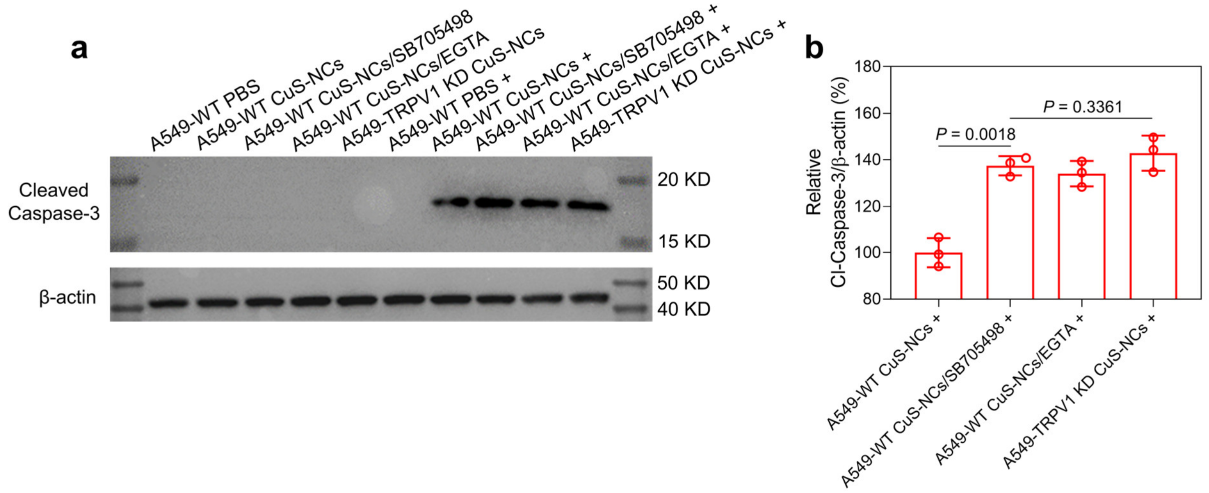

Supplementary Fig. 10. **Western blot analysis of apoptotic proteins.** Western blot (**a**) and quantitative analysis (**b**) of cleaved caspase-3 expression in A549-WT and A549-TRPV1 KD cells treated with or without the hyperthermia from CuS-NCs in the presence or absence of SB705498 (20.0 nM) or EGTA (2.0 mM) (n = 3 independent experiments). Data are presented as mean ± SD. Statistical significance was determined by one-way ANOVA with Tukey's post hoc test.

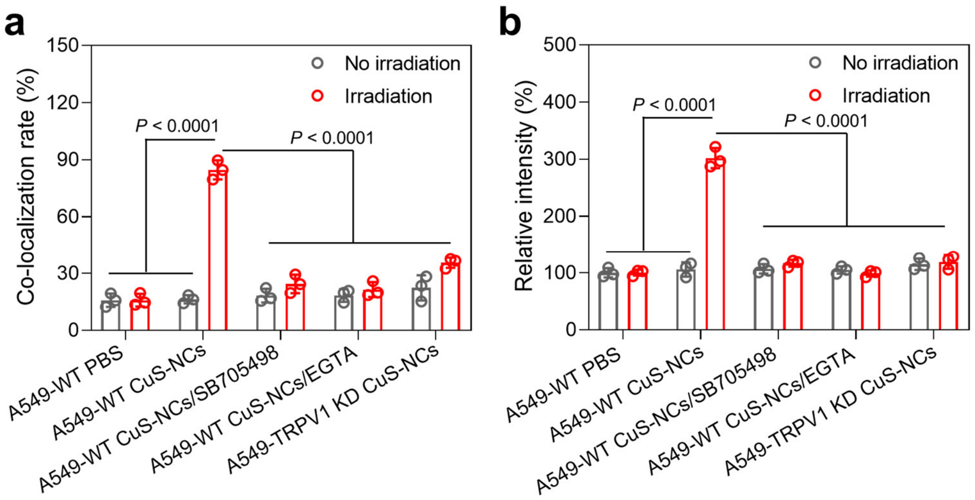

Supplementary Fig. 11. **Quantitative analysis of intranuclear HSF1 and intracellular HSP70.** Co-localization rate of HSF1 and nucleus (**a**) and relative fluorescence intensity of HSP70 (**b**) in A549-WT cells and A549-TRPV1 KD cells treated with or without the hyperthermia from CuS-NCs in the presence or absence of SB705498 (20.0 nM) or EGTA (2.0 mM) (n = 3 biological replicates). Data are presented as mean ± SD. Statistical significance was determined by one-way ANOVA with Tukey's post hoc test.

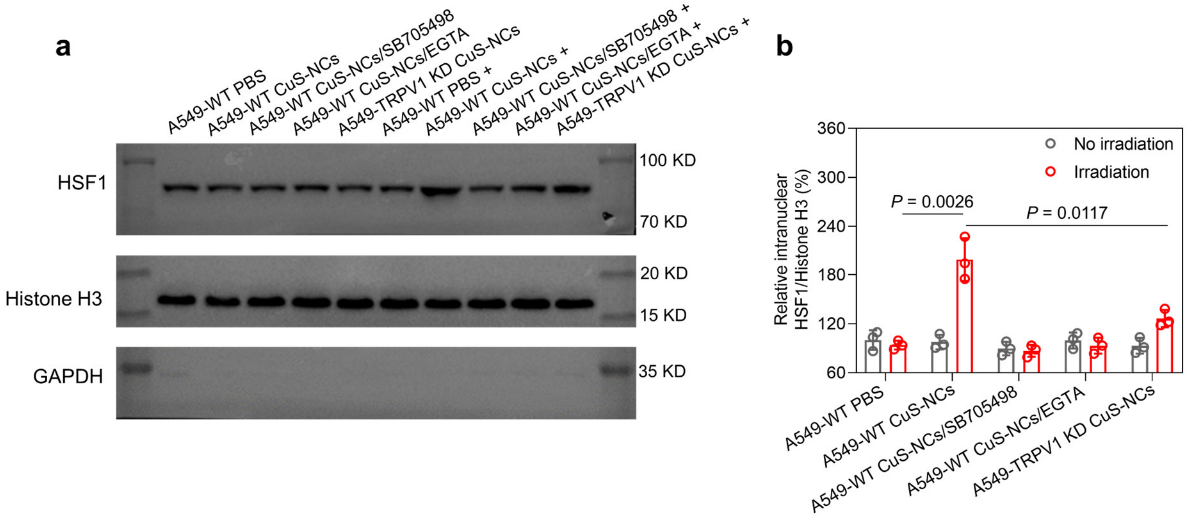

Supplementary Fig. 12. **Western blot analysis of intranuclear HSF1.** Western blot (**a**) and quantitative analysis (**b**) of intranuclear HSF1 expression in A549-WT or A549-TRPV1 KD cells treated with or without the hyperthermia from CuS-NCs in the presence or absence of SB705498 (20.0 nM) or EGTA (2.0 mM) (n = 3 independent experiments). Data are presented as mean ± SD. Statistical significance was determined by one-way ANOVA with Tukey's post hoc test.

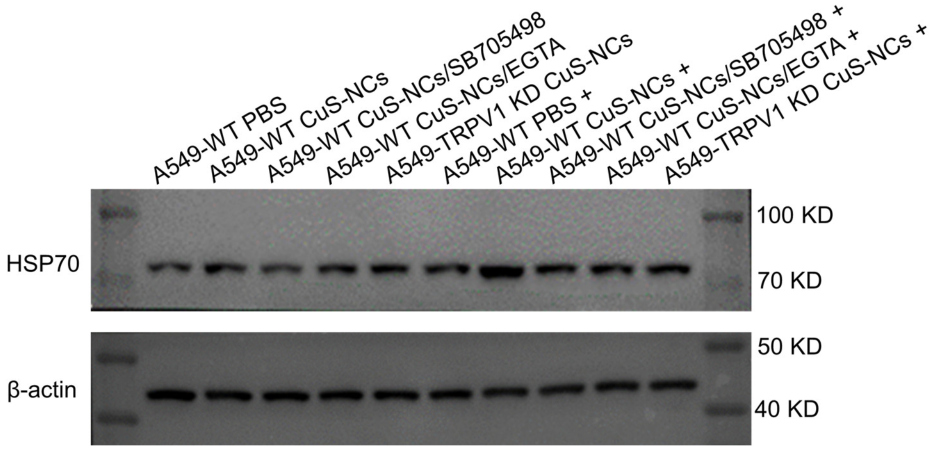

Supplementary Fig. 13. HSP70 expression in A549-WT or A549-TRPV1 KD cells treated with or without the hyperthermia from CuS-NCs in the presence or absence of SB705498 (20.0 nM) or EGTA (2.0 mM). HSP70 could be distinctly suppressed *via* TRPV1 knockdown or blockade upon hyperthermia. This experiment was repeated three times independently with similar results.

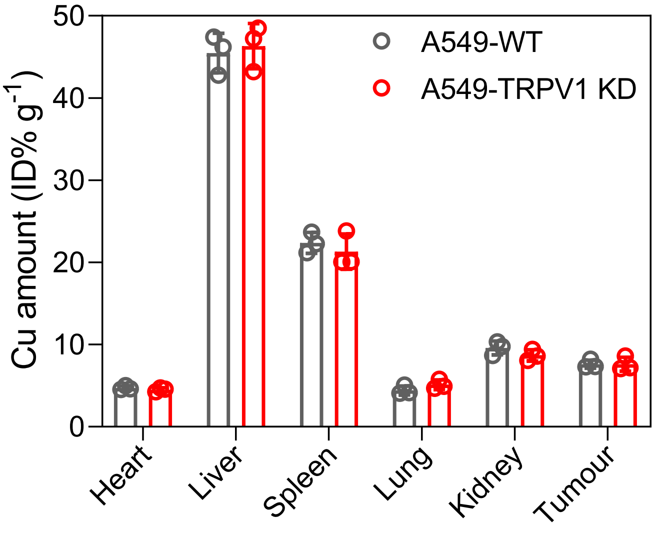

Supplementary Fig. 14. Biodistribution of CuS-NCs in subcutaneous A549-WT and A549-TRPV1 KD tumour-bearing mice at 24 h post-injection of 30.0 μmol kg^{-1} Cu (n = 3 mice per group). Data are presented as mean ± SD.

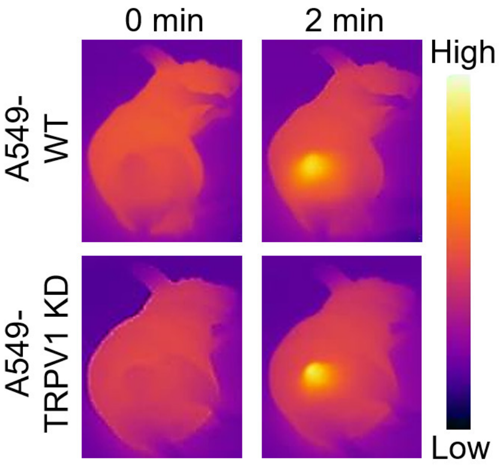

Supplementary Fig. 15. Infrared thermography of mice bearing subcutaneous A549-WT and A549-TRPV1 KD tumours after treatment with CuS-NCs (30.0 μmol kg^{-1} Cu) at 24 h post-injection under light exposure (Low represents 15 ºC and High represents 50 ºC) (n = 3 mice per group).

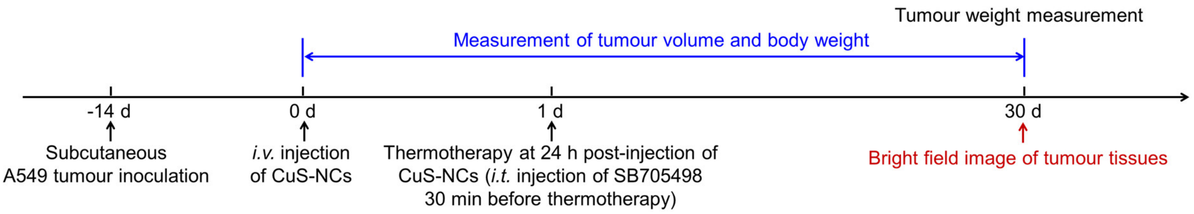

Supplementary Fig. 16. Timeline for the treatment of subcutaneous A549-WT and A549-TRPV1 KD tumour models.

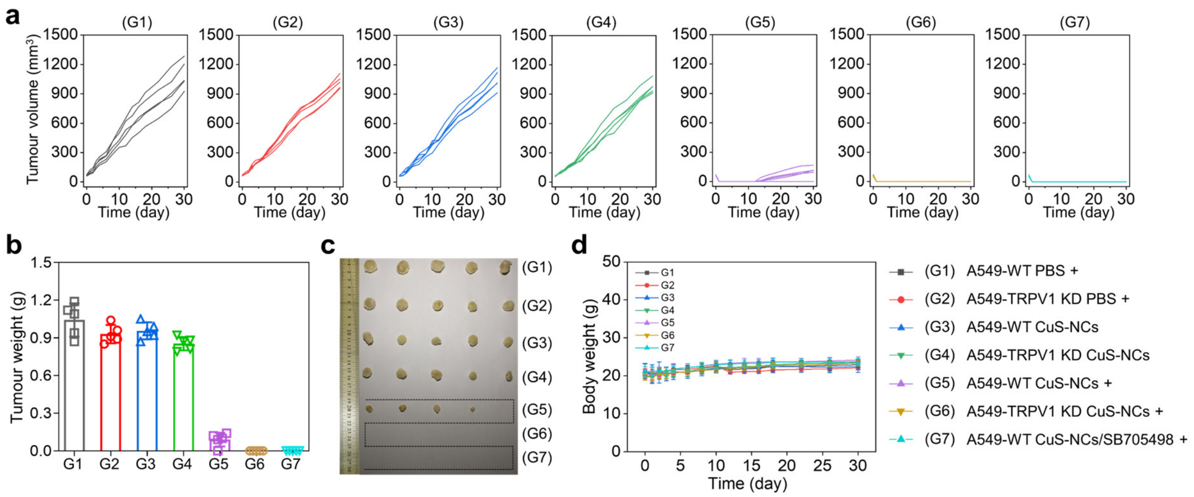

Supplementary Fig. 17. In vivo antitumour efficacy of TRPV1 blockade-synergized thermotherapy against A549-WT and A549-TRPV1 KD tumours. Individual tumour growth profiles (**a**), tumour weight (**b**), tumour photos (**c**), and body weight (**d**) of mice bearing subcutaneous A549-WT and A549-TRPV1 KD tumours treated with the hyperthermia from CuS-NCs (30.0 μmol kg^{-1} Cu) in the presence or absence of intratumoural injection of 1.0 mg kg^{-1} SB705498 (n = 5 mice per group). Data are presented as mean ± SD (**b, d**).

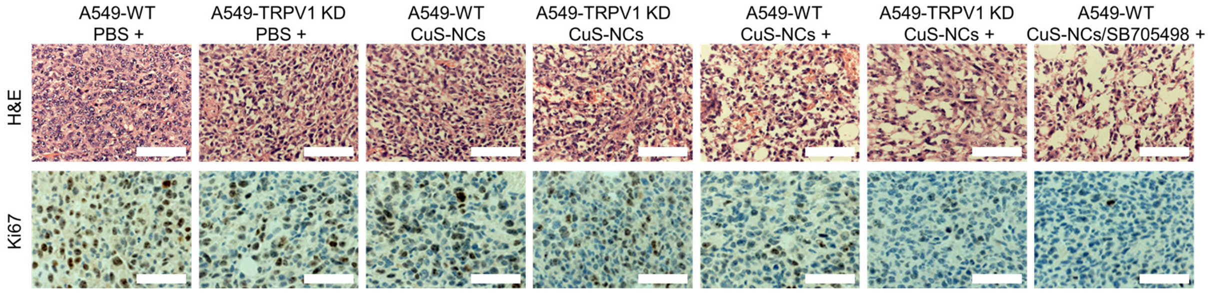

Supplementary Fig. 18. H&E and Ki67 stainings of tumour sections from the mice bearing subcutaneous A549-WT and A549-TRPV1 KD tumours treated with the hyperthermia from CuS-NCs (30.0 μmol kg^{-1} Cu) in the presence or absence of SB705498. Scale bars, 100 μm. These experiments were repeated three times independently with similar results.

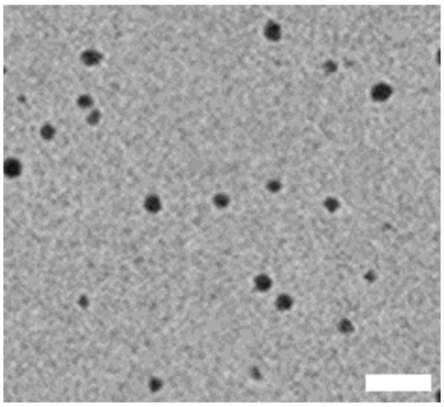

Supplementary Fig. 19. TEM image of I-Micelles with the diameter of 43.2 ± 14.5 nm. Scale bar, 200 nm. This experiment was repeated three times independently with similar results.

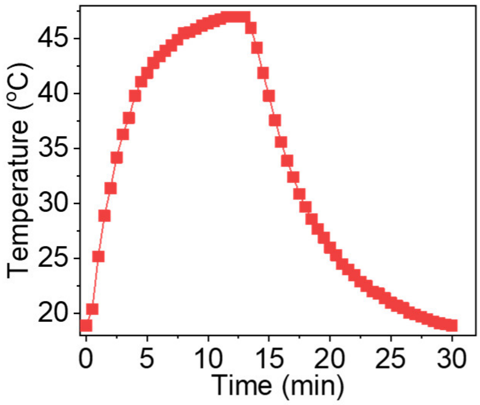

Supplementary Fig. 20. Photothermal conversion efficiency of IS-Micelles. IS-Micelles showed the photothermal conversion efficiency of 22.7%. This experiment was repeated three times independently with similar results.

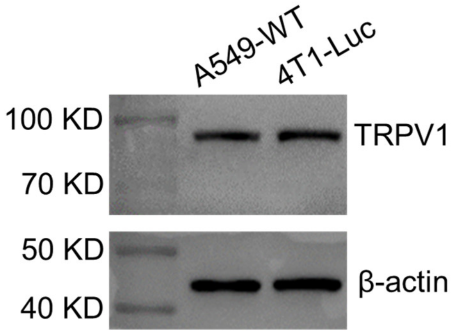

Supplementary Fig. 21. TRPV1 expression in A549-WT and 4T1-Luc cancer cells. These two cells possessed similar TRPV1 expression. This experiment was repeated three times independently with similar results.

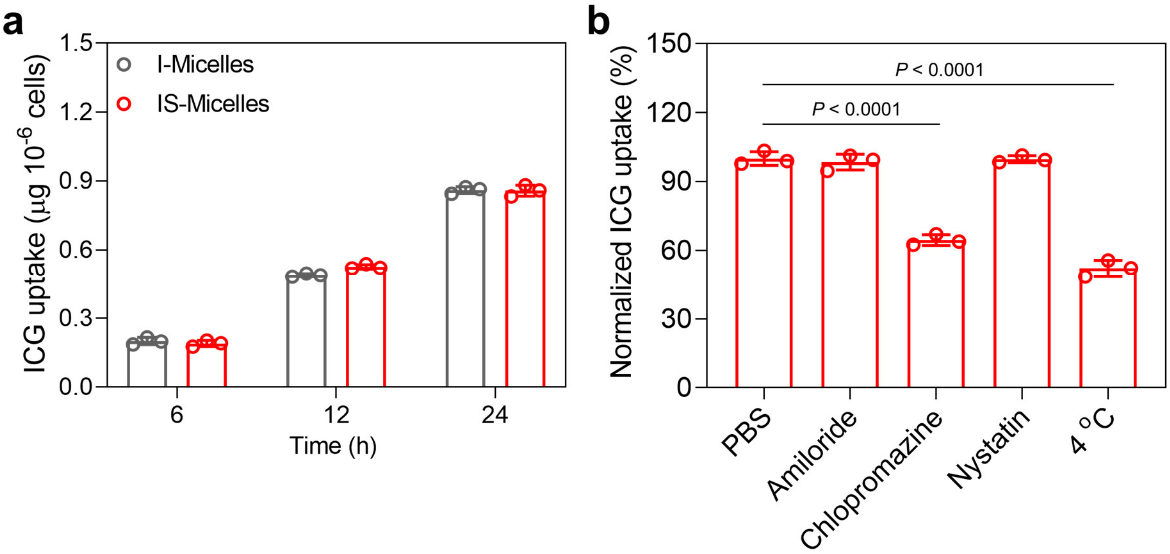

Supplementary Fig. 22. **Cellular uptake and endocytic pathway of IS-Micelles. a** Cellular uptake of IS-Micelles and I-Micelles by 4T1-Luc cells at 6, 12 and 24 h, respectively. I-Micelles and IS-Micelles indicated similar uptake behaviors (n = 3 biological replicates). **b** Normalized amount of ICG internalized by 4T1-Luc tumour cells treated with PBS (control), chlorpromazine, nystatin, amiloride at 37 ºC or 4 ºC. IS-Micelles were endocytosed into 4T1-Luc tumour cells via clathrin-mediated endocytosis pathways (n = 3 biological replicates). Data are presented as mean ± SD (**a, b**). Statistical significance was determined by one-way ANOVA with Tukey's post hoc test.

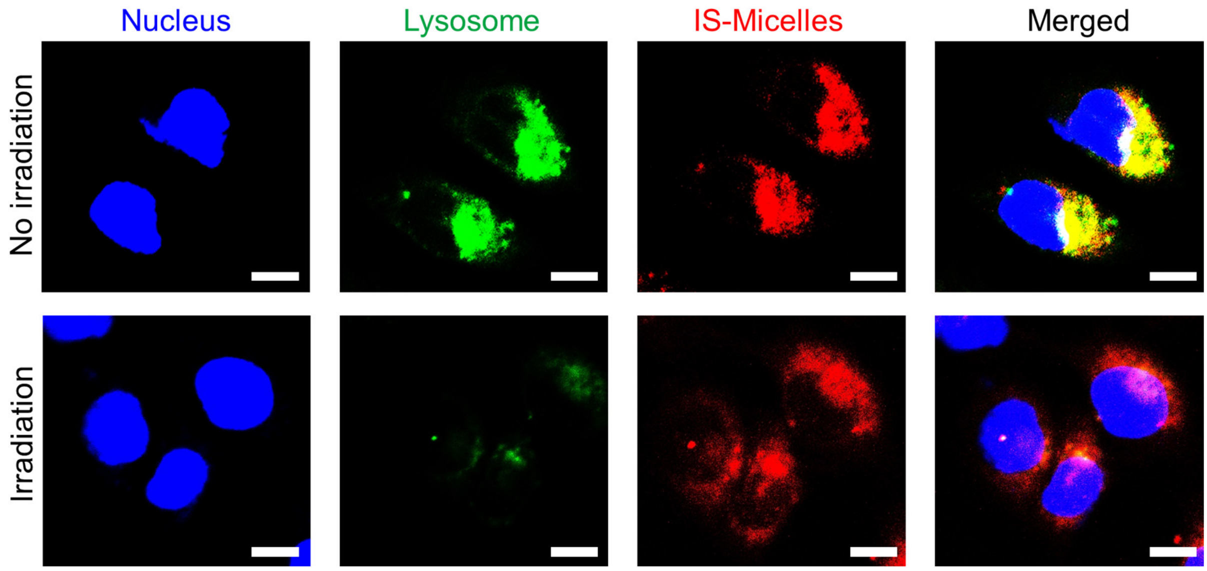

Supplementary Fig. 23. Subcellular distribution of IS-Micelles in 4T1-Luc tumour cells after irradiation or not. Nucleus and lysosome were stained with 1.0 μg mL^{-1} Hoechst 33342 (blue) and 100.0 nM LysoTracker Green (green), respectively. Upon irradiation, the red fluorescence from IS-Micelles appeared in the cytoplasm, and decreased co-localization rate with the lysosome (green) indicated the cytoplasmic translocation of IS-Micelles. Scale bars, 10 μm. This experiment was repeated three times independently with similar results.

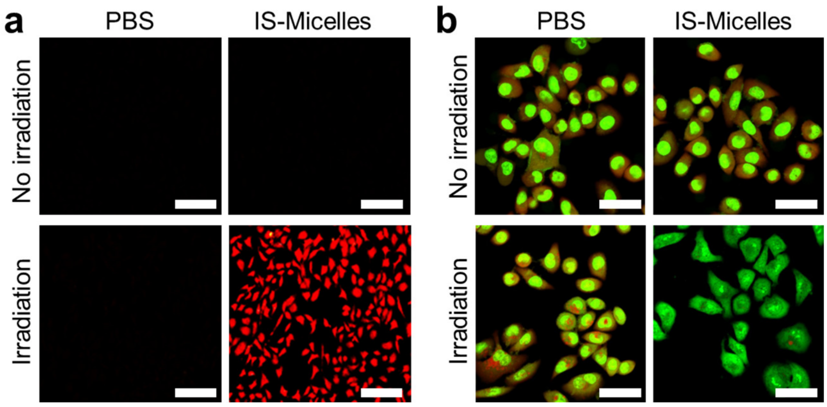

Supplementary Fig. 24. **Intracellular ROS production and lysosomal membrane integrity assessment. a** DHE staining (red) of IS-Micelles in 4T1-Luc tumour cells after irradiation or not. DHE (5.0 μM) was applied as the intracellular ROS probe to evaluate intracellular ROS content (red). Red fluorescence represented the efficient generation of ROS in IS-Micelles upon irradiation. Scale bars, 200 μm. **b** AO staining of IS-Micelles in 4T1-Luc tumour cells after irradiation or not. AO (6.0 μM) was applied as a fluorescent probe to evaluate the integrity of acidic organelles. Red fluorescence represented the integrity of acidic organelles (lysosomes) and the disappearance of red fluorescence indicated the membrane rupture of acidic organelles. Scale bars, 50 μm. These experiments were repeated three times independently with similar results.

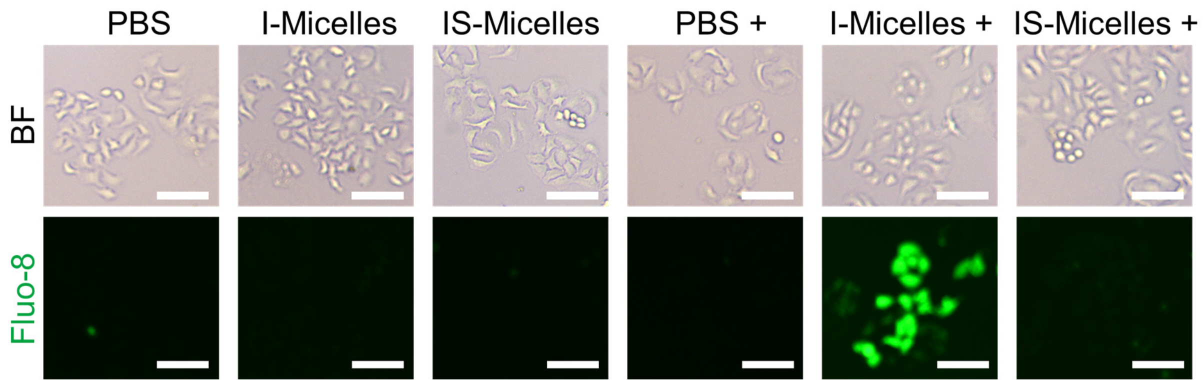

Supplementary Fig. 25. Ca^{2+} influx of 4T1-Luc tumour cells treated with PBS, I-Micelles or IS-Micelles for 24 h upon light exposure (+) or not using Fluo-8 as the calcium probe (green). Green fluorescence represented obvious calcium influx in I-Micelles group under light exposure. Scale bars, 100 μm. This experiment was repeated three times independently with similar results.

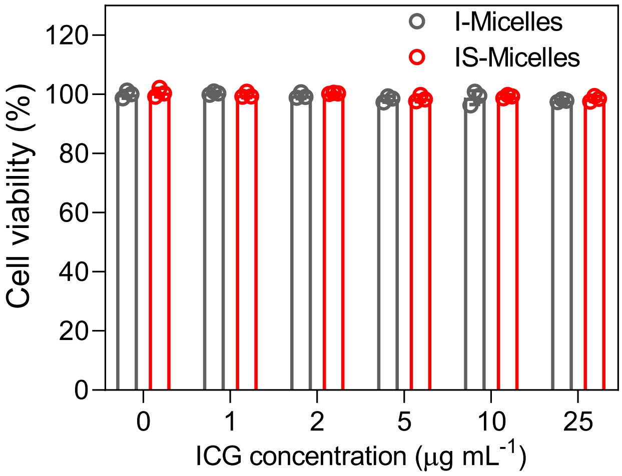

Supplementary Fig. 26. Cell viabilities of 4T1-Luc tumour cells treated with various concentrations of I-Micelles or IS-Micelles for 24 h. I-Micelles and IS-Micelles possessed negligible dark cytotoxicity (n = 3 biological replicates). Data are presented as mean ± SD.

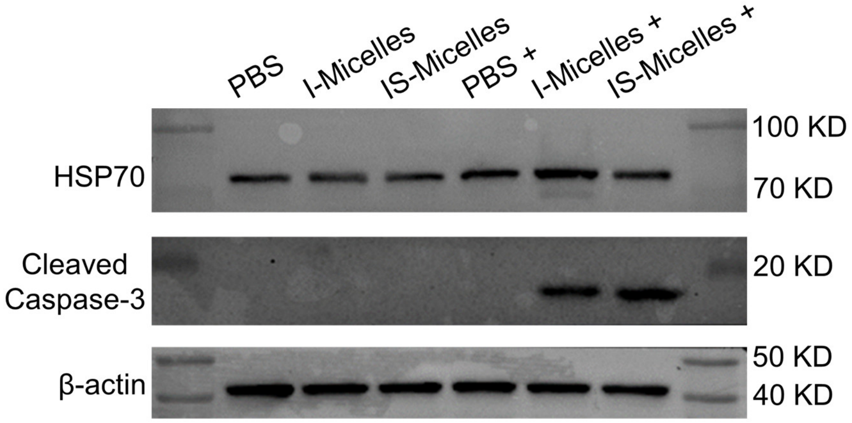

Supplementary Fig. 27. HSP70 and cleaved caspase-3 expressions in 4T1-Luc tumour cells treated with PBS, I-Micelles or IS-Micelles under light exposure (+) or not. HSP70 was distinctly suppressed in IS-Micelles group upon hyperthermia as compared to I-Micelles due to efficient TRPV1 blockade and apoptotic proteins were obviously upregulated. This experiment was repeated three times independently with similar results.

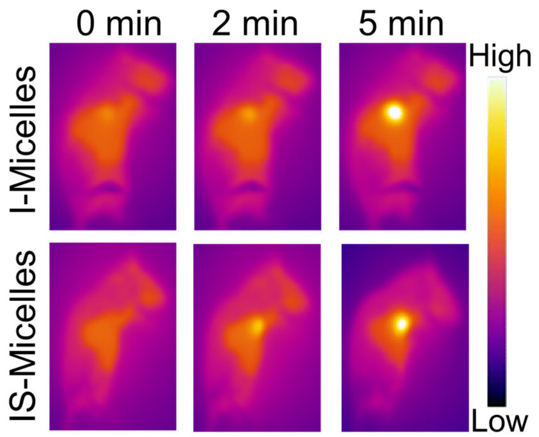

Supplementary Fig. 28. Infrared thermography of orthotopic 4T1-Luc breast tumours at 24 h post-injection of I-Micelles and IS-Micelles (7.5 mg kg^{-1} ICG) upon irradiation for 5 min (n = 3 mice per group, Low represents 15 ºC and High represents 50 ºC).

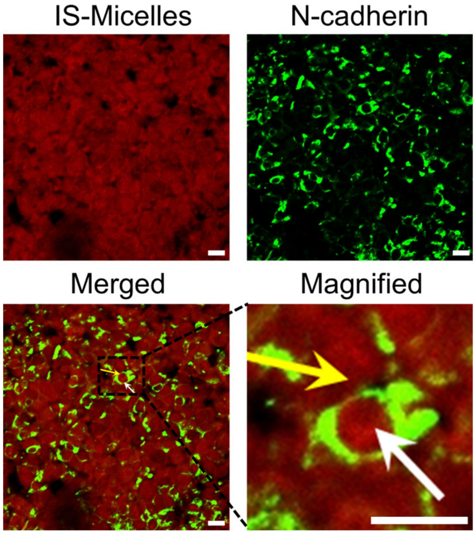

Supplementary Fig. 29. Extracellular and intracellular microdistributions of IS-Micelles (red) in 4T1-Luc breast tumours. The tumour section was labelled with anti-*N*-cadherin antibody and Alexa Flour 488-conjugated secondary antibody for cell membranes staining (green). Yellow arrow and white arrow indicate the extracellular and intracellular distribution of IS-Micelles at tumour site in the magnified image, respectively. Scale bars, 10 μm. This experiment was repeated three times independently with similar results.

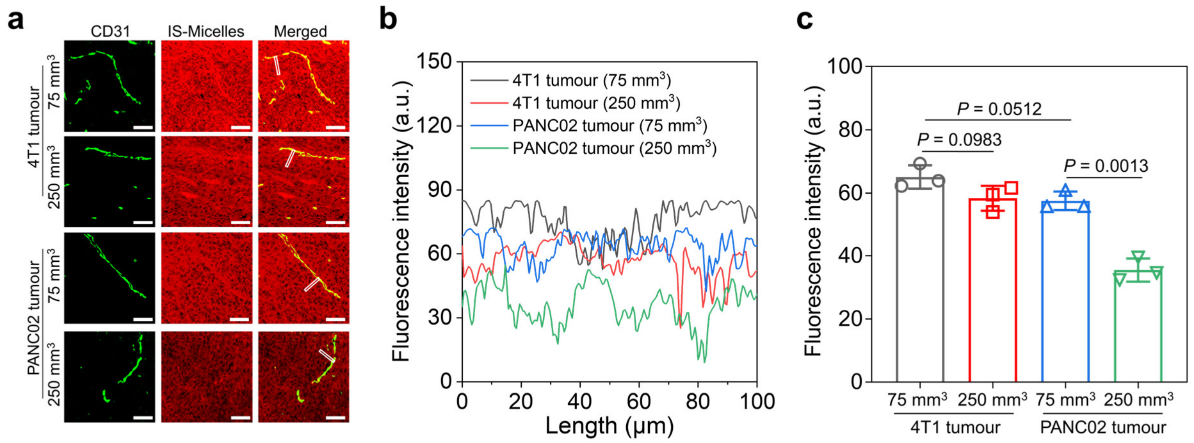

Supplementary Fig. 30. **In vivo penetration of IS-Micelles. a, b** CLSM images of IS-Micelles (red) (**a**) and fluorescence intensity analysis from blood vessel along the white box (**b**) in 4T1 and PANC02 tumour sections from the mice at 24 h post-injection. Tumour blood vessels (green) were stained with anti-CD31 antibody and Alexa Fluor 488 conjugated secondary antibody. Scale bars, 100 μm. **c** Average red fluorescence intensity of IS-Micelles in 4T1 and PANC02 tumour sections from the mice at 24 h post-injection (n = 3 independent experiments). Data are presented as mean ± SD. Statistical significance was determined by one-way ANOVA with Tukey's post hoc test.

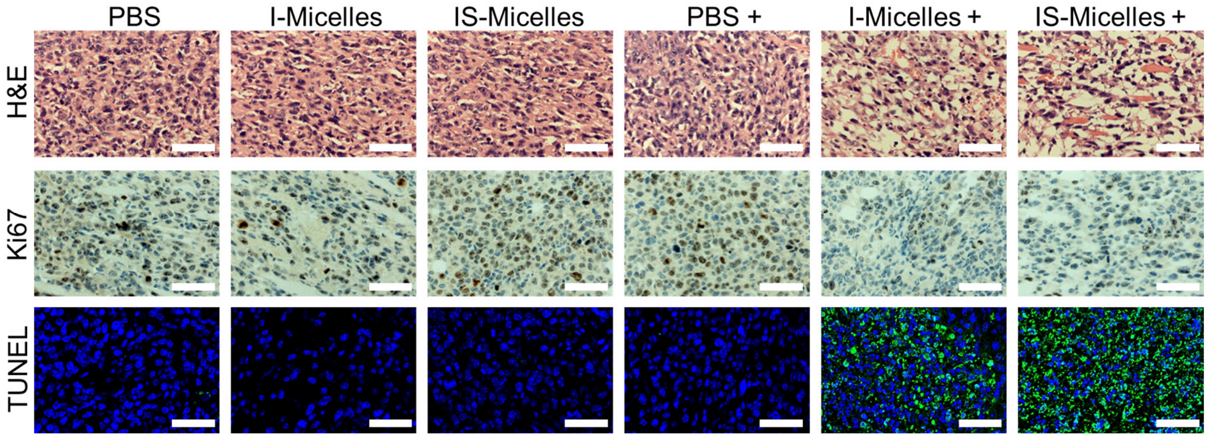

Supplementary Fig. 31. H&E, Ki67, and TUNEL stainings of tumour sections from the mice bearing orthotopic 4T1-Luc breast tumours treated with PBS, I-Micelles or IS-Micelles in the presence or absence of light irradiation (+). Scale bars, 100 μm for H&E and Ki67 stainings, and 50 μm for TUNEL stainings. These experiments were repeated three times independently with similar results.

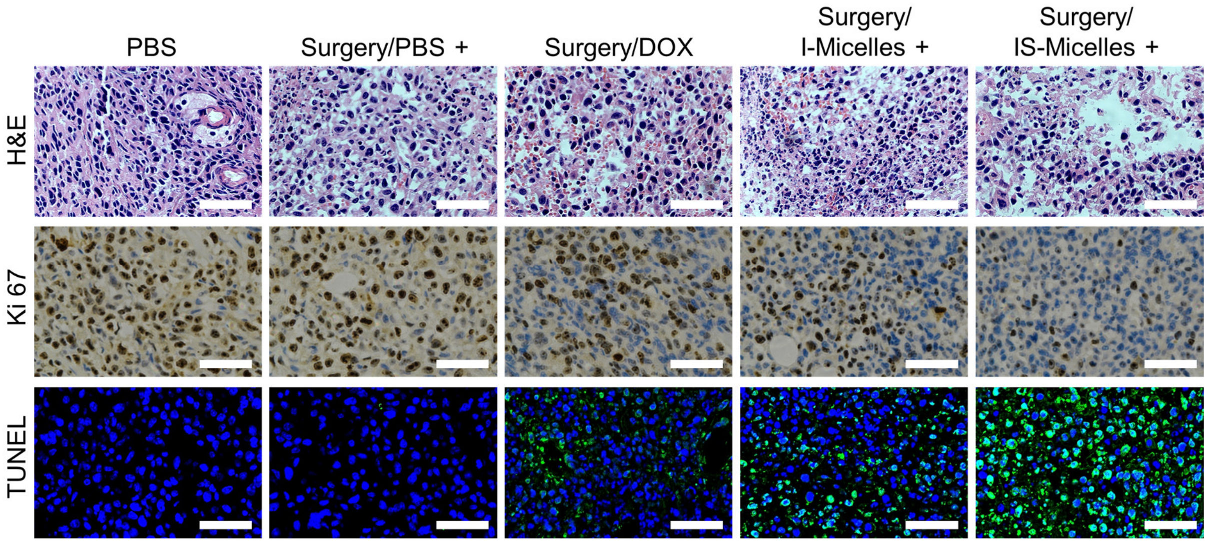

Supplementary Fig. 32. H&E, Ki67 and TUNEL stainings of the tumour sections at 6 h post-treatment of recurrent tumours from the mice bearing orthotopic 4T1-Luc tumours. Scale bars, 100 μm for H&E and Ki67 stainings, and 50 μm for TUNEL stainings. These experiments were repeated three times independently with similar results.

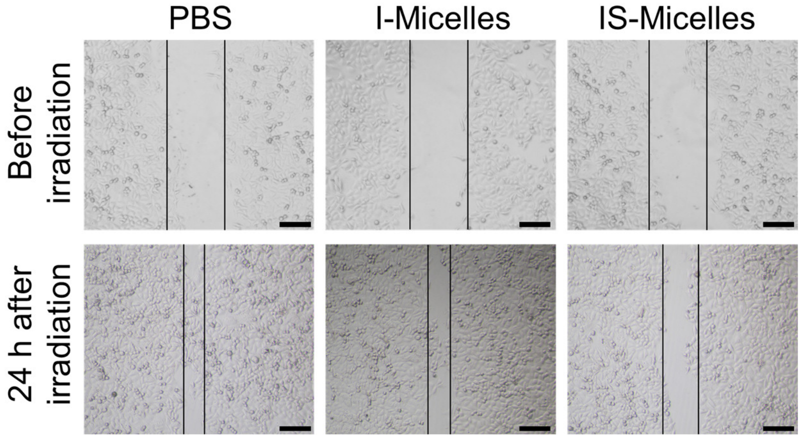

Supplementary Fig. 33. Cell scratch assay of 4T1-Luc breast tumour cells treated with PBS, I-Micelles or IS-Micelles upon light irradiation. The largest distance between tumour cells in the group of IS-Micelles upon irradiation indicates the ability to prevent cell metastasis *via* TRPV1 blockade-synergized thermotherapy. Scale bars, 200 μm. This experiment was repeated three times independently with similar results.

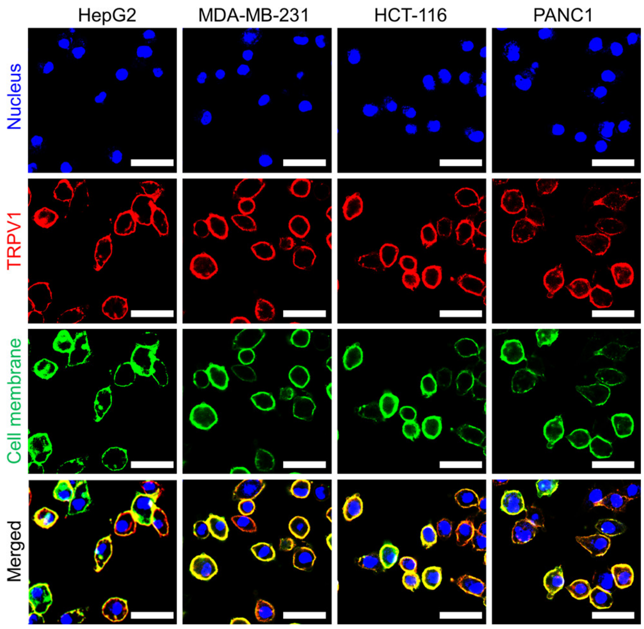

Supplementary Fig. 34. CLSM images of TRPV1 (Alexa Fluor 594-labelled anti-TRPV1 antibody, red) and cell membrane (DiO, green) in the HepG2, MDA-MB-231, HCT-116 and PANC1 cancer cells. Scale bars, 20 μm. This experiment was repeated three times independently with similar results.

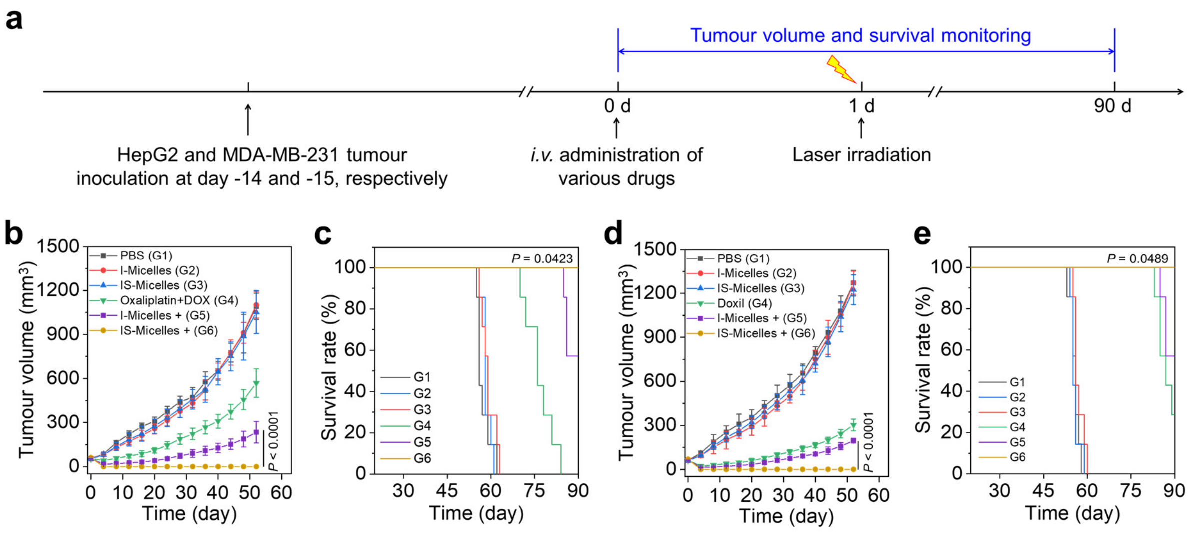

Supplementary Fig. 35. **In vivo antitumour efficacy of IS-Micelles against HepG2 and MDA-MB-231 tumour models. a** Timeline for the treatment of subcutaneous HepG2 and MDA-MB-231 tumour models. **b, c** Tumour growth profiles (**b**) and survival curves (**c**) of mice bearing subcutaneous HepG2 tumour models treated with PBS, Oxaliplatin/Doxorubicin (DOX), I-Micelles and IS-Micelles in the presence of light irradiation (+) or not, respectively (n = 7 mice per group). **d, e** Tumour growth profiles (**d**) and survival curves (**e**) of mice bearing subcutaneous MDA-MB-231 tumour models treated with PBS, DOX-loaded liposomes (Doxil), I-Micelles and IS-Micelles in the presence of light irradiation (+) or not, respectively (n = 7 mice per group). Data are presented as mean ± SD (**b, d**). For **b** and **d**, statistical significance was determined by one-way ANOVA with Tukey's post hoc test. For **c** and **e**, statistical significance was calculated by log-rank test.

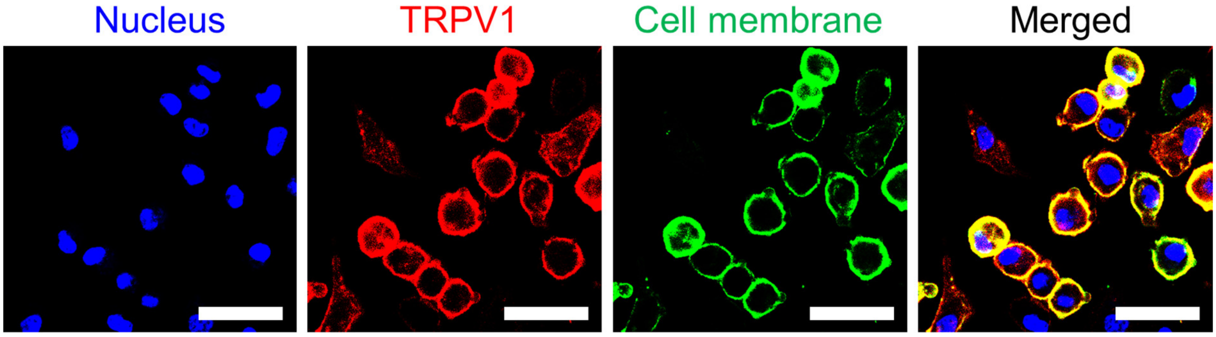

Supplementary Fig. 36. CLSM images of TRPV1 (Alexa Fluor 594-labelled anti-TRPV1 antibody, red) and cell membrane (DiO, green) in the PANC02 cancer cells. Scale bars, 20 μm. This experiment was repeated three times independently with similar results.

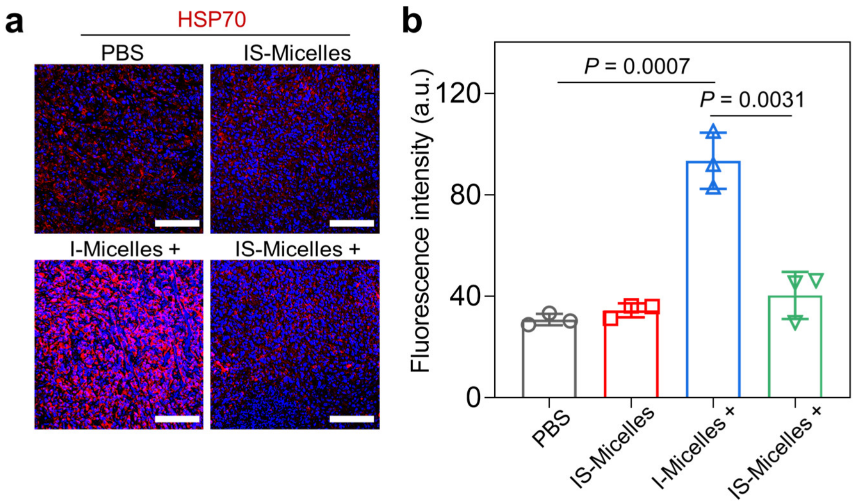

Supplementary Fig. 37. CLSM images (**a**) and fluorescence intensity analysis (**b**) of HSP70 (Alexa Fluor 594-labelled anti-HSP70 antibody, red) in PANC02 tumour sections from the mice treated with PBS, IS-Micelles, I-Micelles under light irradiation (+) and IS-Micelles under light irradiation (+), respectively (n = 3 independent experiments). Scale bars, 100 μm. Data are presented as mean ± SD. Statistical significance was determined by one-way ANOVA with Tukey's post hoc test.

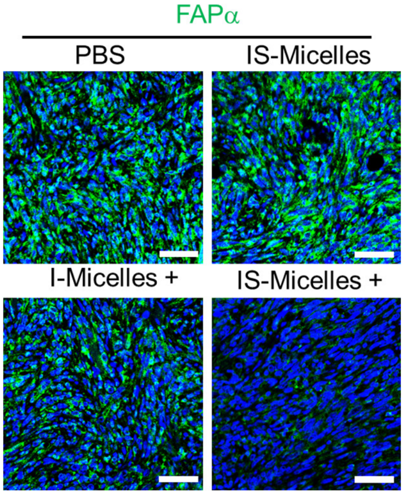

Supplementary Fig. 38. CLSM images of fibroblast activation protein α (FAPα), (Alexa Fluor 488-labelled anti-FAPα antibody, green) of PANC02 tumour sections from the mice treated with PBS, IS-Micelles, I-Micelles under light irradiation (+) and IS-Micelles under light irradiation (+), respectively. Scale bars, 50 µm. This experiment was repeated three times independently with similar results.

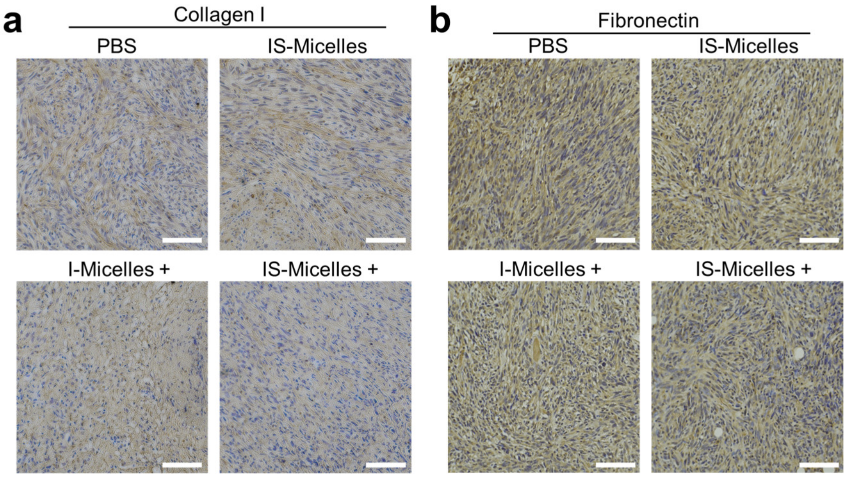

Supplementary Fig. 39. Immunohistochemical stainings of collagen I (**a**) and fibronectin (**b**) using anti-collagen I or anti-fibronectin antibody of PANC02 tumour sections from the mice treated with PBS, IS-Micelles, I-Micelles under light irradiation (+) and IS-Micelles under light irradiation (+), respectively. Scale bars, 100 μm. These experiments were repeated three times independently with similar results.

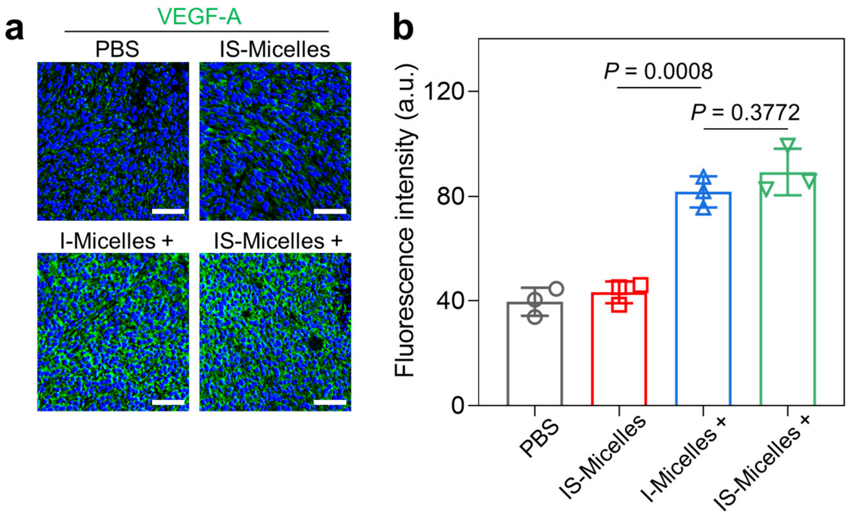

Supplementary Fig. 40. CLSM images of VEGF-A (Alexa Fluor 488-labelled anti-VEGF-A antibody, green) (**a**) and corresponding fluorescence intensity analysis (**b**) of the PNAC02 tumour sections from the mice treated with PBS, IS-Micelles, I-Micelles under light irradiation (+) and IS-Micelles under light irradiation (+), respectively (n = 3 independent experiments). Scale bars, 50 μm. Data are presented as mean ± SD. Statistical significance was determined by one-way ANOVA with Tukey's post hoc test.

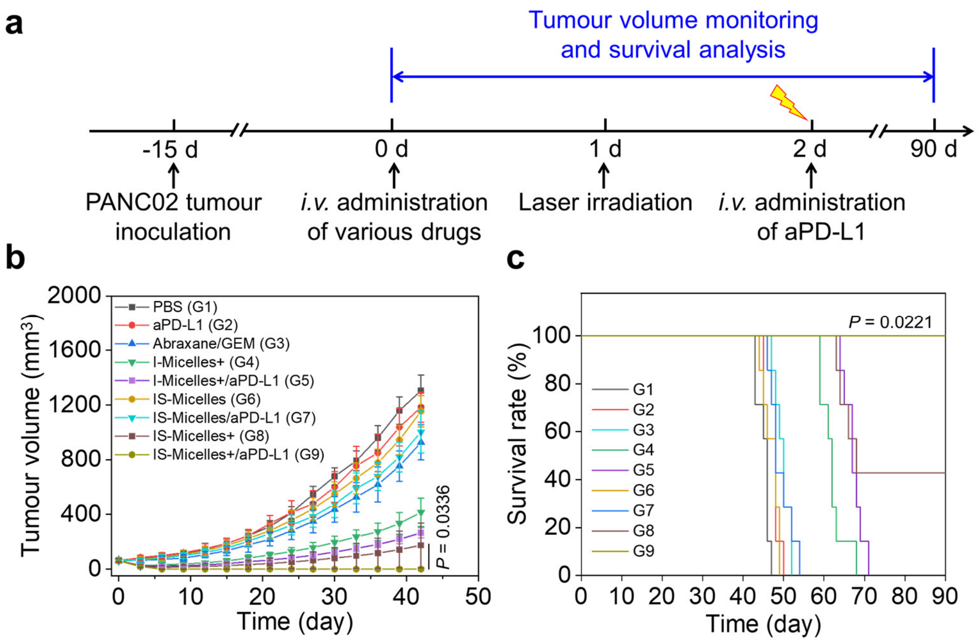

Supplementary Fig. 41. **In vivo antitumour efficacy of IS-Micelles/aPD-L1 against small PANC02 tumour models. a** Timeline for the treatment of subcutaneous PANC02 tumour models. **b, c** Tumour growth profiles (**b**) and survival curves (**c**) of the mice bearing subcutaneous PANC02 tumour models treated with different formulations. The mice were treated with PBS (G1), aPD-L1 (G2), Abraxane/GEM (G3), I-Micelles under light irradiation (+) (G4), I-Micelles/aPD-L1 under light irradiation (+) (G5), IS-Micelles (G6), IS-Micelles/aPD-L1 (G7), IS-Micelles under light irradiation (+) (G8), and IS-Micelles/aPD-L1 under light irradiation (+) (G9), respectively (n = 7 mice per group). Data are presented as mean ± SD. For **b**, statistical significance was determined by one-way ANOVA with Tukey's post hoc test. For **c**, statistical significance was calculated by log-rank test.

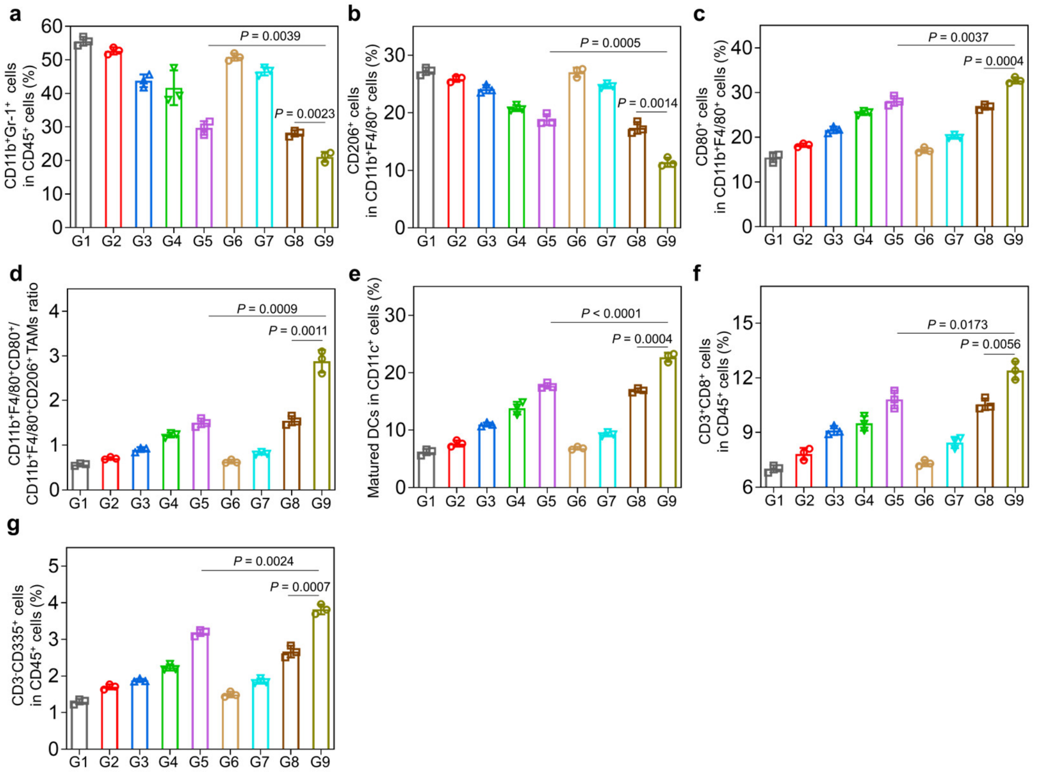

Supplementary Fig. 42. **In vivo immune response of IS-Micelles/aPD-L1 in small PANC02 tumour models. a-c** Quantification of CD45$^+$CD11b$^+$Gr-1$^+$ MDSCs (**a**), CD11b$^+$F4/80$^+$CD206$^+$ TAMs (**b**) and CD11b$^+$F4/80$^+$CD80$^+$ TAMs (**c**) inside PANC02 tumours (50-75 mm^3) at 72 h post-treatment with various formulations. **d** Ratio of CD11b$^+$F4/80$^+$CD80$^+$ TAMs to CD11b$^+$F4/80$^+$CD206$^+$ TAMs inside PANC02 tumours at 72 h post-treatment with various formulations. **e** Quantification of matured dendritic cells (CD11c$^+$CD80$^+$CD86$^+$ DCs) inside tumour-draining lymph nodes at 72 h post-treatment with various formulations. **f, g** Quantification of tumour-infiltrating CD45$^+$CD3$^+$CD8$^+$ CTLs (**f**) and CD45$^+$CD3$^-$CD335$^+$ NK cells (**g**) in PANC02 tumours at 72 h post-treatment with various formulations. The mice were treated with PBS (G1), aPD-L1 (G2), Abraxane/GEM (G3), I-Micelles under light irradiation (+) (G4), I-Micelles/aPD-L1 under light irradiation (+) (G5), IS-Micelles (G6), IS-Micelles/aPD-L1 (G7), IS-Micelles under light irradiation (+) (G8), and IS-Micelles/aPD-L1 under light irradiation (+) (G9), respectively (n = 3 mice per group). Data are presented as mean ± SD (**a-g**). Statistical significance was determined by one-way ANOVA with Tukey's post hoc test.

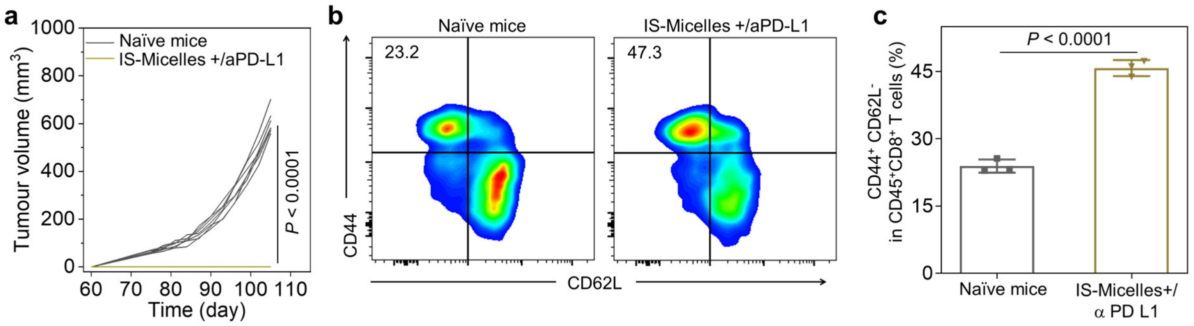

Supplementary Fig. 43. **Immune memory effect of IS-Micelles/aPD-L1. a** Individual tumour growth profiles of rechallenged tumours at 60 d post-administration (n = 7 mice per group). **b, c** Representative flow cytometry dot plot (**l**) and quantification (**m**) of CD44^{hi}CD62L^{low} effector memory T cells in naïve and IS-Micelles (+) plus aPD-L1 treated mice (n = 3 mice per group). Data are presented as mean ± SD. Statistical significance was determined by one-way ANOVA with Tukey's post hoc test.

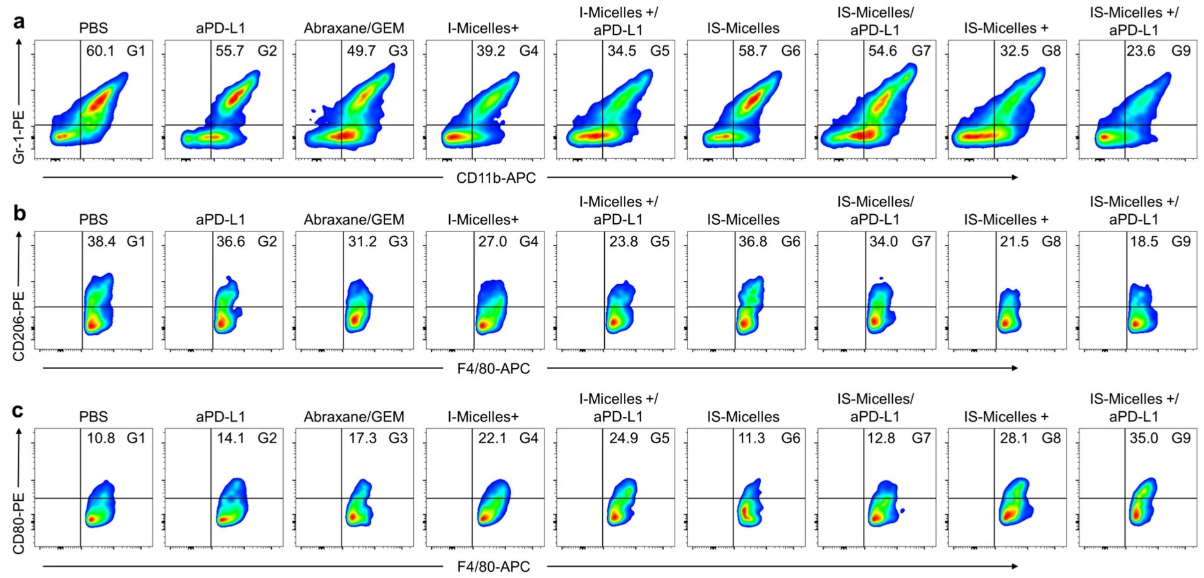

Supplementary Fig. 44. Representative flow cytometry dot plot of tumour-infiltrating $CD45^+CD11b^+Gr\text{-}1^+$ MDSCs (**a**), $CD11b^+F4/80^+CD206^+$ TAMs (**b**), and $CD11b^+F4/80^+CD80^+$ TAMs (**c**) in the mice bearing large PANC02 tumour models (250-300 mm^3) at 72 h post-treatment with PBS, Abraxane/GEM, I-Micelles, and IS-Micelles in the presence of light irradiation (+) and aPD-L1 or not. (n = 5 mice per group).

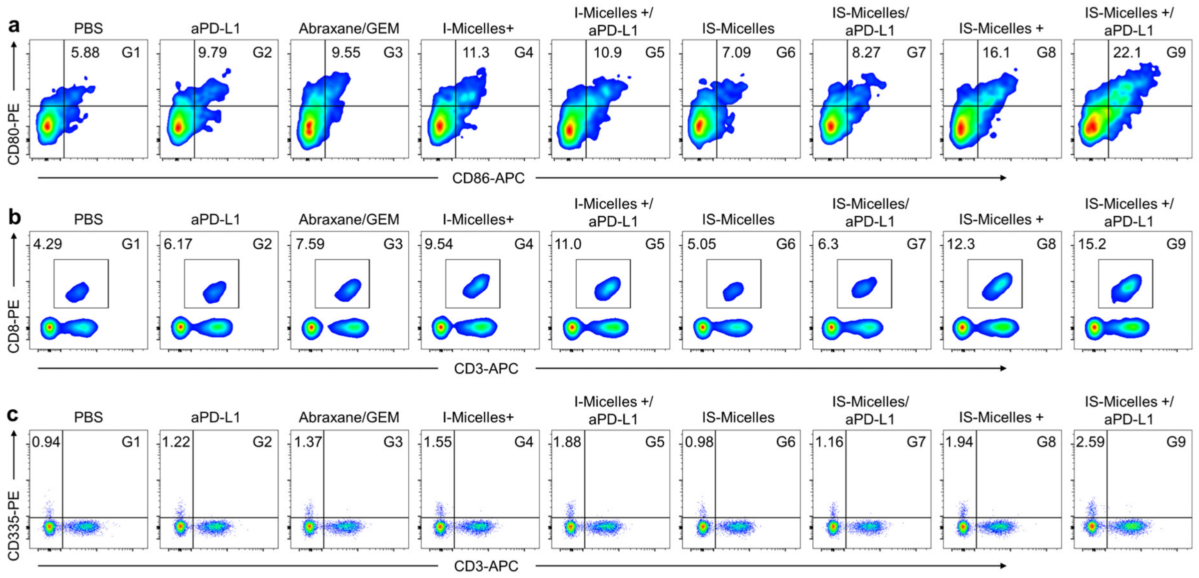

Supplementary Fig. 45. Representative flow cytometry dot plot of CD11c^{+}CD80^{+}CD86^{+} DCs (**a**), tumour-infiltrating CD45^{+}CD3^{+}CD8^{+} CTLs (**b**), and tumour-infiltrating CD45^{+}CD3^{-}CD335^{+} NK cells (**c**) in the mice bearing large PANC02 tumour models (250-300 mm^{3}) at 72 h post-treatment with PBS, Abraxane/GEM, I-Micelles, and IS-Micelles in the presence of light irradiation (+) and aPD-L1 or not (n = 5 mice per group).

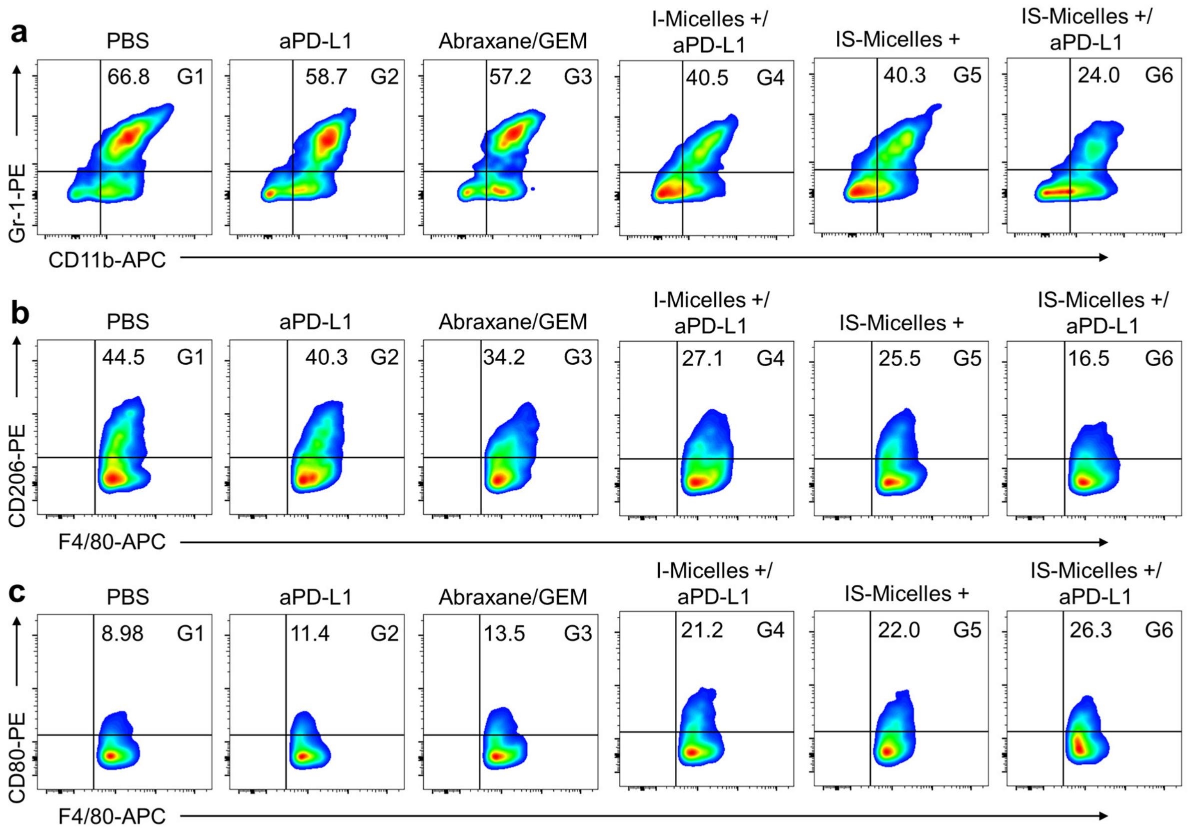

Supplementary Fig. 46. Representative flow cytometry dot plot of tumour-infiltrating CD45$^+$CD11b$^+$Gr-1$^+$ MDSCs (**a**), CD11b$^+$F4/80$^+$CD206$^+$ TAMs (**b**), and CD11b$^+$F4/80$^+$CD80$^+$ TAMs (**c**) in the mice bearing orthotopic PANC02-Luc tumour models at 72 h post-treatment with PBS, Abraxane/GEM, I-Micelles, and IS-Micelles in the presence of light irradiation (+) and aPD-L1 or not (n = 5 mice per group).

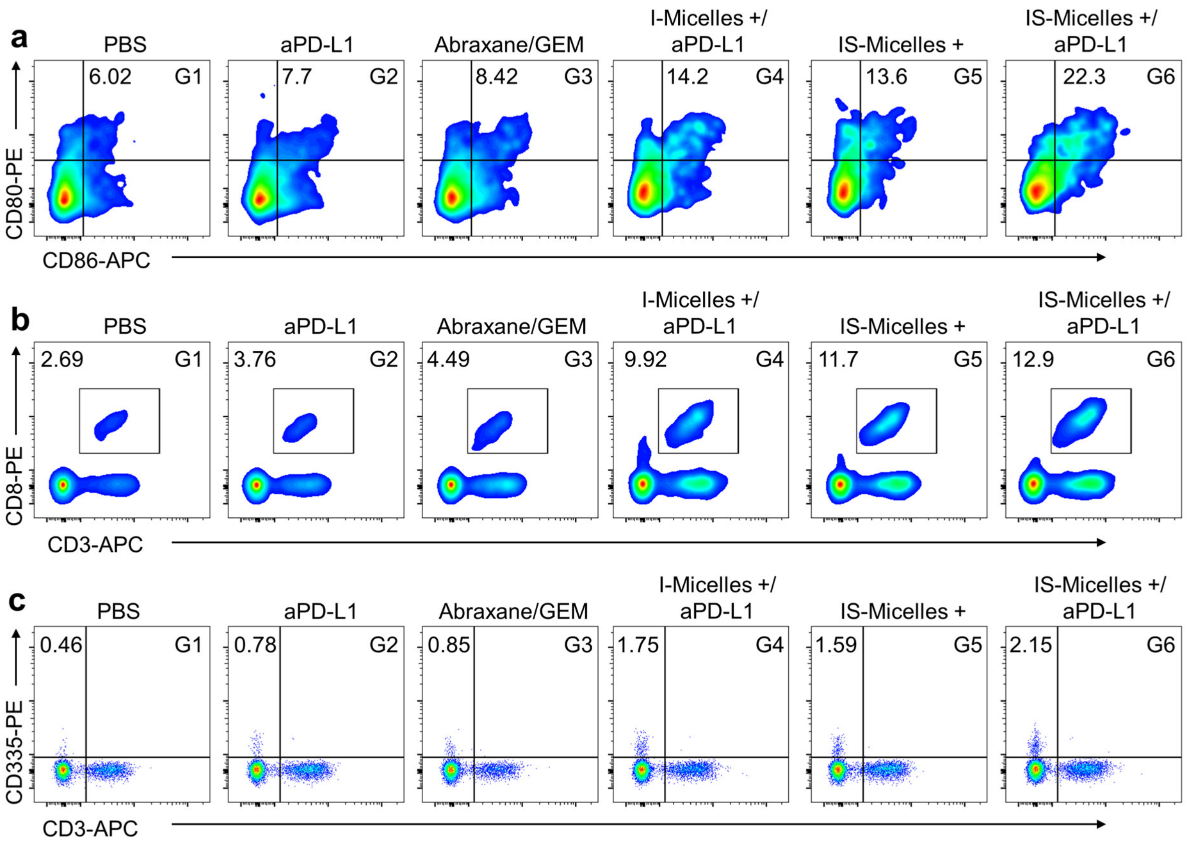

Supplementary Fig. 47. Representative flow cytometry dot plot of $CD11c^{+}CD80^{+}CD86^{+}$ DCs (**a**), tumour-infiltrating $CD45^{+}CD3^{+}CD8^{+}$ CTLs (**b**), and tumour-infiltrating $CD45^{+}CD3^{-}CD335^{+}$ NK cells (**c**) in the mice bearing orthotopic PANC02-Luc tumour models at 72 h post-treatment with PBS, Abraxane/GEM, I-Micelles, and IS-Micelles in the presence of light irradiation (+) and aPD-L1 or not (n = 5 mice per group).

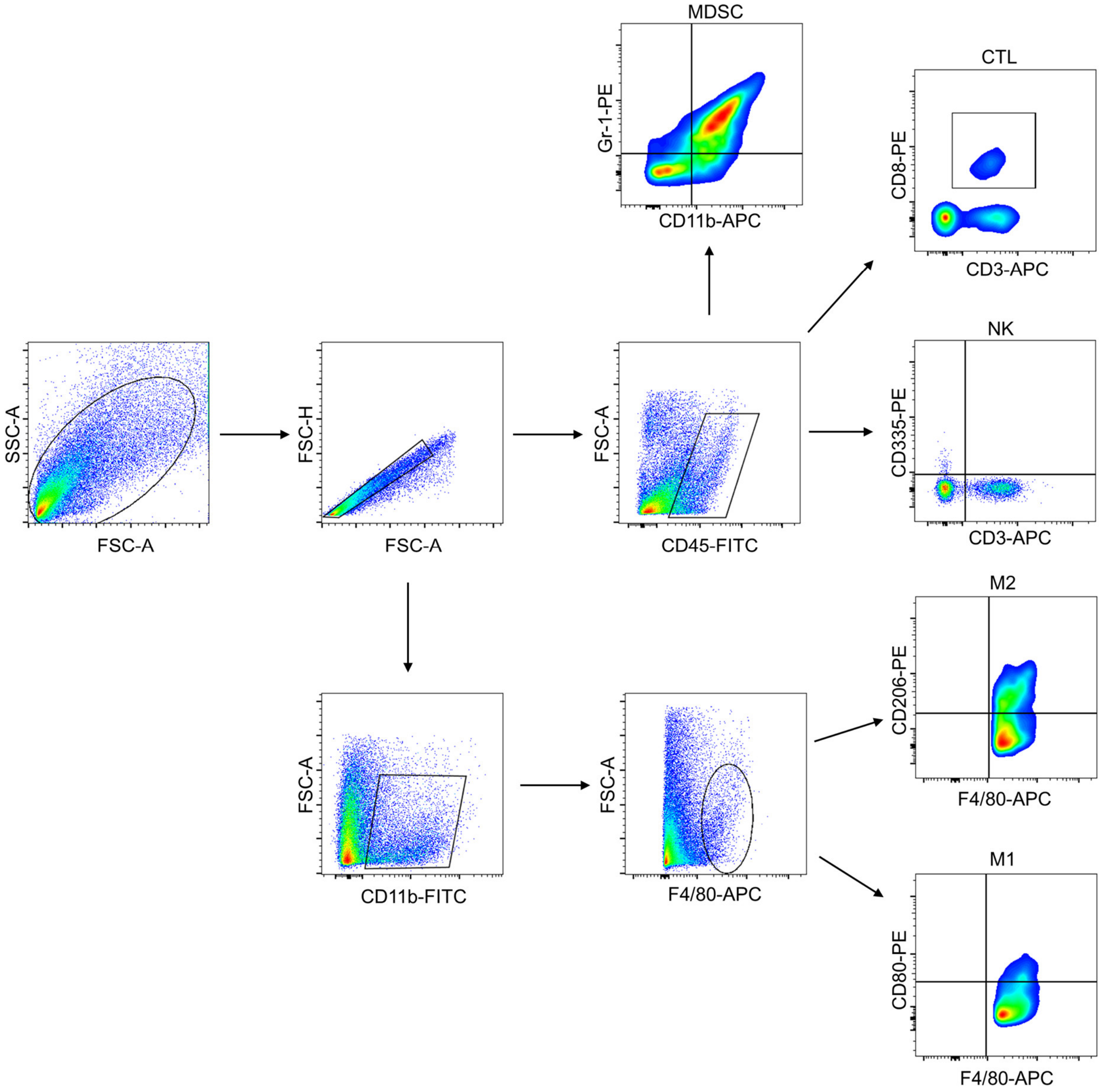

Supplementary Fig. 48. Gating strategy to sort CD11b$^+$Gr-1$^+$ MDSCs in tumour tissues gating in CD45$^+$ cells presented in Fig. 8d and 9e, and Supplementary Fig. 44a and 46a, and tumour-infiltrating CD3$^+$CD8$^+$ T lymphocytes in tumour tissues gating in CD45$^+$ cells presented in Fig. 8i and 9h, and Supplementary Fig. 45b and 47b, and tumour-infiltrating CD3$^-$CD335$^+$ NK cells in tumour tissues gating in CD45$^+$ cells presented in Fig. 8j and 9i, and Supplementary Fig. 45c and 47c, and CD206$^+$ TAMs in tumour tissues gating in CD11b$^+$F4/80$^+$ TAMs presented in Fig. 8e, and Supplementary Fig. 44b and 46b, and CD80$^+$ TAMs in tumour tissues gating in CD11b$^+$F4/80$^+$ TAMs presented in Fig. 8f, Supplementary Fig. 44c and 46c.

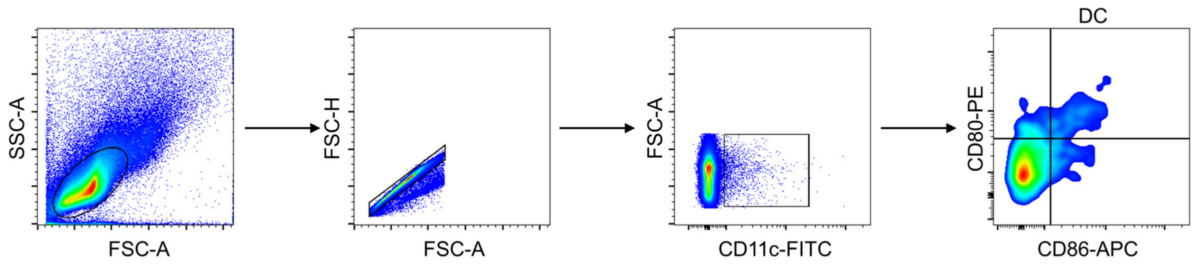

Supplementary Fig. 49. Gating strategy to sort CD80$^+$CD86$^+$ matured dendritic cells in tumour-draining lymph nodes gating in CD11c$^+$ cells presented in Fig. 8h and 9g, Supplementary Fig. 45a and 47a.

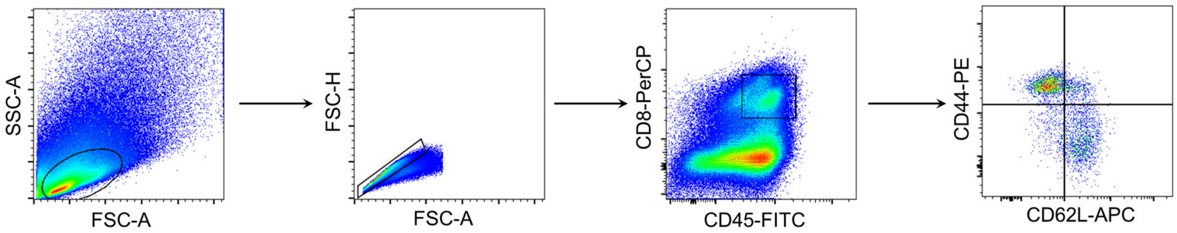

Supplementary Fig. 50. Gating strategy to sort CD44$^+$CD62L$^-$ effector memory T cells in spleen tissues gating in CD45$^+$CD8$^+$ cells presented in supplementary Fig. 43b, c.

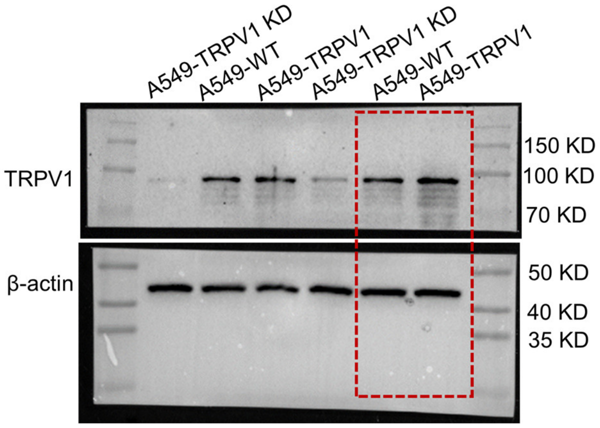

Supplementary Fig. 51. Uncropped scans of blots presented in supplementary Fig. 5a.

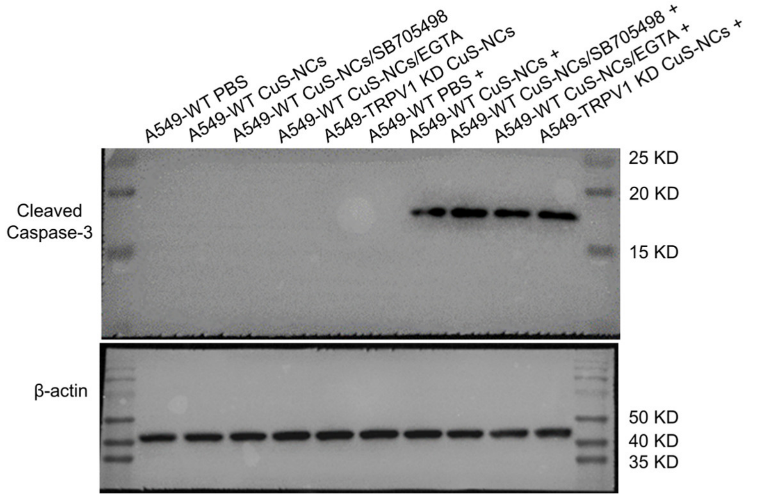

Supplementary Fig. 52. Uncropped scans of blots presented in supplementary Fig. 10a.

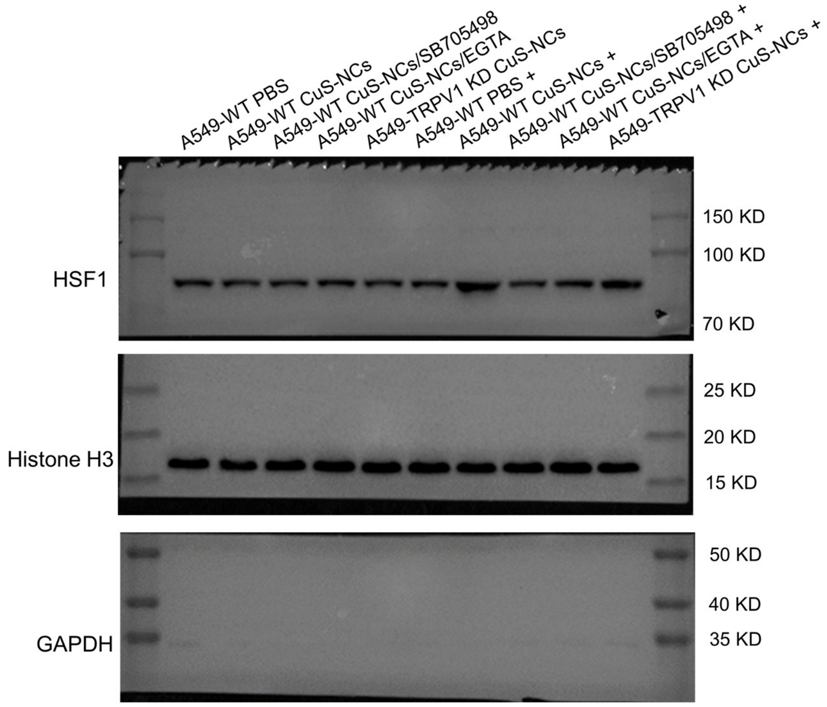

Supplementary Fig. 53. Uncropped scans of blots presented in supplementary Fig. 12a.

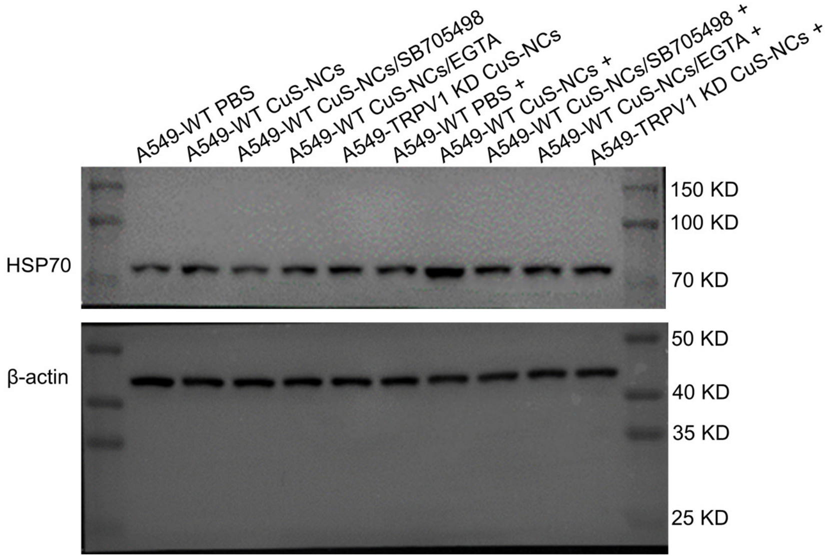

Supplementary Fig. 54. Uncropped scans of blots presented in supplementary Fig. 13.

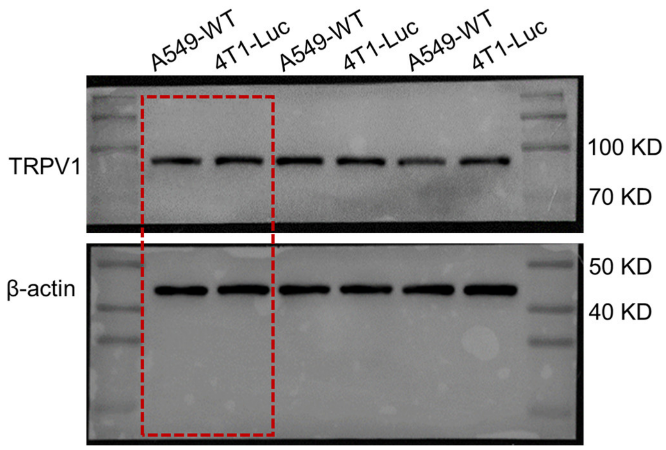

Supplementary Fig. 55. Uncropped scans of blots presented in supplementary Fig. 21.

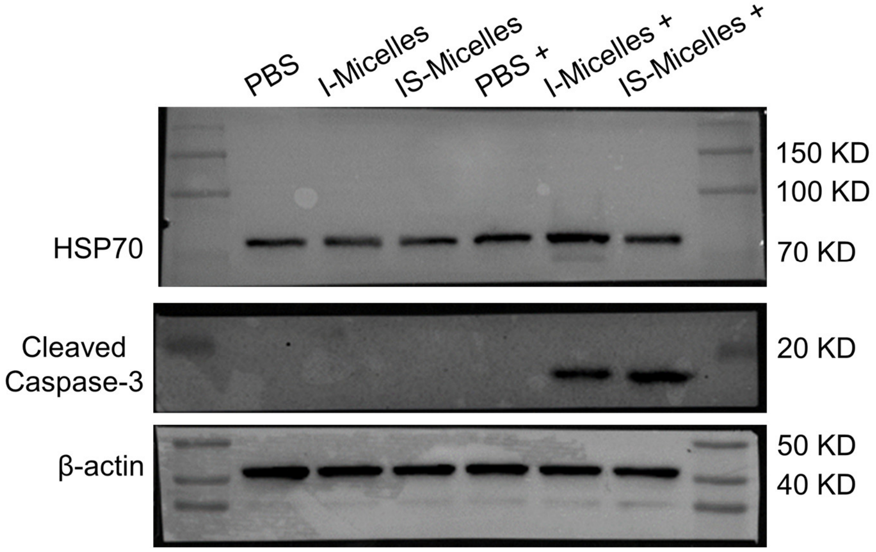

Supplementary Fig. 56. Uncropped scans of blots presented in supplementary Fig. 27.
