# Supplementary material for: Nanoparticle-mediated TRPV1 channel blockade amplifies cancer thermo-immunotherapy via heat shock factor 1 modulation
Source: Nat Commun. 2023 Apr 29;14:2498. doi: 10.1038/s41467-023-38128-x (PMC10148815; doi:10.1038/s41467-023-38128-x)
Supplement: Supplementary file 2 — Reporting Summary [file 41467_2023_38128_MOESM2_ESM.pdf]

nature portfolio

Corresponding author(s): Tao Yang, Xiaohui Sun and Huabing Chen

Last updated by author(s): Apr 4, 2023

Reporting Summary

Nature Portfolio wishes to improve the reproducibility of the work that we publish. This form provides structure for consistency and transparency in reporting. For further information on Nature Portfolio policies, see our Editorial Policies and the Editorial Policy Checklist.

Statistics

For all statistical analyses, confirm that the following items are present in the figure legend, table legend, main text, or Methods section.

n/a	Confirmed	
☐	☒	The exact sample size (*n*) for each experimental group/condition, given as a discrete number and unit of measurement
☐	☒	A statement on whether measurements were taken from distinct samples or whether the same sample was measured repeatedly
☐	☒	The statistical test(s) used AND whether they are one- or two-sided<br>*Only common tests should be described solely by name; describe more complex techniques in the Methods section.*
☒	☐	A description of all covariates tested
☒	☐	A description of any assumptions or corrections, such as tests of normality and adjustment for multiple comparisons
☐	☒	A full description of the statistical parameters including central tendency (e.g. means) or other basic estimates (e.g. regression coefficient) AND variation (e.g. standard deviation) or associated estimates of uncertainty (e.g. confidence intervals)
☐	☒	For null hypothesis testing, the test statistic (e.g. *F*, *t*, *r*) with confidence intervals, effect sizes, degrees of freedom and *P* value noted<br>*Give P values as exact values whenever suitable.*
☒	☐	For Bayesian analysis, information on the choice of priors and Markov chain Monte Carlo settings
☒	☐	For hierarchical and complex designs, identification of the appropriate level for tests and full reporting of outcomes
☒	☐	Estimates of effect sizes (e.g. Cohen's *d*, Pearson's *r*), indicating how they were calculated

Our web collection on statistics for biologists contains articles on many of the points above.

Software and code

Policy information about availability of computer code

Data collection	The morphology was obtained from TEM (HT7700, Hitachi). The size distribution was evaluated by dynamic light scattering (DLS, Zetasizer ZS90, Malvern). UV-vis spectrophotometer (UV2600, Shimadzu) were used to record UV-vis spectra. The amount of ICG was determined by fluorescence spectrophotometer (Fluoromax-4, HORIBA). The concentration of copper ions was determined by inductively coupled plasma optical emission spectrometer (ICP-OES, 710-ES, VARIAN). The concentration of SB705498 was determined by the reversed-phase high-performance liquid chromatography (HPLC) (Agilent 1100, Agilent). The RNA-sequencing data was obtained from NovaSeq 6000 (Illumina). Bioluminescence of lungs and mice were obtained by IVIS Spectrum Lumina III (Perkin Elmer). The infrared thermo-imaging was performed using an infrared camera (Fotric 225, Fotric). All flow cytometry data were obtained by cell analyzer (FACSAria III, BD). All fluorescence images were detected by LSM 710 (Zeiss). All bright-field images were detected by IX73 bright field microscopy (Olympus).
Data analysis	All statistical analyses were performed by Graphpad Prism (Version 8.0.2). All flow cytometry data were analyzed by Flowjo (Version 10). Bioluminescent images were analyzed by Living image software (Version 4.5). Fluorescence images were analyzed by Image J (Version 1.8.0.112). The RNA-sequencing data was analyzed by IPA software (Basic Version). Functional interaction network was analyzed by STRING (Version 11.5).

For manuscripts utilizing custom algorithms or software that are central to the research but not yet described in published literature, software must be made available to editors and reviewers. We strongly encourage code deposition in a community repository (e.g. GitHub). See the Nature Portfolio guidelines for submitting code & software for further information.

Data

Policy information about availability of data

All manuscripts must include a data availability statement. This statement should provide the following information, where applicable:

- Accession codes, unique identifiers, or web links for publicly available datasets
- A description of any restrictions on data availability
- For clinical datasets or third party data, please ensure that the statement adheres to our policy

The data that support the findings of this study are available within the paper and its Supplementary Information files. The RNA-sequencing data generated in this study have been deposited in the Gene Expression Omnibus under the accession number GSE199299. Source data are provided with this paper.

Human research participants

Policy information about studies involving human research participants and Sex and Gender in Research.

Reporting on sex and gender	N/A
Population characteristics	N/A
Recruitment	N/A
Ethics oversight	N/A

Note that full information on the approval of the study protocol must also be provided in the manuscript.

Field-specific reporting

Please select the one below that is the best fit for your research. If you are not sure, read the appropriate sections before making your selection.

☒ Life sciences ☐ Behavioural & social sciences ☐ Ecological, evolutionary & environmental sciences

For a reference copy of the document with all sections, see nature.com/documents/nr-reporting-summary-flat.pdf

Life sciences study design

All studies must disclose on these points even when the disclosure is negative.

Sample size	The sample size (n>=3) of each experiment is included in the corresponding figure captions and Methods section in the main manuscript and supplementary information files. Sample sizes were selected to ensure that they are sufficient for statistical comparison between different experimental groups.
Data exclusions	No data was excluded from any of the analyses in this work.
Replication	All in vitro experiments were repeated independently for at least 3 times. All in vivo experiments were repeated with at least 3 mice: 3 or 5 mice per group for analyzing the immune cells, and 5 or 7 mice per group for monitoring the tumor growth and survival periods. This information is also given in the figure legends and Methods section.
Randomization	For all studies, samples were randomly divided into different experimental groups.
Blinding	No formal blinding was used throughout experiments. The investigators should keep careful track of protocols because that most of the experiments needed multiple treatments (including formulation, cells or mouse tumor treatment, sample collection, and so on). Hence, it would be difficult to blind the investigators to group allocation during data collection and analysis. The bioluminescence imaging study was conducted by an independent operator, who was unaware of the treatment conditions.

Reporting for specific materials, systems and methods

We require information from authors about some types of materials, experimental systems and methods used in many studies. Here, indicate whether each material, system or method listed is relevant to your study. If you are not sure if a list item applies to your research, read the appropriate section before selecting a response.

Materials & experimental systems

n/a	Involved in the study	
	☒	Antibodies
	☒	Eukaryotic cell lines
☒		Palaeontology and archaeology
	☒	Animals and other organisms
☒		Clinical data
☒		Dual use research of concern

Methods

n/a	Involved in the study	
☒		ChIP-seq
	☒	Flow cytometry
☒		MRI-based neuroimaging

Antibodies

Antibodies used

1. Antitumour experiments: anti-PD-L1 antibody (BioXCell, catalogue number BE0101, Clone: 10F.9G2).
2. Flow cytometry analysis: FITC anti-mouse CD11c (BioLegend, catalogue number 117306, clone: N418, dilution: 1:200), PE anti-mouse CD80 (BioLegend, catalogue number 104708, clone: 16-10A1, dilution: 1:200), APC anti-mouse CD86 (BioLegend, catalogue number 105012, clone: GL-1, dilution: 1:200), FITC anti-mouse CD45 (BioLegend, catalogue number 103108, clone: 30-F11, dilution: 1:200), APC anti-mouse/human CD11b (BioLegend, catalogue number 101212, clone: M1/70, dilution: 1:200), PE anti-mouse Gr-1 (BioLegend, catalogue number 108408, clone: RB6-8C5, dilution: 1:200), FITC anti-mouse/human CD11b (BioLegend, catalogue number 101206, clone: M1/70, dilution: 1:200), APC anti-mouse F4/80 (BioLegend, catalogue number 123116, clone: BM8, dilution: 1:200), PE anti-mouse CD206 (BioLegend, catalogue number 141706, clone: C068C2, dilution: 1:200), APC anti-mouse CD3 (BioLegend, catalogue number 100236, clone: 17A2, dilution: 1:200), PE anti-mouse CD8a (BioLegend, catalogue number 100708, clone: 53-6.7, dilution: 1:200), PE anti-mouse CD335 (BioLegend, catalogue number 137604, clone: 29A1.4, dilution: 1:200), PerCP anti-mouse CD8a (BioLegend, catalogue number 100732, clone: 53-6.7, dilution: 1:200), PE anti-mouse CD44 (BioLegend, catalogue number 397504, clone: C44Mab-5, dilution: 1:200) and APC anti-mouse CD62L (BioLegend, catalogue number 104412, clone: MEL-14, dilution: 1:200).
3. Western blot: anti-HSF1 antibody (Abcam, catalogue number ab52757, clone: EP1710Y, dilution: 1:1000), anti-HSP70 antibody (Abcam, catalogue number ab181606, clone: EPR16892, dilution: 1:1000), anti-Cleaved Caspase-3 antibody (CST, catalogue number 9664S, dilution: 1:1000), anti-β-actin antibody (Abclonal, catalogue number AC026, clone: ARC5115-01, dilution: 1:5000), anti-GAPDH antibody (Abclonal, catalogue number AC033, clone: AMC0062, dilution: 1:5000), anti-Histone H3 antibody (Abcam, catalogue number ab1791, dilution: 1:1000), anti-TRPV1 antibody (Proteintech, catalogue number 66983-1-Ig, clone: 1A3C9, dilution: 1:500), goat anti-rabbit IgG H&L secondary antibody (Abcam, catalogue number ab6702, dilution: 1:5000) and goat anti-mouse IgG H&L secondary antibody (Abcam, catalogue number ab6708, dilution: 1:5000).
4. Immunostaining: anti-HSF1 antibody (Abcam, catalogue number ab52757, clone: EP1710Y, dilution: 1:100), anti-HSP70 antibody (Abcam, catalogue number ab181606, clone: EPR16892, dilution: 1:100), anti-TRPV1 antibody (Proteintech, catalogue number 66983-1-Ig, clone: 1A3C9, dilution: 1:100), anti-CD31 antibody (Abcam, catalogue number ab281583, clone: RM1006, dilution: 1:100), anti-VEGF-A antibody (Abcam, catalogue number ab52917, clone: EP1176Y, dilution: 1:100), anti-TGFβ1 antibody (Abclonal, catalogue number A15103, dilution: 1:100), anti-α-SMA antibody (Abclonal, catalogue number A2319, clone: ARC1913, dilution: 1:100), anti-FAPα antibody (Abclonal, catalogue number A6349, dilution: 1:100), anti-collagen I antibody (Abclonal, catalogue number A5786, dilution: 1:100), anti-fibronectin antibody (Abclonal, catalogue number A16678, dilution: 1:100), anti-Ki67 antibody (Abcam, catalogue number ab15580, dilution: 1:100), Alexa 594 labelled goat anti-rabbit IgG H&L secondary antibody (Abcam, catalogue number ab150080, dilution: 1:200), Alexa 594 labelled goat anti-mouse IgG H&L secondary antibody (Abcam, catalogue number ab150116, dilution: 1:200), Alexa 488 labelled goat anti-rabbit IgG H&L secondary antibody (Abcam, catalogue number ab150077, dilution: 1:200) and HRP labelled goat anti-rabbit IgG H&L secondary antibody (ThermoFisher, catalogue number 32460, dilution: 1:3000).

Validation

Antibodies used were commercially available and all antibodies were validated by manufacturers, with related data shown on the manufacturer's website.
1. Antitumour experiments:
anti-PD-L1 antibody (BioXCell, catalogue number BE0101, Clone: 10F.9G2)
https://www.bioxcell.com/invivomab-anti-mouse-pd-l1-b7-h1-be0101
2. Flow cytometry analysis
anti-mouse CD11c FITC (BioLegend, catalogue number 117306, clone: N418)
https://www.biolegend.com/en-us/products/fitc-anti-mouse-cd11c-antibody-1815?GroupID=BLG11937
anti-mouse CD80 PE (BioLegend, catalogue number 104708, clone: 16-10A1)
https://www.biolegend.com/en-us/products/pe-anti-mouse-cd80-antibody-43?GroupID=BLG1851
anti-mouse CD86 APC (BioLegend, catalogue number 105012, clone: GL-1)
https://www.biolegend.com/en-us/products/apc-anti-mouse-cd86-antibody-2896?GroupID=BLG10719
anti-mouse CD45 FITC (BioLegend, catalogue number 103108, clone: 30-F11)
https://www.biolegend.com/en-us/products/fitc-anti-mouse-cd45-antibody-99?GroupID=BLG1932
anti-mouse/human CD11b APC (BioLegend, catalogue number 101212, clone: M1/70)
https://www.biolegend.com/en-gb/products/apc-anti-mouse-human-cd11b-antibody-345?GroupID=BLG10530
anti-mouse Gr-1 PE (BioLegend, catalogue number 108408, clone: RB6-8C5)
https://www.biolegend.com/en-us/products/pe-anti-mouse-ly-6g-ly-6c-gr-1-antibody-460?GroupID=BLG4876
anti-mouse/human CD11b FITC (BioLegend, catalogue number 101206, clone: M1/70)
https://www.biolegend.com/en-us/products/fitc-anti-mouse-human-cd11b-antibody-347?GroupID=BLG10660
anti-mouse F4/80 APC (BioLegend, catalogue number 123116, clone: BM8)
https://www.biolegend.com/en-us/products/apc-anti-mouse-f4-80-antibody-4071?GroupID=BLG5319
anti-mouse CD206 PE (BioLegend, catalogue number 141706, clone: C068C2)
https://www.biolegend.com/en-us/search-results/pe-anti-mouse-cd206-mmr-antibody-7424?GroupID=BLG9506
anti-mouse CD3 APC (BioLegend, catalogue number 100236, clone: 17A2)
https://www.biolegend.com/en-us/products/apc-anti-mouse-cd3-antibody-8055?GroupID=BLG242

anti-mouse CD8a PE (BioLegend, catalogue number 100708, clone: 53-6.7)
https://www.biolegend.com/en-us/products/pe-anti-mouse-cd8a-antibody-155?GroupID=BLG2559
anti-mouse CD335 PE (BioLegend, catalogue number 137604, clone: 29A1.4)
https://www.biolegend.com/en-us/products/pe-anti-mouse-cd335-nkp46-antibody-6523?GroupID=BLG8849
anti-mouse CD8a PerCP (BioLegend, catalogue number 100732, clone: 53-6.7)
https://www.biolegend.com/en-us/products/percp-anti-mouse-cd8a-antibody-4256?GroupID=BLG6765
anti-mouse CD44 PE (BioLegend, catalogue number 397504, clone: C44Mab-5)
https://www.biolegend.com/en-us/products/apc-anti-human-cd44-antibody-18191?GroupID=GROUP28
anti-mouse CD62L APC (BioLegend, catalogue number 104412, clone: MEL-14)
https://www.biolegend.com/en-us/products/apc-anti-mouse-cd62l-antibody-381?GroupID=BLG10670
3. Western blot
anti-HSF1 antibody (Abcam, catalogue number ab52757, clone: EP1710Y)
https://www.abcam.cn/products/primary-antibodies/hsf1-antibody-ep1710y-chip-grade-ab52757.html
anti-HSP70 antibody (Abcam, catalogue number ab181606, clone: EPR16892)
https://www.abcam.com/products/primary-antibodies/hsp70-antibody-epr16892-ab181606.html
anti-Cleaved Caspase-3 antibody (CST, catalogue number 9664S)
https://www.cellsignal.com/products/primary-antibodies/cleaved-caspase-3-asp175-5a1e-rabbit-mab/9664
anti-β-actin antibody (Abclonal, catalogue number AC026, clone: ARC5115-01)
https://abclonal.com/catalog-antibodies/ACTBMonoclonalAntibody/AC026
anti-GAPDH antibody (Abclonal, catalogue number AC033, clone: AMC0062)
https://abclonal.com/catalog-antibodies/GAPDHMonoclonalAntibody/AC033
anti-Histone H3 antibody (Abcam, catalogue number ab1791)
https://www.abcam.com/products/primary-antibodies/histone-h3-antibody-nuclear-marker-and-chip-grade-ab1791.html
anti-TRPV1 antibody (Proteintech, catalogue number 66983-1-Ig, clone: 1A3C9)
https://www.ptglab.co.jp/Products/TRPV1-Antibody-66983-1-Ig.htm
goat anti-rabbit IgG H&L secondary antibody (Abcam, catalogue number ab6702)
https://www.abcam.cn/products/secondary-antibodies/goat-rabbit-igg-hl-ab6702.html
goat anti-mouse IgG H&L secondary antibody (Abcam, catalogue number ab6708)
https://www.abcam.cn/products/secondary-antibodies/goat-mouse-igg-hl-ab6708.html
4. Immunostaining
anti-HSF1 antibody (Abcam, catalogue number ab52757, clone: EP1710Y)
https://www.abcam.cn/products/primary-antibodies/hsf1-antibody-ep1710y-chip-grade-ab52757.html
anti-HSP70 antibody (Abcam, catalogue number ab181606, clone: EPR16892)
https://www.abcam.com/products/primary-antibodies/hsp70-antibody-epr16892-ab181606.html
anti-TRPV1 antibody (Proteintech, catalogue number 66983-1-Ig, clone: 1A3C9)
https://www.ptglab.co.jp/Products/TRPV1-Antibody-66983-1-Ig.htm
anti-CD31 antibody (Abcam, catalogue number ab281583, clone: RM1006)
https://www.abcam.cn/products/primary-antibodies/cd31-antibody-rm1006-ab281583.html
anti-VEGF-A antibody (Abcam, catalogue number ab52917, clone: EP1176Y)
https://www.abcam.cn/products/primary-antibodies/vegfa-antibody-ep1176y-c-terminal-ab52917.html
anti-TGFβ1 antibody (Abclonal, catalogue number A15103)
https://abclonal.com/catalog-antibodies/TGFB1RabbitpAb/A15103
anti-α-SMA antibody (Abclonal, catalogue number A2319, clone: ARC1913)
https://abclonal.com/catalog-antibodies/Actin1ACTA1RabbitmAb/A2319
anti-FAPα antibody (Abclonal, catalogue number A6349)
https://abclonal.com/catalog-antibodies/FibroblastactivationproteinFAPRabbitpAb/A6349
anti-collagen I antibody (Abclonal, catalogue number A5786)
https://abclonal.com/catalog-antibodies/CollagenICOL1A2RabbitpAb/A5786
anti-fibronectin antibody (Abclonal, catalogue number A16678)
https://abclonal.com/catalog-antibodies/FibronectinPolyclonalAntibody/A16678
anti-Ki67 antibody (Abcam, catalogue number ab15580)
https://www.abcam.cn/products%2fprimary-antibodies%2fki67-antibody-ab15580.html
Alexa 594 labelled goat anti-rabbit IgG H&L secondary antibody (Abcam, catalogue number ab150080)
https://www.abcam.cn/products/secondary-antibodies/goat-rabbit-igg-hl-alexa-fluor-594-ab150080.html
Alexa 594 labelled goat anti-mouse IgG H&L secondary antibody (Abcam, catalogue number ab150116)
https://www.abcam.cn/products/secondary-antibodies/goat-mouse-igg-hl-alexa-fluor-594-ab150116.html
Alexa 488 labelled goat anti-rabbit IgG H&L secondary antibody (Abcam, catalogue number ab150077)
https://www.abcam.cn/products/secondary-antibodies/goat-rabbit-igg-hl-alexa-fluor-488-ab150077.html
HRP labelled goat anti-rabbit IgG H&L secondary antibody (ThermoFisher, catalogue number 32460)
https://www.thermofisher.cn/cn/zh/antibody/product/Goat-anti-Rabbit-IgG-H-L-Secondary-Antibody-Polyclonal/32460

Eukaryotic cell lines

Policy information about cell lines and Sex and Gender in Research

Cell line source(s)

A549 (catalogue number SCSP-503), HepG2 (catalogue number SCSP-510), MDA-MB-231 (catalogue number SCSP-5043), HCT-116 (catalogue number SCSP-5076), and PANC1 (catalogue number SCSP-535) cells were purchased from Cell Bank of Type Culture Collection of the Chinese Academy of Sciences (Shanghai, China). 4T1 tagged with luciferase (catalogue number IML-021), HCT-116 tagged with luciferase (catalogue number IML-052), PANC02 (catalogue number IML-092) and PANC02 tagged with luciferase (catalogue number IML-045) cells were obtained from IMMOCELL. A549-TRPV1 KD cell was constructed by transfection of TRPV1 shRNA (5`-CCG AGG GAT TCA GTA TTT CCT-3`) (GeneCopoeia, Rockville, MD) into A549-WT cells using lipofectamine 2000 as the vector and further incubation with 1.0 μg mL-1 puromycin for picking up A549-TRPV1 KD cells. A549-TRPV1 cells were constructed by transient transfection of TRPV1 plasmid (a kind gift from Prof. Jiuping Ding, Huazhong University of Science and Technology, Wuhan, China) into A549-WT cells using lipofectamine 2000.

Authentication	Each cell line we used was morphologically confirmed according to the information provided by the cell-source center. Western blot was applied to verify the successful construction of A549-TRPV1 KD and A549-TRPV1 cells.
Mycoplasma contamination	Cells were tested negative for mycoplasma contamination by using the MycAway-Color one-step mycoplasma detection kit.
Commonly misidentified lines (See ICLAC register)	No commonly misidentified cell lines were used.

Animals and other research organisms

Policy information about studies involving animals; ARRIVE guidelines recommended for reporting animal research, and Sex and Gender in Research

Laboratory animals	BALB/c mice (female, 18 ± 2 g, 6-8 weeks), BALB/c nude mice (female, 18 ± 2 g, 6-8 weeks) and C57BL/6J mice (female, 18 ± 2 g, 6-8 weeks) were purchased from Shanghai SLAC Animal Technology Co., Ltd. (Shanghai China). Mice were housed in an animal facility under constant environmental conditions (room temperature, 21 ± 1°C; relative humidity, 40-70% and a 12 h light-dark cycle). All mice had access to food and water.
Wild animals	The study did not involve wild animals.
Reporting on sex	The experiment was designed without considering the sex of the mice, and female mice were selected to ensure gender uniformity.
Field-collected samples	The study did not involve samples collected from the field.
Ethics oversight	All animal procedures were performed with ethical compliance and approved by the Animal Care and Use Committee of Soochow University with the approval number of ECSU-2019000179.

Note that full information on the approval of the study protocol must also be provided in the manuscript.

Flow Cytometry

Plots

Confirm that:

☒ The axis labels state the marker and fluorochrome used (e.g. CD4-FITC).

☒ The axis scales are clearly visible. Include numbers along axes only for bottom left plot of group (a 'group' is an analysis of identical markers).

☒ All plots are contour plots with outliers or pseudocolor plots.

☒ A numerical value for number of cells or percentage (with statistics) is provided.

Methodology

Sample preparation	The lymph nodes were first mechanically disrupted and then filtered through a 40-µm cell strainer to isolate the cells. The spleen and tumours were first lysed by red-blood-cell lysis buffer and then centrifuged for further analysis.
Instrument	BD, FACSAria III
Software	BD FACSDivaTM software was used for data acquisition and FlowJo V10 was used for data analysis.
Cell population abundance	The relative abundance was maintained by diluting all the samples at equal volume and collecting samples at a fixed and consistent time. Representative plots for each group are shown in main figures and supplementary figures.
Gating strategy	Generally, cells were first gated on FSC/SSC. Singlet cells were usually gated using FSC-H and FSC-A. Surface antigen-positive cells were gated according to the cells stained with single antibody.

☒ Tick this box to confirm that a figure exemplifying the gating strategy is provided in the Supplementary Information.
